# Introgression among North American wild grapes (*Vitis*) fuels biotic and abiotic adaptation

**DOI:** 10.1186/s13059-021-02467-z

**Published:** 2021-09-03

**Authors:** Abraham Morales-Cruz, Jonas A. Aguirre-Liguori, Yongfeng Zhou, Andrea Minio, Summaira Riaz, Andrew M. Walker, Dario Cantu, Brandon S. Gaut

**Affiliations:** 1 Department of Ecology and Evolutionary Biology, University of California Irvine, Irvine, CA USA; 2 Department of Viticulture and Enology, University of California, Davis, Davis, CA USA

**Keywords:** Adaptive introgression, Pierce’s disease, Grapevines, Climate

## Abstract

**Background:**

Introgressive hybridization can reassort genetic variants into beneficial combinations, permitting adaptation to new ecological niches. To evaluate evolutionary patterns and dynamics that contribute to introgression, we investigate six wild *Vitis* species that are native to the Southwestern United States and useful for breeding grapevine (*V. vinifera*) rootstocks.

**Results:**

By creating a reference genome assembly from one wild species, *V. arizonica*, and by resequencing 130 accessions, we focus on identifying putatively introgressed regions (pIRs) between species. We find six species pairs with signals of introgression between them, comprising up to ~ 8% of the extant genome for some pairs. The pIRs tend to be gene poor, located in regions of high recombination and enriched for genes implicated in disease resistance functions. To assess potential pIR function, we explore SNP associations to bioclimatic variables and to bacterial levels after infection with the causative agent of Pierce’s disease (*Xylella fastidiosa*). pIRs are enriched for SNPs associated with both climate and bacterial levels, suggesting that introgression is driven by adaptation to biotic and abiotic stressors.

**Conclusions:**

Altogether, this study yields insights into the genomic extent of introgression, potential pressures that shape adaptive introgression, and the evolutionary history of economically important wild relatives of a critical crop.

**Supplementary Information:**

The online version contains supplementary material available at 10.1186/s13059-021-02467-z.

## Background

Species emerge from complex interactions among evolutionary processes. For example, genetic drift and local adaptation drive divergence between populations, which ultimately leads to genetic isolation and eventual speciation [1, 2]. The process of divergence can be slowed, in turn, by gene flow between populations, which maintains genetic similarity. There is growing evidence, however, that introgressive hybridization between populations and species does more than homogenize gene pools. It may also be a source of novelty that reassorts genetic variants into beneficial combinations, permitting adaptation to new ecological niches [3]. This ability to reassort genetic variation may partially explain the highly reticulated evolutionary history of adaptive radiations like *Heliconius* butterflies [4], tomatoes [5], Darwin’s finches [6], and African cichlids [7]. Introgression has also played a major role in the diversification and speciation of angiosperms [8], because hybridization affects an estimated ~ 25% of flowering plant species [9].

It is generally not known how frequently introgression occurs between species, whether introgression events are adaptive, and, if so, the traits that have been affected. Fortunately, genomic approaches have begun to provide some insights into these central questions. For example, the analysis of *Heliconius* genomes suggests that a large inversion was transferred between species and that this event was adaptive because the inversion contains a color pattern locus that controls mimicry and crypsis [4]. Similarly, a large chromosomal region was exchanged between distinct sunflower subspecies, likely facilitating genetic adaptation to xeric environments [10]. Recent work in cypress [11], oaks [12, 13], maize [14], and cultivated date palms [15] also suggest that introgression between plant species facilitates adaptation to local environments. The work in maize and date palms further highlights the importance of studying the wild relatives of crop species, because they are potential sources of traits for agronomic improvement [16]. Indeed, numerous studies have documented introgression between a crop and its wild relative, leading to a growing understanding of how introgression contributes to important agronomic traits like highland adaptation in maize [14], stress tolerance in potatoes [17], and perhaps fruit quality in apple [18]. We nonetheless emphasize that for many genomic studies of introgression among wild species, the potential phenotypic basis for adaptation has been either unclear or unstudied.

Here we explore the genomic extent and evolutionary dynamics of introgression among wild relatives of cultivated grapevines (*Vitis vinifera* ssp. *sativa*). The genus *Vitis* likely originated in North America ~ 45 my [19] and contains two subgenera [*Muscadinia* (2*n* = 40) and *Vitis* (2*n* = 38)] that encompass ~ 70 species across varied environments. The subgenus *Vitis* has a disjunct distribution across North America and Eurasia [19], with the ~ 25 North American species [20] distributed broadly across the continent, including the American Southwest, where extreme temperature changes and drought are pervasive abiotic stressors. All species within the subgenus are dioecious, interfertile, and often sympatric [21], suggesting the possibility of an extensive history of introgression among species [22, 23]. However, the extent and genomic location of introgressed regions remain unexplored among *Vitis* species, as do the potential functions and evolutionary forces that may shape successful introgression events.

*Vitis* is also an important study system because cultivated *V. vinifera* (hereafter *vinifera*) is the most valuable horticultural crop in the world [24] and also because it is a model for the study of perennial fruit crops [25]. It is not always appreciated, however, that the cultivation, sustainability, and security of grapevine cultivation relies on North American (NA) *Vitis* species as rootstocks that provide resistance to abiotic and biotic stress [21, 26, 27]. There is a need to identify additional sources of resistance to biotic and abiotic stress, however, because the major rootstock cultivars currently utilized represent a narrow genetic foundation [28]. One biotic stress is Pierce’s disease (PD), which is a global threat to the sustainability of wine production [29]. PD is caused by a bacterium (*Xyllela fastidiosa*) that spreads from plant to plant by xylem-feeding insect vectors. Several *Vitis* species are polymorphic for resistance to PD, including *V. arizonica* [30] and other species native to the Southwestern United States [31]. These observations open interesting questions about the potential introgression of pathogen resistance loci among wild grape species.

**Fig. 1:**
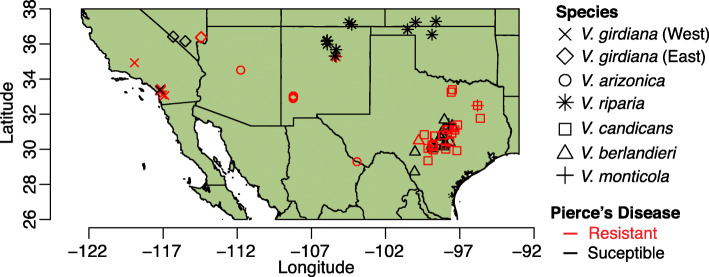
Geographic distribution of sampled populations of wild grapes. Shapes correspond to different genetic clusters. Samples colored red or black were classified as resistant or susceptible to Pierce’s disease, respectively

In this study, our goal is to characterize the genomic extent of introgression among wild *Vitis* species from the American Southwest. To do so, we have assembled a reference genome from one species (*V. arizonica*) and generated whole-genome resequencing data from 130 accessions representing six *Vitis* species from portions of their native ranges (Fig. 1). Some of these species have largely overlapping distributions (e.g., *V candicans* and *V. berlandieri* in Texas), others have disjunct distributions (e.g., *V. arizonica*), and still another (*V. riparia*) has populations in the Southwest at the edge of a broader continental distribution. To complement our genetic data, we have also assessed PD resistance for each accession and gathered bioclimatic data from their location of origin. Given this multifaceted dataset, we address four sets of questions. First, given that species are interfertile and can overlap substantively in geographic range, are they genetically distinct? Second, if they are distinct, is there nonetheless genetic evidence for introgression? Third, if there is evidence for introgression, what are the genomic characteristics of introgressed regions, in terms of locations, size, and gene content? Finally, is there evidence that introgression events have played an adaptive role, as evidenced by genetic associations with either disease resistance or bioclimatic variables? In addressing these questions, this study provides novel insights into the evolutionary dynamics that shape the radiation of *Vitis* and identifies potentially useful genomic targets for breeding.

## Results

### Population structure and phylogeny

**Fig. 2:**
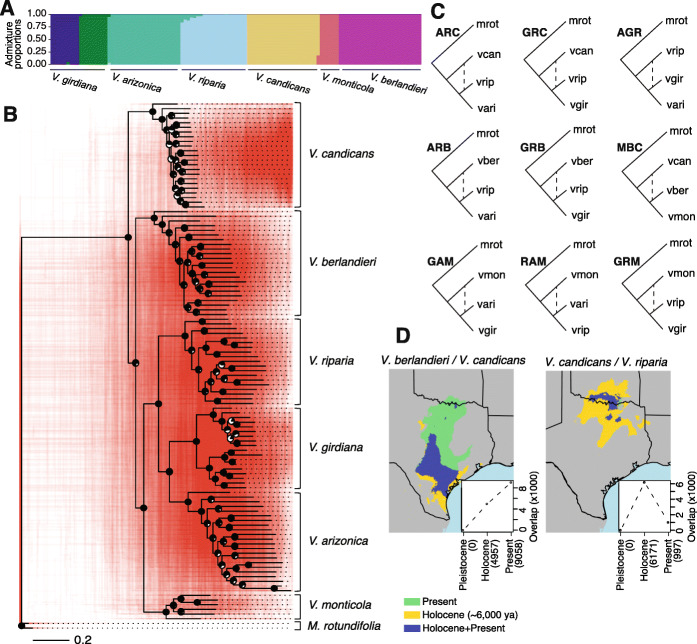
Genetic history of the wild grapes sampled. **A** Genetic structure of samples detected by the structure analysis (*K* = 7). Hybrid samples are not included but see Additional File 2: Figure S1. **B** The phylogenetic tree in black corresponds to the consensus tree. Each node has a pie chart with the black portion indicating the proportion of supporting bootstrap replicates. The red phylogenies in the background correspond to 500 highly supported consensus trees (median bootstrap support > 70%) based on separate 10-kb windows throughout the genome. The scale bar represents 0.2 average substitutions per nucleotide. **C** Diagram of the tree models used for the nine trios that had significant introgression signals, structured from top to bottom of each tree as follows: outgroup, P3, P2, and P1. In this diagram, the species are abbreviated as mrot: *M. rotundifolia*, vari: *V. arizonica*, vcan: *V. candicans*, vmon: *V. monticola*, vber: *V. berlandieri*, vrip: *V. riparia* and vgir: *V. girdiana.* **D** Examples of SDM overlaps from pairs of species with evidence of introgression projected in one of three periods: Pleistocene, Holocene and the Present. The inset in the bottom left corner shows the area of overlap per period. The overlap corresponds to the number of overlapped pixels from the raster objects at a 2.5-arcsecond resolution. See Additional File 2: Figures S5, S6 and S7 for additional SDMs featuring pairs of species

We generated a reference genome of *V. arizonica* from previously reported long-read sequences [32] that we assembled with the aid of a new optical map. The reference assembly contained 19 anchored pseudomolecules, an N50 of 25.9 Mb, a size of 503 Mb, and a BUSCO score of 96.4% (Additional File 1: Table S1 & Table S2). We generated short- and long-read RNAseq data to annotate genes within the genome, ultimately predicting 28,259 gene models. We then resequenced the genome of 130 *Vitis* samples from throughout the native range of six species (*V. arizonica*, *V. berlandieri*, *V. candicans*, *V. girdiana*, *V. monticola*, and *V. riparia*) (Fig. 1, Additional File 1: Table S3). After mapping resequencing data to the reference, we identified ~ 20 million SNPs among all samples (Additional File 1: Table S4) and used them to assess genetic structure using NGSadmix [33] with *K* = 2 to 10 clusters. The highest support was for *K* = 7 clusters, corresponding to one per species, except for *V. girdiana*, which had two disjunct groups from different geographical locations (Figs. 1 and 2A, Additional File 2: Figure S1). Based on NGSadmix results, we found and removed 19 hybrid individuals that had < 80% of the admixture proportion assigned to a single cluster [34, 35], leaving a final dataset of 111 accessions. Of these, four individuals (“vber09,” “vrip15,” “vcan26,” and “vcan27”) did not fit neatly into their initially proposed species group based on our genetic clustering; we treated each of the four as mis-named and assigned them to a species based on genetic grouping. Altogether, the 111 samples included accessions from *V. arizonica* (*n* = 22), *V. candicans* (*n* = 24), *V. berlandieri* (*n* = 22), *V. girdiana* (*n* = 18), *V. riparia* (*n* = 19), and *V. monticola* (*n* = 6) (Fig. 2A). Genome-wide nucleotide diversity per base pair (*π*) averaged 0.00284 across species and ranged from 0.00211 in *V. berlandieri* to 0.00353 in *V. monticola* (Additional File 1: Table S5).

We created a consensus phylogenetic tree based on a reduced number of SNPs that limit the effects of linkage disequilibrium (see “Methods”). The phylogeny had median bootstrap support of 88.5% for all nodes and strong support (> 76%) for nodes that separated species groups (Fig. 2B, Additional File 2: Figure S2). In addition, the accessions from each species formed a well-supported monophyletic clade, demonstrating that species are genetically identifiable and also justifying treating each named species as a separate group. Given the phylogeny, we calculated divergence times using a calibration point of 28.32 million years [22] for the separation of *M. rotundifolia* from the *Vitis* subgenus (Additional File 2: Figure S3). Divergence time estimates indicated that the deepest node of individual species often dated to ~ 10 million years or older, but the divergence between species typically exceeded 20 million years (Additional File 2: Figure S3). Despite strong support for the consensus tree, phylogenies based on individual 10Kb genomic regions from throughout the genome were highly discordant, representing “clouds” around species (Fig. 2B). These clouds illustrate potentially reticulate lineages and suggest the possibility of incomplete lineage sorting and/or a history of introgression among species.

### Tests for introgression and geographic overlap among species

To formally test for introgression, we calculated the *D* statistic [36–38]. *D* is a genome-wide statistic that measures the excess of shared ancestral alleles based on a tree model of four populations, designated as (((P1,P2),P3),O), where P1, P2, and P3 are ingroups and O is the outgroup. *D* is expected to be zero under the null hypothesis of incomplete lineage sorting but deviates from zero when there is introgression between P2 and P3 [36, 37, 39]. We calculated *D* for all combinations of three *Vitis* ingroup species (hereafter called “trios”) that had an appropriate topology for the test according to the consensus tree (Fig. 2B, Additional File 1: Table S6). We used *Muscadinia rotundifolia* as the outgroup for all trios and estimated significance using a block jackknife approach [37]. Of 11 trios tested, nine had a significant *D* value at *p* < 0.0031 (Additional File 1: Table S6), representing a total of six P2-P3 species pairs (Fig. 2C, Table 1).

**Table 1:** Sets of taxa with significant D statistics and properties of the pIRs

Trio^**a**^	P2	P3	***f***_**4**_-ratio	Mean pIRs (Kb)^**b**^	No. genes	Gene density^**c**^	Sweeps^**d**^	cM/kb^**e**^	Resistance genes^**f**^	PD^**g**^	Climate^**h**^
GAM	*arizonica*	*monticola*	8.03%	269.36	2282	0.96	0.27	2.27	1.1	2.19*	1.27*
RAM	*arizonica*	*monticola*	2.07%	210.44	597	0.92	0	1.1	0.56	1.12	1.51*
MBC	*berlandieri*	*candicans*	3.24%	163.23	746	0.82*	0.39	1.52	1.12	0.59	2.02*
AGR	*girdiana*	*riparia*	3.32%	154.49	955	0.99	0.32	1.61	0.52	0.80	1.42*
GRM	*riparia*	*monticola*	6.08%	270.68	1325	0.79*	0.57	1.21	1.56*	0.81	1.13
GRB	*riparia*	*berlandieri*	7.47%	233.29	1538	0.75*	0.93	1.43	2.00*	1.03	1.13*
ARB	*riparia*	*berlandieri*	7.40%	200.07	1607	0.75*	0.77	1.8	1.73*	1.09	1.28*
ARC	*riparia*	*candicans*	2.43%	226.25	480	0.77*	1.41*	1.23	3.55*	2.47*	1.14*
GRC	*riparia*	*candicans*	2.30%	217.32	455	0.76*	0.43	1.45	3.01*	2.47*	1.33*

^a^The three letters in a trio represent three species that act as P1, P2, and P3, respectively, in introgression tests. The species are A: *V. arizonica*, B: *V. berlandieri*, C: *V. candicans*, G: *V. girdiana*, M: *V. monticola*, and R: *V. riparia*. For each trio, *M. rotundifolia* was used as the outroup for introgression tests

^b^ The mean length of pIRs after merging windows separated by < 1 kb in distance

^c^Gene density inside pIRs relative to the average gene density of the whole genome. Asterisks denote trios with significantly lower values of gene density in pIRs (*p* < 0.05)

^d^Sweeps refers to the relative enrichment of selective sweeps in pIR regions. Significant enrichment is denoted by an asterisk, with significance (*p* < 0.05) assessed by permutation

^e^cM/kb refers to the ratio of the average recombination rate within pIR regions, relative to the genomic background

^f^ Resistance genes refers to the enrichment of annotated disease resistance genes. Asterisks denote trios with significant enrichment (*p* < 0.05)

^g^ PD refers to the enrichment of Pierce’s disease associated SNPs within pIRs. Significant enrichment is denoted by an asterisk (*p* < 0.05), with significance assessed by permutation

^h^ Climate refers to the enrichment of bioclimate associated SNPs within pIRs. Values correspond to the average relative enrichment of the top three bioclimatic variables. Significant enrichment is denoted by an asterisk, with significance assessed by permutation (*p* < 0.05)

The *D* statistic is useful for detecting introgression but a poor estimator of the introgressed proportion of genome [39, 40], so we applied the *f*_4_-ratio [37] to estimate the proportion of the genome with the signal of introgression. The values ranged from ~ 2.3% of the genome in comparisons between *V. riparia* and *V. candicans*, to ~ 8.0% between *V. arizonica* and *V. monticola* (Table 1)*.* For some comparisons, we were able to test the same species pairs with different “control” P1 species. In two cases, the same species pairs yielded similar estimates (Table 1) providing some reassurance about the results. In contrast, the *f*_4_-ratio estimate varied widely, from 2.1 to 8.0%, for tests between *V. monticola* and *V. arizonica* depending on the P1 control species; notably, however, *D* was significant with either control. Following calculation of the *f*_4_-ratio, we also calculated the *f*-branch statistic [41], which recognizes that trios are not independent because they share branches. The *f*-branch statistic did not yield significant values for any of the internal branch nodes (Additional File 2: Figure S4), suggesting that introgression signals are due to multiple recurrent events instead of a single ancestral event. Taken together, these results show that (*i*) *D* and *f*_4_-ratio support historical introgression among species, (*ii*) the history of introgression is complex, potentially involving multiple species pairs and multiple events, and (*iii*) *V. riparia* was most commonly implicated in introgression events with other species (Fig. 2C).

A puzzling feature of these results is that some P2-P3 species pairs currently have few or no regions of geographic overlap (Figure S5; Additional File 2: Figure S5), suggesting limited opportunities for hybridization. To explore the potential for sympatry between species, we performed species distribution modeling (SDM). SDMs identify climatic factors that define the geographic distribution of a species and predict the change in species’ distribution over time, given climate prediction models [42–44]. We constructed SDMs based on bioclimatic data from the present, the Holocene (~ 6000 ya), and the Pleistocene (~ 18,000 ya) (see “Methods”) (Fig. 2D). Some of the species pairs had no predicted geographic overlap in any of the three periods (Additional File 2: Figure S6), and none of these yielded genetic evidence for introgression. Others had overlapping distributions but without detected introgression events (Additional File 2: Figure S7). Finally, all species pairs with genomic evidence for introgression had some geographic overlap (Additional File 2: Figure S5). In some cases, however, the predicted geographic overlap between species was higher in the past. For example, *V. candicans* and *V. riparia* had little predicted overlap in the present and in the Pleistocene, but substantial predicted overlap in the Holocene (Fig. 2D). These SDMs support current or historical sympatry of species that yielded evidence for introgression, and they also suggest that detected introgression events are unlikely to have occurred very recently.

### Introgression at chromosomal scales

For each of the nine trios with significant *D* values, we identified putative introgressed chromosomal regions by calculating *f*_d_ [39] and *f*_dM_ [41] in non-overlapping windows of 1000 SNPs along chromosomes (Additional File 2: Figures S8-S11). The two metrics were significantly correlated (*R* > 0.75, *p* < 2.2e−16) along the genome (Additional File 2: Figure S12) and gave qualitatively similar results; we focused on *f*_dM_ because positive values are interpretable as exchange of derived alleles between P2 and P3 [41]. For each trio, we defined putative introgressed regions (pIRs) (Dataset S1) [45] as the top *f*_dM_ windows that summed to the genomic proportion estimated by the *f*_4_-ratio (Table 1).

With pIRs identified, we evaluated their basic characteristics. For example, the mean length of pIRs ranged from 154 kb in the AGR trio (see Table 1 and Fig. 2C for trio definitions) to 271 kb in GRM, with the number of pIRs ranging from 54 to 269 (Table 1). The total complement of pIRs contained from 455 to 2282 genes across trios, representing an estimated 1.6 to 8.1% of all genes. We evaluated GO enrichment for the set of putatively introgressed genes and found 15 terms significantly enriched within pIRs (hypergeometric test, *p* value < 0.048, Additional File 1: Table S7), including cell-cell signaling (GO:0007267), signaling receptor activity (GO:0038023), and nucleotide binding (GO:0000166). However, pIRs had significantly lower gene density per kilobase than the genome-wide average in 6 of 9 trios (Table 1, Additional File 1: Table S8 & Additional File 2: Figure S13), and in no case were pIRs significantly enriched for gene density.

**Fig. 3:**
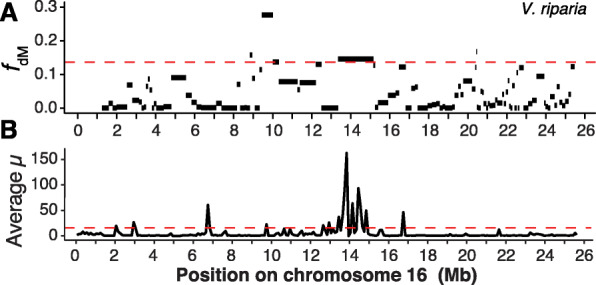
Introgression statistics along chromosome 16 in the ARC trio, which consists of *V. arizonica* as P1, *V. riparia* as P2, and *V. candicans* as P3. **A** Introgression signal measured as *f*_dM_ in windows across the chromosome. Windows within 1000 bp of each other were merged. The red line shows the cutoff value to define pIRs, calculated by highest *x*% of *f*_dM_ values, where *x* was determined for each trio by the *f*_4_-ratio estimate. **B** The *μ* statistic showing potential locations of selective sweeps in *V. riparia*

Finally, we assessed three additional features of pIRs. First, we reasoned that if pIRs result from introgression events, they should not follow the species consensus tree. We therefore investigated the phylogeny of pIRs for each trio using twisst [46], expecting P2 and P3 to be topologically reversed within introgressed regions. Indeed, for each trio the predominant phylogenetic weight was for the tree ((P1,P3),P2,O), as expected if there were introgression between P2 and P3 (Additional File 2: Figure S14). Second, some studies have suggested that introgressed regions should be in genomic regions of high recombination [47]. We used a genetic map to measure recombination in cM/kb [48]; in every trio, pIRs were in regions of higher recombination than the genomic average (Table 1, Additional File 1: Table S9). Third, we sought to determine if pIRs represent adaptive events by assessing their overlap with inferred selective sweeps. To do so, we evaluated sweeps in the P2 population (see “Methods”), focusing on 10-kp windows within the highest 5% *μ* statistic support. On average, 12.2% of pIRs had at least one putative sweep across the trios. However, sweeps were generally not enriched within pIRs; only one trio (ARC) had significantly more sweeps in pIRs than expected at random (*p* < 2.0e^−3^, permutation test) (Table 1). This trio provided an interesting example of a potentially adaptive pIR between *V. riparia* and *V. candicans* on chromosome 16, where the pIR at 14–15 Mb contained multiple putative sweeps (Fig. 3)*.*

### pIRs may be enriched for biotic associations

**Fig. 4:**
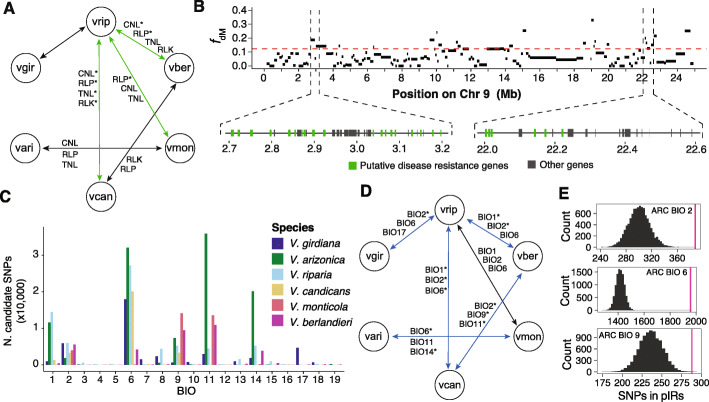
Biotic and abiotic signals in pIRs. **A** Illustrates the pIRs that were either enriched (black arrows) or significantly enriched (green arrows) in at least one category of disease resistance genes, based on permutation of genes across the genome. The four categories of disease resistance genes are CNL: CC-NB-LRR, TNL: TIR-NB-LRR, RLP: Receptor Like Proteins, and RLK: Receptor Like Kinases. Asterisks denote significant categories. **B** Shows windows with introgression signals with pIRs (above the red line) from *V. candicans* into *V. berlandieri* (based on the ARB trio) across chromosome 9. The details show two pIRs that are highly enriched in disease resistance genes in green, with non-disease genes shown in black. **C** Number of SNPs associated with all 19 bioclimatic variables per species. **D** Shows the pIRs that were significantly enriched (blue) in at least one of the top three bioclimatic variables. Asterisks denote significant enrichment in pIRs, based on permutation tests. **E** The diagram shows the distributions of the number of climate-associated SNPs within pIRs based on permutation for the top three bioclimatic variables for the ARC trio. The distributions based on permutations are in black and the observed value is in magenta

pIRs were not enriched for selective sweeps, which suggests superficially that they may not have resulted from recent adaptive events. However, another way to evaluate the potential for adaptation is to focus on function, especially potential roles of pIR genes in biotic resistance. We therefore subjected all 28,259 predicted genes to a pipeline designed to identify disease resistance genes [49] and tallied genes in the four most-studied types of pathogen recognition genes (PRGs)—i.e., CC-NB-LRR (CNL), TIR-NB-LRR (TNL), Receptor Like Proteins (RLP), and Receptor Like Kinases (RLK) genes. We found 208 CNLs, 55 TNLs, 258 RLKs, and 336 RLPs in the *V. arizonica* reference (Dataset S2) [45], representing 3% of all genes. We then assessed whether pIRs were functionally enriched in PRGs among genes. Notably, PRGs were enriched within the pIRs for seven trios, with five significantly enriched (hypergeometric test, *p* value < 3.66e−04) (Fig. 4A, Table 1). Regions introgressed between *V. candicans* and *V. riparia* were especially noteworthy, because all four PRG types were significantly enriched. Illustrative examples include the pIRs at 3 Mb and 22 Mb on chromosome nine, which had 53.7% and 33.3% of their genes annotated as PRGs, respectively (Fig. 4B). The pIR at 14 Mb in chromosome 16 of the ARC trio was another region of interest (Fig. 3), because it contained multiple selective sweeps and had 39.1% of genes annotated as PRGs. Finally, we note that the enrichment of PRGs with pIRs is not due to the confounding effects of recombination. We separated the genome into four quadrants based on recombination rate (high, medium, low, and no recombination) and found that genome-wide PRGs are enriched only in low recombination genomic regions (*p* = 0.0004). Hence, pIRs are rare among high recombination genomic regions because they are enriched for PRGs.

The enrichment for PRGs suggests that pIRs could provide an adaptive benefit to biotic challenges. To pursue this idea further, we used quantitative measurements of *X. fastidiosa* bacterial levels in plant stems after manual infection (see “Methods”). We gathered *X. fastidiosa* data for 108 of the 111 accessions [30, 31] (Fig. 1) and performed genome-wide associations studies (GWAS) using bacterial levels as a quantitative phenotype (Additional File 1: Table S10). Our dataset was not ideal for GWAS because: (*i*) individual species had small sample sizes, which limits statistical power within species and (*ii*) the entire dataset, while much larger, contained accessions from multiple species that reflect strong genetic structure (Fig. 2A). We nonetheless performed GWAS on the entire dataset, with a specific focus on whether pIR regions have enriched numbers of SNPs that associate with variation in bacterial levels.

To analyze the dataset, we applied Latent Factor Mixed Models (LFFM2) [50] while correcting for seven discrete genetic clusters (Fig. 2A) with seven Latent Factors (LFs) (Fig. 2A, Additional File 2: Figure S15A) and using a genome inflation factor to correct for population structure (Additional File 2: Figure S15B-C and Additional File 2: Figure S16). We also used a reduced set of 397,723 SNP sites that had no missing data across all 108 accessions and were polymorphic in at least one species. With this approach, we found bacterial levels were significantly associated (FDR adjusted *p* < 0.05) with 261 SNPs across the genome (Dataset S3, Additional File 2: Figure S17) [45], of which 25% (64/261) were located in pIRs of at least one of the nine trios. We then measured, for each trio, the relative enrichment of SNPs per kilobase within pIRs compared to the remainder of the genome; pIRs had higher densities of associated SNPs in six of nine trios (Table 1). Finally, we assessed the significance of this enrichment using a randomization approach that takes into account SNP densities (see “Methods”); the level of enrichment was significantly higher than expected at random in three of the nine trios (*p* < 3.32e^−2^; permutation test) (Table 1, Additional File 1: Table S11).

Because genetic structure can increase the number of false positives (Additional File 2: Figure S16), we also tested the effect of increasing the number of LFs to further control for population structure. Based on Q-Q plots, we found that increasing the number of LFs reduced the impact of genetic structure, but led to fewer significantly positive associated SNPS—particularly for increasing LF = 7 to LF = 8—but with similar overall patterns (Additional File 2: Figure S18). At LF = 8, there were 129 associated SNPs. Although up to 10% of these outlier SNPs were found within pIRs for some trios, statistical tests for enrichment within pIRs were no longer significant. We suspect that the increase of LFs may better control for genetic structure but likely at the cost of statistical power in both GWAS (see ref. [51]) and enrichment tests. We conclude that pIRs house SNPs associated with bacterial levels and tend to be enriched for such SNPs in some analyses.

### pIRs are enriched for abiotic associations

Given evidence for pIR enrichment for both defense-related genes and for SNPs associated with one biotic interaction (with *X. fastidiosa*), we also performed genome environment associations (GEA) to assess abiotic associations. We used BayPass [52] to identify associations between SNPs and 19 bioclimatic variables for each *Vitis* species separately. Outlier SNPs were defined as having Bayes Factor (BF) > 10 [52, 53]. For each species, we then identified the three bioclimatic variables with the highest number of outlier SNPs (Fig. 4C, Additional File 2: Figure S19). For most species, outlier SNPs were especially associated with low temperatures during the coldest period (BIO11 and BIO6), the temperature of the driest months (BIO9), and/or mean diurnal range (BIO2). The number of candidate SNPs associated with each of the three strongest bioclimatic variables ranged from 3380 SNPs (for BIO9 in *V. candicans*) to 39,587 (for BIO11 in *V. arizonica*) (Dataset S4) [45].

We then assessed whether pIRs in the P2 species were enriched for outlier SNPs, doing so separately for each trio and for each of the three bioclimatic variables. Out of 27 potential combinations (= 3 bioclimatic variables × 9 trios), 16 combinations were enriched for candidate SNPs within pIRs across seven trios (*p* < 2.65e^−2^; permutation test) (Fig. 4E, Additional File 1: Table S12, Dataset S5) [45]. Thus, despite the fact that they tend to represent gene-poor and high recombination regions of the genome, pIRs are enriched for SNPs associated with bioclimatic variables.

## Discussion

Genomic analyses have fueled a growing realization that introgression is an important evolutionary process. These genomic studies have been enhanced by the application of population genomic approaches, like *D* statistics [36, 37] and related metrics. Nonetheless, these studies often suffer from one (or more) of three limitations. First, a surprising number have relied on reduced representation sequencing methods and poor reference genomes [54]. As a consequence, these studies often have the power to identify a signal of introgression but lack the genomic resolution to adequately characterize the location and content of introgressed regions. Second, many—and perhaps most—studies have focused on introgression between a single pair of taxa [54]. While this simplifies analysis and interpretation, it makes it difficult to infer general evolutionary patterns. Finally, many studies lack information about the potential phenotypic effects of introgressed regions [55], although introgression events into a crop from a wild relative constitute notable exceptions [56]. Ultimately, information about potential phenotypic effects are crucial for understanding the evolutionary processes that affect retention of introgressed regions.

In this study, we have used whole genome resequencing data to identify putatively introgress regions (pIRs) across six wild *Vitis* species. *Vitis* is an interesting system for studying introgression because it is an example of an adaptive radiation [19], because several species grow in sympatry (Figs. 1 and 2D & Additional File 2: Figure S5) and because all wild species have the potential to be (or already are) agronomically important. Moreover, all *Vitis* species are inter-fertile, with hybrid individuals found in nature [21]. To facilitate our study of historical introgression, we first generated a reference genome assembly for *V. arizonica.* To date, four genomes have been published from other wild *Vitis* species, two from *V. riparia* [57, 58], *Vitis labrusca* L. [59], and one genome from *V. amurensis* [60]. However, the *V. arizonica* genome is more contiguous than other North American *Vitis* genomes (e.g., scaffold N50 of ~ 1 Mb [57] vs. 25.9 Mb), which presents a clear advantage for its use. Nonetheless, the use of a single genome raises the spector of reference bias. Our use of high coverage (~ 25× average coverage) whole-genome resequencing data should prevent substantial ascertainment biases [61], but reference bias may also influence estimates of heterozygosity and allele frequencies [62, 63] and cannot capture true variants that are not present in reference haplotypes [64]. Full amelioration of reference bias likely requires a genome from all six species in the study, which are not currently available. If they do become available, the optimal strategy for reducing reference bias is not yet clear, but strategies like aligning the resequencing data to multiple assemblies, e.g., [65, 66] or to a *Vitis* pangenome may prove fruitful. Fortunately, however, at least two studies have evaluated reference bias in population genetics analyses, and they concluded that the effect of the reference bias is unlikely to bias broad demographic and evolutionary genomic analyses [62, 63].

### Evidence for extensive effects of introgression on genomes

Using the *V. arizonica* reference, we have produced a dataset of ~ 20 million SNPs from high coverage (~ 25×) data representing 130 accessions. We first used the SNPs to investigate genetic clustering and phylogenetic relationships. After the removal of 19 inferred hybrid individuals, we found that individuals from each species formed a monophyletic clade and also that inter-species relationships had strong support (Fig. 2). This last point is interesting because phylogenetic treatments of *Vitis* have not yet reached a broad consensus about species’ relationships [67]. The lack of phylogenetic resolution is not surprising given the rapid radiation and inter-fertility of *Vitis* species, but our results suggest that further phylogenetic analyses based on whole genome data and population-level sampling may clarify relationships within this model genus.

Despite the well-supported inter-species phylogeny, we have also detected introgression. *D* and *f* statistics detect introgression between six distinct pairs of species, among a total of eight tested pairs (Fig. 2C). These analyses also suggest that four of the six taxa (and most notably *V. riparia*) have complex histories of hybridization and introgression with potentially more than one species. The results also imply that 2 to 8% of the genomes of some species owe their origins to introgression (Fig. 2C and Table 1). For comparison, the lower value is similar to the 2% of the human genome inferred as having neanderthal origin [68], and the higher value exceeds the 5% percentage of genome introgressed between *Z. mays* ssp. *mays* and ssp. *mexicana* [69]. Overall, our work supports the idea that introgression shapes substantial regions of wild extant plant genomes.

It remains difficult, however, to infer the evolutionary forces that lead to the retention of introgressed regions within hybrid individuals [70]. One hypothesis is that they are retained because they reduce genetic load, particularly when deleterious alleles are recessive. Under this model, the introgressed region from a donor population contributes to lower load in a hybrid compared to non-introgressed members of the receptor population, thereby creating a fitness advantage. This scenario may be most likely for regions of the genome that (*i*) are unlikely to contribute numerous deleterious mutations (and hence likely to be gene poor); (*ii*) have high recombination rates, where interference among mutations is minimized [47]; and (*iii*) come from donor populations with higher effective population sizes (*N*_*e*_) than recipient species. This last point reflects the fact that high *N*_*e*_ species are generally expected to have a lower deleterious load. In our species, however, we do not find substantial variation in *N*_*e*_, because nucleotide diversity varies < 2-fold, making it hard to assess any relationship between population size and hybridization outcomes. Our results are, however, consistent with the first two points, because pIRs are gene poor in 5 of 9 trios (relative to randomly chosen genomic regions of the same size) and also because the pIRs in all trios are in regions with higher recombination rates than the genome-wide average (Table 1). Here we must recognize an important caveat: our trios are neither evolutionary nor statistically independent. As a consequence, conclusions based solely on the proportion of trios may be misleading. Nonetheless, these two specific trends are clear, because none of the nine trios have enriched gene density within the pIRs and because all nine trios have pIRs with higher recombination rates than the genomic background (Table 1).

### Potential adaptive significance of introgressed regions

Another non-exclusive hypothesis is that introgression is fueled by adaptation. Indeed, a recent simulation study has found that adaptation (and not deleterious load by itself) may be necessary to produce positive introgression statistics, particularly for regions that have high recombination rates and low gene density [71], like our pIRs. To investigate potential signals and causes of adaptation in our data, we have performed two distinct types of analyses. The first is selective sweep mapping within recipient species. While there were some compelling examples of overlapping sweeps and pIRs (Fig. 3), pIRs are generally not enriched for selective sweeps (Table 1). The lack of enrichment does not completely nullify an adaptive explanation for introgression, however, both because selective sweep mapping is inherently noisy and because sweeps are detectable only over a finite time frame. To the latter point, we believe that some pIRs reflect old events, based on two sources of information. First, most species pairs that show evidence for introgression do not currently grow in sympatry; SDMs suggest they were sympatric only in the past (Fig. 2D & Additional File 2: Figure S5). Second, some pIRs overlapped among trios, suggesting that introgression events pre-date the origin of current species (despite the lack of significant *f*-branch statistics on internal branches). For example, we compared two trios that had *V. riparia* as the P2 species. The first (GRM) had *V. monticola* as P3 and the second (GRB) had *V. berlandieri* as P3*.* The two trios had 142 and 293 pIRs, with 73 overlapping. This observation suggests the possibility that the introgression events contributing to these 73 pIRs pre-date the speciation of *V. monticola* and *V. berlandieri*.

Another approach to assess adaptation is to investigate associations with potentially adaptive functions. Accordingly, we have assessed gene content and explored associations with biotic and abiotic variables. For gene content, the pIRs have a consistent over-enrichment of genes involved in defense functions; seven of nine trios have pIRs that have more than the expected number of disease resistance genes, with five of seven statistically significant (Table 1). This phenomenon is most pronounced for *V. riparia*, because it is the P2 species in all five of these trios*.* Nonetheless, when we extend our work to include a quantitative phenotype—i.e., an assay of *X. fastidiosa* quantity after infection—we find that six of nine trios have pIRs enriched for SNPs associated with bacterial level, with three significantly so (Table 1). Interestingly, one of the three involves *V. arizonica*, so that our joint approach based on significant enrichments in gene content and bacterial levels implicates pIRs in disease function from a total of six of the nine trios (Table 1). We caution that pIR enrichment for associated SNPs is less marked when the GWAS model includes more latent factors (Additional File 2: Figure S18), and we must again recognize inherent limitations of our GWAS analyses and the lack of independence among trios. Nonetheless, the data on bacterial level and disease resistance enrichment (Fig. 4A) suggest that pathogen interactions could be a major feature shaping adaptive retention of introgressed regions in *Vitis*. Our results complement findings that plant-pathogen interactions play dominant roles in shaping the genetic diversity [72–74] and evolutionary dynamics [74–76] of plant populations, but they also extend these findings by suggesting that pathogen interactions shape the outcome of introgression events [77]. More empirical work is merited toward this end, both for *Vitis* species and plants more generally.

We have also assessed the potential for adaptive introgression by performing genome-environment associations, which show that climate-associated SNPs are highly significantly enriched within pIRs for at least one of the top three bioclimatic variables in eight of the nine trios (Table 1). One interesting pattern is that the results imply that cold-adapted alleles (e.g., as indicated by pIR associations with BIO6) have introgressed into *V. riparia*. This is puzzling, both because *V. riparia* is the most cold-hardy species among North America wild *Vitis* [78] and also because our sample represents a geographic region that is likely to experience warmer temperatures than most *V. riparia* populations. However, recent studies about the physiological processes involved in grape cold hardiness may provide clues to this puzzle [79, 80]. Perennial plants reduce seasonal cold hardiness by the process of deacclimation, and the rate of deacclimation varies among wild grape species [79]. Because deacclimation affects the time of budbreak, it is a critical process for reproduction. We speculate that historical introgressions into *V. riparia* may have facilitated adaptation to southern regions by contributing alleles that avoid premature budbreak during the midwinter temperature increases that can be common in the Southwest. Clearly, this speculation requires further study to be tested thoroughly.

There is, however, another intriguing possibility, which is that climate-associations do not directly reflect adaptation to climate but rather serve as indirect indicators of biotic stressors within a particular climate. Some evidence from this possibility comes from the observation that, on average, 17.8% of the genes annotated as plant resistance genes in pIRs had a candidate SNP associated with a bioclimatic variable. If true, this idea strengthens the hypothesis that the complex history of introgression among *Vitis* species is driven in part by adaptation to biotic stress, which parallels inferences based on introgression events between neanderthals and humans [81] and between *Populus* species [77]. Overall, these results suggest that evolution has promoted the exchange of resistance alleles and genes among species, which—somewhat paradoxically—is one of the major goals of modern rootstock breeders. Hence, the pIRs identified by this study may prove to be valuable regions for agronomic focus.

## Conclusions

In this study, we show that six wild *Vitis* species are genetically distinct, but there is widespread evidence of introgression among species, encompassing up to 8.0% of the genome. We also found that all species-pairs with evidence for introgression had some geographic overlap either in the present or in the past. The identified pIR were characterized by lower gene density and high recombination rates, matching theoretical expectations for introgressed regions. Phylogenetic analysis of the pIRs was also consistent with a history of introgression. Finally, we found some evidence that mutations significantly associated with pathogenic bacterial levels and bioclimatic variables are overrepresented in the pIRs, suggesting that these genomic regions have been exchanged and retained because of their adaptive benefits. The putative adaptive benefits suggest a model*—*in *Vitis* and perhaps more generally—for which biotic interactions are a major factor shaping the outcome of hybridization.

## Methods

### *V. arizonica* genome

#### Genome assembly

The *V. arizonica* genome was assembled using a hybrid strategy combining Single Molecule Real-Time (SMRT; Pacific Biosciences) data, which were reported previously [32], with the addition of Bionano NGM maps (Bionano Genomics). Genome sequences were assembled using SMRT reads following the custom procedure reported in https://github.com/andreaminio/FalconUnzip-DClab [82]. The pipeline performs the marking of repetitive content in SMRT reads using the TANmask and REPmask modules from DAmasker suite [83], both on raw reads before error correction and on the corrected reads used by FALCON ver. 2017.06.28-18.01 [84] to assemble the genome. Multiple combinations of assembly parameters were tested to reduce the sequence fragmentation. We used the following parameters for the final assembly:

$$ \mathrm{length}\_\mathrm{cutoff}\_\mathrm{pr}=7500 $$

$$ \mathrm{ovlp}\_\mathrm{DBsplit}\_\mathrm{option}=-\times 500 $$

$$ \mathrm{ovlp}\_\mathrm{HPCdaligner}\_\mathrm{option}=-\mathrm{mtan}-\mathrm{mrep}2-\mathrm{v}-\mathrm{B}128-\mathrm{M}60-\mathrm{t}60-\mathrm{k}20-\mathrm{h}256-\mathrm{e}.9-\mathrm{l}1000-\mathrm{s}100-\mathrm{T}16 $$

$$ \mathrm{overlap}\_\mathrm{filtering}\_\mathrm{setting}=--\max \_\mathrm{diff}\ 100--\max \_\operatorname{cov}\ 400--\min \_\operatorname{cov}\ 3 $$

FALCON_unzip was applied with default parameters to produce the primary contigs and the associated contigs [84] that were then polished from sequence error using PacBio reads with Arrow (from ConsensusCore2 ver. 3.0.0). Primary assembly contiguity was improved by scaffolding with SSPACE-Longread ver. 1.1 [85], followed by a gap-closing procedure with PBJelly (PBsuite ver. 15.8.4) [86, 87].

An optical map was based on Bionano molecules (Bionano Genomics) that were generated and assembled at the genome center of the University of California, Davis (Ming-Cheng Luo). Ultra-high molecular weight DNA (> 500 kbp) was extracted from young leaves by Amplicon Express (Pullman, WA). DNA was then labeled with a DLE-1 non-nicking enzyme (CTTAAG) and stained according to the Bionano Prep™ Direct Label and Stain (DLS) Kit (Bionano Genomics, San Diego, CA) instructions. Labeled DNA was loaded onto the SaphyrChip nanochannel array for imaging on the Saphyr system (Bionano Genomics, San Diego, CA). Optical maps were assembled with BioNano Solve ver. 3.3 [88]. The optical maps obtained were then used to scaffold the PacBio assembled sequences using HybridScaffold ver. 04122018 [88]. The procedure was performed in four iterations. In the first iteration, both sequences and optical maps were broken (“-B 2 -N 2”) when in conflict one with the other. In the second iteration, the scaffolds produced as results of the first iteration were compared to the optical maps, again both sequences and optical maps were broken (“-B 2 -N 2”) when in conflict. In the third iteration, scaffolds resulting from the previous step were compared to the optical maps, conflicts were resolved by breaking nucleotide sequences (“-B 1 -N 2”). In the fourth iteration, results of the previous scaffolding were used, conflicts were again resolved by breaking nucleotide sequences (“-B 1 -N 2”). The genomic sequences obtained were organized and sorted into two sets of chromosomes by using HaploSync ver. 0.1beta (https://github.com/andreaminio/HaploSync) and based on synteny with *Vitis vinifera* PN40024 chromosomes.

#### cDNA library preparation and sequencing

To help annotate the genome, we performed RNAseq using both short- and long-read sequencing technologies. Total RNA from *V. arizonica* leaves was isolated using a Cetyltrimethyl Ammonium Bromide (CTAB)-based extraction protocol as described in Ref. [89]. A Nanodrop 2000 spectrophotometer (Thermo Scientific, Hanover Park, IL) was then used to evaluate RNA purity. The RNA quantity was evaluated with the RNA broad range kit of the Qubit 2.0 Fluorometer (Life Technologies, Carlsbad, CA) and the integrity using electrophoresis and an Agilent 2100 Bioanalyzer (Agilent Technologies, CA). Total RNA (300 ng, RNA Integrity Number > 8.0) was used for cDNA synthesis and library construction. An RNA-Seq library was prepared using the Illumina TruSeq RNA sample preparation kit v.2 (Illumina, CA, USA) following Illumina’s Low-throughput protocol. This library was evaluated for quantity and quality with the High Sensitivity chip in an Agilent 2100 Bioanalyzer (Agilent Technologies, CA) and was sequenced in 100 bp single-end reads, using an Illumina HiSeq4000 sequencer (DNA Technology Core Facility, University of California, Davis). To prepare a cDNA long-read SMRTbell library, first-strand synthesis and cDNA amplification were accomplished using the NEBNext Single Cell/Low Input cDNA Synthesis & Amplification Module (New England, Ipswich, MA, USA). The obtained cDNAs were then purified with ProNex magnetic beads (Promega, WI) following the instructions in the Iso-Seq Express Template Preparation for Sequel and Sequel II Systems protocol (Pacific Biosciences, Menlo Park, CA). Amplified cDNA was size-selected with a mode of 2 kb using ProNex magnetic beads (86 μl). At least 80 ng of the size-selected, amplified cDNA was used to prepare the cDNA SMRTbell library using the SMRTbell Express Template Prep Kit 2.0 (Pacific Biosciences, Menlo Park, CA), following the manufacturer’s protocol. One SMRT cell was sequenced on the PacBio Sequel I platform (DNA Technology Core Facility, University of California, Davis). RNAseq data were submitted to NCBI under accession BioProject: PRJNA705722.

#### Genome annotation

The structural annotation of the *V. arizonica* genome was performed with the pipeline described here: https://github.com/andreaminio/AnnotationPipeline-EVM_based-DClab, an adaptation of a pipeline used previously [90] to use IsoSeq data as primary experimental evidence. In brief, high-quality Iso-Seq data from *V. arizonica* were used in PASA ver. 2.3.3 [91] to generate a set of high-quality gene models for the training of the following ab initio predictions software: Augustus ver. 3.0.3 [92], GeneMark ver. 3.47 [93], and SNAP ver. 2006-07-28 [94]. Gene predictions were generated also using BUSCO ver. 3.0.2 [95] with OrthoDB ver. 9 Plant conserved proteins. Repeats were annotated using RepeatMasker ver. open-4.0.6 [96] with the *Vitis* custom repeat library reported in Ref. [97].

Transcriptomic data was based on new data from *V. arizonica*, data from previous work [97], *Vitis* ESTs (NCBI, download date: 2016.03.15) and *Vitis* mRNA excluding transposable element-related proteins (NCBI, download date: 2016.03.15). RNAseq data were processed using Stringtie ver. 1.3.4d [98] and Trinity ver. 2.6.5 [99] with both on-genome and de novo protocols. Transcriptomic data was mapped, along with transcriptome assemblies and from the Iso-Seq data described above, on the *V. arizonica* genome using PASA ver. 2.3.3 [91] and MagicBLAST v.1.4.0 [100]. Protein evidence obtained from swissProt viridiplantae (download date: 2016.03.15) and Vitis proteins excluding transposable element-related proteins (NCBI, download date: 2016.03.15) were mapped on the genomic sequences using Exonerate ver. 2.2.0 [101]. Predictions and experimental evidence were then processed by EvidenceModeler ver. 1.1.1 [102] to generate consensus gene models and alternative splicing information integrated from the available transcriptomic data using PASA ver. 2.3.3 [91]. The final functional annotation was produced integrating into Blast2GO ver. 4.1.9 [103] hits from the blastp ver. 2.2.28 [104] results against the Refseq plant protein database (ftp://ftp.ncbi.nlm.nih.gov/refseq, retrieved January 17th, 2017) and InterProScan ver. 5.28-67.0 [105].

Disease-related gene functions were annotated using the HMM models from the Disease Resistance Analysis and Gene Orthology (DRAGO 2) database [49]. All the 28,259 predicted proteins from the primary reference chromosomes were evaluated in DRAGO2. Enrichment analysis of functional categories was tested using GeneMerge v1.4 [106].

### Genetic diversity data and analyses

#### Plant material

We collected fresh leaf tissue from 130 individuals from six American *Vitis* species in the wild grape germplasm collection at Davis, California. The germplasm reported in this study was collected from 1997 to 2016 as cuttings across the southwestern states and maintained at the Department of Viticulture and Enology, University of California, Davis, CA. Additional File 1: Table S3 provides details of global positioning coordinates and species designation based on the morphological features of leaves and growth habit of the field grown plants.

#### Genome resequencing

For the 130 samples, genomic DNA was extracted from leaf samples with the Qiagen DNeasy plant kit. The sequencing libraries were constructed with an insert size of ~ 300 bp using Illumina library preparation kits and were sequenced using the Illumina HiSeq 2500 platform with 2 × 150 bp paired reads to a target coverage of 25× following ref. [107]. The raw sequencing data has been deposited in the Short Read Archive at NCBI under BioProject ID: PRJNA731597.

#### SNP call and filtering

We filtered and evaluated raw reads using Trimmomatic-0.36 [108] and FastQC (http://www.bioinformatics.babraham.ac.uk/projects/fastqc/). Filtered reads were then mapped to the reference genome with the BWA-MEM algorithm [109] implemented in bwa-0.78 [110]. Joint SNP calling was conducted using the HaplotypeCaller in the GATK v.4.0 pipeline following ref. [107]. We then filtered raw SNPs with bcftools v1.9 (https://samtools.github.io/bcftools/) and vcftools v0.1.15 (https://vcftools.github.io/). We kept SNP sites for downstream analysis if they were biallelic, had quality higher than 30, had a depth of coverage higher than five reads, had no more than three times the median coverage depth across accessions, and also had less than 25% of missing data among all samples. Additionally, the following expression was applied under the exclusion argument of the filter function in bcftools: “QD < 2.0 | FS > 60.0 | MQ < 40.0 | MQRankSum < − 12.5 | ReadPosRankSum < − 8.0 | SOR > 3.0”.

#### Population structure and phylogenetic analyses

We used NgsAdmix to evaluate the genetic structure and the *thetastat* program to measure genetic diversity (*π*) within species from the ANGSD package v0.915 [33]. We tested values of *K* from 1 to 10 and used a minMaf filter of 0.05. We then employed the Cluster Markov Packager Across K (Clumpak) software [111] to detect the best *K* number of clusters*.* For the overall consensus phylogenetic tree, we used the SNPhylo pipeline (v20180901) to reduce the number of SNPs [112], using a linkage disequilibrium threshold of 0.5 and resulting in 15,893 informative sites. We then created a maximum likelihood phylogeny with the sites from SNPhylo using IQ-TREE v1.6.12 [113]. We used the Model Finder algorithm [114] implemented in IQ-TREE to search for the substitution model best among 550 different combinations and ultimately applied the “VT + F + R5” model. We used the ultrafast bootstrap option with 1000 replicates to obtain support values for each node. For the densitree (in red, Fig. 1C), we divided the genome in 10 kb windows but focused on used windows with at least 800 variable sites, resulting in 3342 windows. We created a consensus tree for each of the 3342 windows with 1000 replicates. We then focused on trees with a median bootstrap value higher than 70% across all nodes, reducing the number of windows to 1172, and randomly chose 500 of the 1172 trees for plotting. We used the R packages ape v5.4 [115] and phangorn v2.5.5 [116] to create the densitree plot and calculate tree statistics.

We also created a clock calibrated tree with BEAST v2.6.4 [117] using the 15,893 informative sites found by SNPphylo. BEAST was run in a chain length of 10 million with sampling every 500. A 10% burn-in was used to create the final tree (Additional File 2: Figure S3). To calibrate the time estimates, we used the estimates from ref. [22] of separation of *M. rotundifolia* with the *Vitis* subgenera as a prior, with a log normal distribution. For the time estimation, we used the Gamma Site Model distributions with the JTT site substitution model, a strict clock model, and the calibrated Yule model. We plotted multiple tree from BEAST using densitree v2.6.4 [118].

#### Admixture proportions

To test for introgression, we used the ABBA-BABA test as part of the Dsuite software [40]. First, we used the program Dtrios to calculate the overall *D* statistic and perform a block jackknifing of the statistic to obtain an associated *p* value, treating non-hybrid samples of each species as populations. The trios that were concordant with the 4-taxon topology of the test and had a *p* value < 0.001 were then used for more detailed analysis. For the *f*_*d*_ and *f*_dM_ tests of introgression, we used the program Dinvestigate from Dsuite, choosing non-overlaping windows of 1000 bp SNPs throughout the whole genome. We defined a pIR as windows with highest *x*% of *f*_dM_ values, where *x* was determined for each trio by the *f*_4_-ratio estimate (Table 1). To test for potential false positives of the *f*_4_-ratio estimates in closely related species we also calculated the f-branch statistic using Dsuite. We evaluated the phylogenies in the pIRs by creating phylogenetic trees of 10 kb SNP windows using the “phyml_sliding_windows.py” script and then using the “twisst.py” script to calculate average topology weights from the software twisst (https://github.com/simonhmartin/twisst) [46].

#### Selective sweeps

To detect selective sweeps, we used RAiSD v2.8 [119] for each species separately, with default parameters. We removed the gaps of the reference genome from the analysis to avoid potential errors. We split the genome into non-overlapping 10 kb windows created by bedtools v2.27.1 [120] and focused on the windows with top 5% of the *μ* statistic in the corresponding P2 population and defined those as highly supported sweeps. We first calculated the number of top 5% *μ* windows from the P2 species overlapping the IRs of each trio, using bedtools with a requirement of at least 50% of the window overlapping with the IR (-f 0.5). For the enrichment analysis, we randomly chose the same number of 10 kb windows as the number of HSSs chosen from the whole genome with 10,000 replicates. We counted the number of the random windows overlapping IRs in the same way as described before. We then compared the distribution of the observed values with the randomly generated distribution and performed a *t*-test to evaluate statistical significance. For plotting, we calculated average *μ* values for a 100 kb window using the bedtools mapping function.

#### Recombination

To estimate the location of pIRs relative to recombination rates across the genome, we employed the genetic mapping data from a previous study [48]. Briefly, the map data defined the recombination rate between two markers, based on a consensus of four different mapping populations that used both wild and cultivated *Vitis* parents. The physical location of 1662 markers was provided on the PN40024 *V. vinifera* reference. Since the *V. arizonica* assembly was anchored on the PN40024 *V. vinifera* reference, we were able to transform the markers and recombination values [48] and then assign recombination values (in cM/kb) to discrete regions of the *V. arizonica* genome.

### Phenotype data, climate data, and SNP associations

#### Pierce’s disease assays

Evaluations for Pierce’s disease (PD) resistance were carried out using a greenhouse-based screen [121, 122]. Accessions with strong and intermediate PD resistance and inoculated and un-inoculated susceptible *V. vinifera* cultivar Chardonnay were used in all experiments as reference control plants. Nineteen screens were carried out from 2011 to 2020, and a minimum of four biological replicates of each accession were tested. Disease severity was accessed 10 to 14 weeks post inoculation and ELISA was used to measure the *X. fastidiosa* levels in the stem. Statistical analysis was performed using JMP Pro14 software (Copyright 2020, SAS Institute Inc.) to determine the variability of ELISA for the reference control plants across 19 experiments. In the next step, ELISA values of wild accessions were analyzed with the inclusion of the reference plants to adjust for variation among the 19 screens. PD resistance data for 38 of the 108 accessions used in this study were from a previous study [122] (Additional File 1: Table S10).

#### Genome-wide association studies (GWAS)

We used LFFM2 to evaluate the association between SNPs and *X. fastidiosa* bacterial level [123]. To control for demographic structure in the across-species associations, we used 7 latent factors (LF) based on the genetic clusters inferred by Admixture and PCA analyses (Additional File 2: Figure S15). We first used the function *lfmm_ridge* and *K* = 7 to compute the regularized least squares using a ridge penalty. Next, we used the *lfmm_test* function to estimate the genomic inflation factor based on the median *Z* scores. We verified that the calibrated *p* values had a flat distribution with a slight peak near zero (Additional File 2: Figure S15B-C). Since genetic structure could increase the number of false positives, we also compared GWAS models based on a linear regression model that does not control for genetic structure (see [51, 124, 125]), and models using LFMM with LF values ranging from *K* = 6 to *K* = 12 (Additional File 2: Figure S18).

To avoid biases associated to imputation (LFMM needs non-missing data), all GWAS models were applied to the subset of 397,723 SNPs that had no missing data across the dataset of *n* = 108 accessions. We also used an FDR of 0.05 to detect candidate SNPs and applied GWAS to data with and without a minor allele frequency of < 5%, with no change in results. Once candidate SNPs were identified, we tested for enrichments of SNP density within pIRs. To do so, we randomized the state (candidate-associated SNP or non-candidate SNP) of observed SNPs across the genome. For each randomization, we counted the total number (*t*_*i*_) of randomly assigned candidate SNPs within pIRs. We performed 10,000 randomizations and compared the distribution of *t*_*i*_ to the observed value (*t*_*obs*_) to compute a *p* value. Note that this approach inherently accounts for any differences in the density of SNPs across chromosomal regions.

#### Genome environment associations (GEA)

Genome environment association studies identify outlier loci that correlate with the environment while controlling for demographic structure [126]. We used BayPass version 2 [52] to identify outlier loci that correlated with 19 bioclimatic variables. BayPass first uses the entire set of SNPs to estimate the covariance between populations and identify populations that are genetically closer because of recent co-ancestry or lower genetic structure. In a second step, BayPass analyzes the correlation between the allelic frequency of each SNPs (response variable) and independent environmental, phenotypic, and/or categorical variables. SNPs are considered candidates if they show a strong correlation with the independent variable after controlling for the co-ancestry between populations.

We obtained 19 bioclimatic variables from Worldclim [127] for the location of each accession, using the *extract* function of the raster package [128] in R (R Core Team). For each species, we ran BayPass using all the default parameters, using all polymorphic SNPs without any missing data (Nloc_*arizonica*_ *=* 5,456,474 SNPs; Nloc_*berlandieri*_ *=* 3,859,330 SNPs; Nloc_*candicans*_ *=* 4,971,805 SNPs; Nloc_*girdiana*_ *=* 3,598,951; Nloc_*monticola*_ *=* 4,664,260 SNPs; Nloc_*riparia*_ *=* 5,630,334 SNPs) and the 19 bioclimatic variables. Outlier SNPs were defined using Jeffreys’ rule [53], so that they were considered outliers if they had a BF > 10. For each species, we identified three bioclimatic variables that showed the highest number of outlier loci and focused on outlier SNPs. We further analyzed the set of outlier SNPs to see if they were enriched in pIRs. We used bedtools v2.27.1 [120] to obtain the overlap between the candidate SNPs of the top 3 bioclimatic variables of each species and the pIRs. For each trio showing evidence of introgression, we used the randomization approach described above for PD associations to test whether pIRs in the P2 species were enriched with outlier SNPs associated with a bioclimatic variable.

#### Species distribution models

We constructed species distribution models (SDMs) to identify whether species having evidence of introgression had overlapping distributions in the present, the Holocene (~ 6000 ya) and the Pleistocene (~ 18,000 ya). For each species, we obtained occurrence records by combining coordinates data from the Global Biodiversity Information Facility (GBIF.org, 7 August 2020). Most of the six species analyzed had adequate occurrence data (> 70 entries), except *V. monticola* with 20 entries. The low values for *V. monticola* are likely to lead to an overestimation of its geographic distribution. We also obtained 19 bioclimatic variables from WorldClim [127] at a 2.5 resolution for the present, Holocene, and Pleistocene layers. For the past layers, we obtained bioclimatic data projected with the general circulation model CCSM [127].

We employed Maxent V. 3.4.1 [129] to construct and train the SDM of each species and then projected them onto the landscape for each time period. For each species, we ran 30 bootstrap replicates and used 70% and 30% of data for training and validating the models, respectively. Additionally, we evaluated the fit of the models by analyzing the area under the curve (AUC) of the receiver-operating characteristic curve (ROC). For each species, we binarized each replicate by defining as the probability of existence all areas that had a probability value in which the omission rate of the training and testing was above the 10% of prevalence, and/or that predicted the accessions we sampled. For each species, we summed all the binarized models and defined as the final distribution all geographic areas that were predicted by > 60% of the bootstrap replicates.

We used SDM to predict areas of overlap between pairs of species at different time periods. To simplify the data, for each period, first, we summed the SDM of each pair of 15 species pairs retained areas that were predicted by both species. The present, Holocene, and Pleistocene overlapped regions were arbitrarily set at values of 2, 5, and 10. Second, we summed the three time periods to identify the areas where each pair of species were potentially sympatric at different periods. Areas with values 2, 5, and 10 correspond to areas where there have been overlaps only in the present, Holocene, or Pleistocene, respectively. Areas with values 7, 12, or 15 are areas where there have been overlaps in the present and Holocene, present and Pleistocene, or Holocene and Pleistocene, respectively. Areas with value 17 are areas where there has been a continuous overlap between the pair of species. For each pair of species, we obtained the geographic area (measured by the number of overlapping pixels) that were predicted to have an overlap in the SDMs in the different periods.

## Supplementary Information

**Additional file 1: Table S1.** Statistics of the *V. arizonica* b40-14 genome assembly. **Table S2.** Summary table of the BUSCO genes of both haplotypes detected in the *V. arizonica* genome assembly. **Table S3.** Accession identifiers and geographical coordinates of the 130 accessions used in this study. **Table S4.** Chromosome size, raw number of predicted SNPs and number of SNPs after filtering across all 130 samples. **Table S5.** Genome-wide calculation of nucleotide diversity (휋) within per species. **Table S6.** Results from genome-wide estimates of introgressions across all combination of trios tested. **Table S7.** GO terms significantly enriched (p < 0.05) in pIRs. **Table S8.** Gene density averages per 10 kb and relative enrichment. **Table S9.** Recombination rates per trio and values relative to the genome-wide estimated rate of recombination. **Table S10.** Quantitative measurements of Pierce’s Disease evaluations. **Table S11.** Enrichments of SNPs significantly associated with PD in pIRs. **Table S12.** Enrichments of SNPs associated to the top three bioclimatic variables per trio in pIRs.

**Additional file 2: Figure S1.** Genetic clustering of all samples (including hybrid samples) detected by the structure analyses from K = 2 to K = 10. **Figure S2.** Consensus maximum likelihood phylogenetic tree created with IQtree using 15,893 informative sites found by SNPphylo. **Figure S3.** Clock calibrated phylogenetic tree created with BEAST using 15,893 informative sites found by SNPphylo. **Figure S4.** Results of the Fbranch statistic using species tree models. **Figure S5.** Pairs of species that show evidence of introgression present evidence of overlap at three periods: present, Holocene, Pleistocene. **Figure S6.** Pairs of species that do not show evidence of introgression present no or low overlap at three periods: present, Holocene, Pleistocene. **Figure S7.** Pairs of species that do not show evidence of introgression but have overlapping distributions at three periods: present, Holocene, Pleistocene. **Figure S8.** Genomic size distribution of the windows used to calculate the ABBA-BABA statistics per trio. **Figure S9.** Distribution the *f*_dM_ statistic values across windows per trio. **Figure S10.** Distribution the f_d_ statistic values across windows per trio. **Figure S11.** Manhattan plot of all the middle point value all windows with fdM values for all nine trios. **Figure S12.** Scatterplot showing correlations between the f_dM_ and f_d_ statistics across all windows in the genome. **Figure S13.** Mean gene density of across the whole genome and within the pIRs of all the trios. **Figure S14.** Average weights of phylogenetic relationships in the pIRs. SNPs from pIRs for each trio were split in 10Kb windows to create a phylogenetic tree for each window. **Figure S15.** Principal component variance LFMM association analyses. **Figure S16.** QQ plots showing the logarithm of the p-values for a linear regression model, the LFMM model using 7 LF and without controlling for a genome inflation factor and controlling for a genome inflation factor. **Figure S17.** Manhattan plot of LFMM association results for the concentration of *X. fastidiosa*. The red lines indicate the threshold at an FDR < 0.05. **Figure S18.** QQ plots showing the logarithm of the p-values for a linear regression model (gray), and LFMM models using 6-12 LF and controlling for a genome inflation factor. **Figure S19.** Number of associated candidate SNPs per bioclimatic variables per species.

**Additional file 3.** Review history.

**Additional file 4: Dataset S1.** Genomic windows identified as putative introgressed regions (pIRs) across nine trios.

**Additional file 5: Dataset S2.** Gene functional annotation for the genome reference of *Vitis arizonica* b40-14 version 1.

**Additional file 6: Dataset S3.** Significant SNPs (FDR adjusted p < 0.05) from GWAS with bacterial levels of the causative agent of Pierce’s Disease.

**Additional file 7: Dataset S4.** Significant SNPs (Bayes’ Factor > 10) from GEA with the top three bioclimatic variables per species.

**Additional file 8: Dataset S5.** Subset of significant SNPs (Bayes’ Factor > 10) from GEA with the top three bioclimatic variables in the P2 species that were in putative introgressed regions (pIRs).

## Data Availability

Raw genome resequencing data of the 130 wild grape accessions are available at NCBI under BioProject ID PRJNA731597 [130]. Transcriptome sequencing data of *V. arizonica* b40-14 used for gene model predictions are available at NCBI under BioProject ID: PRJNA705722 [131]. The published [32] long-read *V. arizonica* sequencing data were taken from NCBI BioProject ID PRJNA593045. Genome assembly files and gene annotation files are available in Zenodo [132]. Source code for the genome assembly is available in Github [133]. The genome files and a genome browser can also be found at www.grapegenomics.com/pages/Vari/. The Datasets S1-S5 generated during this study are also available at Figshare [45].
