# Supplementary material for: Introgression among North American wild grapes (Vitis) fuels biotic and abiotic adaptation
Source: Genome Biol. 2021 Sep 3;22:254. doi: 10.1186/s13059-021-02467-z (PMC8414701; doi:10.1186/s13059-021-02467-z)
Supplement: Supplementary file 1 — Additional file 1: Table S1. Statistics of the V. arizonica b40-14 genome assembly. Table S2. Summary table of the BUSCO genes of both haplotypes detected in the V. arizonica genome assembly. Table S3. Accession identifiers and geographical coordinates of the 130 accessions used in this study. Table S4. Chromosome size, raw number of predicted SNPs and number of SNPs after filtering across all 130 samples. Table S5. Genome-wide calculation of nucleotide diversity (휋) within per species. Table S6. Results from genome-wide estimates of introgressions across all combination of trios tested. Table S7. GO terms significantly enriched (p < 0.05) in pIRs. Table S8. Gene density averages per 10 kb and relative enrichment. Table S9. Recombination rates per trio and values relative to the genome-wide estimated rate of recombination. Table S10. Quantitative measurements of Pierce’s Disease evaluations. Table S11. Enrichments of SNPs significantly associated with PD in pIRs. Table S12. Enrichments of SNPs associated to the top three bioclimatic variables per trio in pIRs. [file 13059_2021_2467_MOESM1_ESM.pdf]

Additional File 1: Supplementary Tables S1-S12 for

Extensive introgression among North American wild grapes (*Vitis*) fuels biotic and abiotic adaptation

Abraham Morales-Cruz[1], Jonas Aguirre-Ligouri[1], Yongfeng Zhou[1], Andrea Minio[2], Summaira Riaz[2], Andrew M. Walker[2], Dario Cantu[2], and Brandon S. Gaut*[2]

[1] Dept. Ecology and Evolutionary Biology, University of California Irvine, Irvine, CA

[2] Dept. of Viticulture, University of California, Davis, Davis, CA

*Address for Correspondence: Brandon Gaut, Department of Ecology and Evolutionary Biology, 321 Steinhaus Hall, UC Irvine, 92697; Email: bgaut@uci.edu; Phone: (949) 677-06972

Table S1. Statistics of the *V. arizonica* b40-14 genome assembly

Metric	Number
Total number of bases	503291318
Number of sequences	19
Average length of sequences	2.65E+07
Minimum length of sequences	17755125
Maximum length of sequences	47005314
N10 length	35285526
N20 length	33926803
N30 length	30932466
N40 length	30043575
N50 length	25930761
N60 length	25085101
N70 length	23233325
N80 length	22502345
N90 length	19267294
N10 index	2
N20 index	3
N30 index	5
N40 index	6
N50 index	8
N60 index	10
N70 index	12
N80 index	14
N90 index	17
Number of sequences >= 100bp	19
Avg length of sequences >= 100bp	2.65E+07
N50 sequences >= 100bp	25930761

Table S2. Summary table of the BUSCO genes of both haplotypes detected in the *V. arizonica* genome assembly

Metric	Count	Percentage
Complete BUSCOs (C)	1389	96.40%
Complete and single-copy BUSCOs (S)	493	34.20%
Complete and duplicated BUSCOs (D)	896	62.20%
Fragmented BUSCOs (F)	14	1.00%
Missing BUSCOs (M)	37	2.60%
Total BUSCO groups searched	1440	

Table S3. Accession identifiers and geographical coordinates of the 130 accessions used in this study. Coordinates correspond to the original collection site of the accession.

Accession	Sample ID	Species after phylogeny	Latitude	Longitude
NM11-048	vari12	*V. arizonica*	33.03475	-108.20006
NM11-031	vari11	*V. arizonica*	32.91086	-108.22894
NM11-047	vari21	*V. arizonica*	33.03644	-108.20619
NM11-040	vari16	*V. arizonica*	33.03514	-108.22894
NM11-034	vari14	*V. arizonica*	32.91928	-108.21964
NM11-033	vari13	*V. arizonica*	32.91944	-108.21933
NM11-046	vari07	*V. arizonica*	33.03928	-108.22067
NM11-035	vari03	*V. arizonica*	32.92264	-108.21567
NM11-026	vari09	*V. arizonica*	32.89156	-108.23436
TXNM088	vber09	*V. arizonica*	29.28985	-103.92507
NM11-044f	vari20	*V. arizonica*	33.03994	-108.22264
NM11-044a	vari19	*V. arizonica*	33.03994	-108.22264
NM11-042	vari17	*V. arizonica*	33.03503	-108.22875
NM11-038	vari15	*V. arizonica*	32.94819	-108.20100
NM11-027	vari10	*V. arizonica*	32.90675	-108.23219
NM11-039	vari06	*V. arizonica*	32.94953	-108.20667
ANU58	vrip15	*V. arizonica*	34.51501	-111.76801
NM11-043	vari18	*V. arizonica*	33.03994	-108.22264
NM11-037	vari05	*V. arizonica*	32.93492	-108.20075
NM11-036	vari04	*V. arizonica*	32.92881	-108.21033
NM11-045	vari02	*V. arizonica*	33.03961	-108.22000
NM11-049	vari01	*V. arizonica*	33.03500	-108.20050
TX16-022	vber21	*V. berlandieri*	30.84407	-98.09657
TX16-032	vber14	*V. berlandieri*	30.43791	-98.34980
TX16-064	vcan26	*V. berlandieri*	30.24638	-98.04376
TX16-018	vber18	*V. berlandieri*	31.03509	-98.14784
TX16-065	vber28	*V. berlandieri*	30.14990	-98.05138
TX16-026	vber12	*V. berlandieri*	30.79163	-98.16845
TX15-003	vber10	*V. berlandieri*	29.84893	-100.02290
TX16-034	vber26	*V. berlandieri*	30.03024	-98.83222
TX16-035	vber27	*V. berlandieri*	30.02967	-98.83170
TX16-016	vber17	*V. berlandieri*	31.33896	-97.51378
TX15-059	vber11	*V. berlandieri*	30.43829	-98.34963
T21	vber02	*V. berlandieri*	30.48940	-99.77200

T18	vber29	*V. berlandieri*	30.48940	-99.77200
TX15-063	vber23	*V. berlandieri*	30.68166	-98.31197
C 8-93	vber20	*V. berlandieri*	31.67678	-98.04191
C 5-93	vber19	*V. berlandieri*	31.16966	-97.42910
TX16-030	vber13	*V. berlandieri*	30.68943	-98.27036
T 03-05 S03	vber07	*V. berlandieri*	30.24687	-98.87010
TX9722	vber05	*V. berlandieri*	28.70910	-100.04950
T38	vber04	*V. berlandieri*	30.40020	-97.67910
T23	vber03	*V. berlandieri*	30.04740	-99.14030
TX43-01	vber01	*V. berlandieri*	30.31996	-97.99865
T45	vcan02	*V. candicans*	30.75930	-98.67500
T64	vcan17	*V. candicans*	31.13000	-97.78000
TX9715	vcan15	*V. candicans*	29.34750	-99.14140
candicans 9003	vcan29	*V. candicans*	32.49250	-95.80917
candicans 9005	vcan30	*V. candicans*	32.49250	-95.80917
T62	vcan16	*V. candicans*	31.27230	-97.51570
T36	vcan13	*V. candicans*	30.40020	-97.67570
TX12-003	vcan09	*V. candicans*	33.39661	-97.50039
TX32-01	vcan04	*V. candicans*	30.04743	-99.14032
T48	vcan03	*V. candicans*	30.75620	-98.70030
T46	vcan14	*V. candicans*	30.75930	-98.67500
T2	vcan10	*V. candicans*	33.25760	-97.58324
T56	vcan08	*V. candicans*	31.09820	-97.34280
candicans 9039	vcan31	*V. candicans*	29.91972	-97.19306
candicans 9001	vcan28	*V. candicans*	31.76583	-95.61667
TX16-024	vcan27	*V. candicans*	30.81022	-98.10594
TX16-006	vcan25	*V. candicans*	31.10614	-97.91810
TX16-001	vcan24	*V. candicans*	30.93038	-97.52800
TX14-081	vcan23	*V. candicans*	30.00979	-97.94384
T 03-13	vcan21	*V. candicans*	30.83118	-99.39540
T 03-08	vcan20	*V. candicans*	30.16750	-98.77183
T 03-05	vcan19	*V. candicans*	30.24687	-98.87010
TX9703	vcan12	*V. candicans*	29.96270	-98.78630
T73	vcan11	*V. candicans*	31.38660	-97.15040
SC37	vgir19	*V. girdiana*	33.33007	-117.23638
NV12-040	vgir01	*V. girdiana*	36.16128	-115.49850
SC11	vgir25	*V. girdiana*	36.41840	-116.31895
SC9	vgir06	*V. girdiana*	36.41840	-116.31895
SC26	vgir23	*V. girdiana*	34.93163	-118.92795
SC39	vgir26	*V. girdiana*	33.31813	-117.19465

NV12-041	vgir14	*V. girdiana*	36.15642	-115.49542
NV11-118	vgir12	*V. girdiana*	36.37539	-114.44308
NV11-115	vgir10	*V. girdiana*	36.38999	-114.42996
ANU78	vgir02	*V. girdiana*	36.38925	-114.42955
NV12-050	vgir15	*V. girdiana*	36.41986	-116.32050
NV11-116	vgir11	*V. girdiana*	36.37678	-114.44361
SC53	vgir17	*V. girdiana*	33.10527	-117.04198
SC36	vgir27	*V. girdiana*	33.33007	-117.23638
SC30	vgir22	*V. girdiana*	33.46235	-117.13700
SC33	vgir21	*V. girdiana*	33.39145	-117.21640
SC40	vgir20	*V. girdiana*	33.36293	-117.10158
SC51	vgir16	*V. girdiana*	33.06660	-116.87915
C20-93-A	vmon05	*V. monticola*	31.06623	-97.57890
T 03-02 S01	vmon04	*V. monticola*	29.98305	-98.90348
monticola 9040	vmon07	*V. monticola*	32.49250	-95.80917
C20-93	vmon06	*V. monticola*	31.06623	-97.57890
T40	vmon02	*V. monticola*	30.30610	-97.95240
TX67-03	vmon01	*V. monticola*	31.43516	-97.74391
NM12-119	vrip21	*V. riparia*	36.21283	-105.92722
NM12-114	vrip07	*V. riparia*	35.98822	-105.93197
NM12-108	vrip19	*V. riparia*	35.32289	-105.41953
NM12-117	vrip09	*V. riparia*	36.15992	-105.97272
NM12-116	vrip08	*V. riparia*	35.98911	-105.93036
KS14-036	vrip12	*V. riparia*	37.24091	-99.98147
CO12-103	vrip02	*V. riparia*	37.11083	-104.28486
KS14-043	vrip23	*V. riparia*	37.29012	-98.61109
NM12-104	vrip18	*V. riparia*	35.66897	-105.34619
TXNM0824	vrip17	*V. riparia*	35.66893	-105.33610
TXNM0823	vrip16	*V. riparia*	35.66893	-105.33610
CO12-102	vrip14	*V. riparia*	37.21572	-104.46469
OK14-064	vrip13	*V. riparia*	36.53865	-98.87695
KS14-035	vrip11	*V. riparia*	37.24038	-99.98163
OK14-027	vrip10	*V. riparia*	36.83688	-100.51925
NM12-111	vrip06	*V. riparia*	35.26753	-105.33447
V28-96	vrip05	*V. riparia*	41.14871	-73.27039
V23-98	vrip04	*V. riparia*	41.71946	-72.57670
OK14-026	vrip01	*V. riparia*	36.83703	-100.51897
T39	vcan06	hybrid	30.30610	-97.95240
UT12-094	vgir05	hybrid	37.29369	-113.41414
OK12-025	vcan22	hybrid	34.10983	-98.53114

OK12-027	vcan07	hybrid	34.11075	-98.53250
OK12-019	vcan05	hybrid	34.12075	-98.52269
T42	vcan01	hybrid	30.32000	-97.99860
TXNM083	vber08	hybrid	30.53358	-103.78433
SC12	vgir09	hybrid	37.03228	-117.32460
NV11-119 (GVC)	vgir13	hybrid	35.22736	-114.68964
PC96-C	vrip22	hybrid	NA	NA
TX9714	vmon03	hybrid	29.34750	-99.14140
SC23	vgir24	hybrid	35.66705	-118.25247
SC27	vgir08	hybrid	34.93163	-118.92795
SC42	vgir07	hybrid	33.28781	-116.87448
UT12-084	vgir04	hybrid	37.30861	-113.42914
UT12-075	vgir03	hybrid	37.31033	-113.43553
candicans x aestivalis 9012	vcan32	hybrid	31.76583	-95.61667
T74	vcan18	hybrid	31.38660	-97.15040
T 03-01 S01	vber06	hybrid	29.98305	-98.90348

Table S4. Chromosome size, raw number of predicted SNPs and number of SNPs after filtering across all 130 samples

Chromosome	Size (bp)	Size (Mbp)	SNPs raw bialellic	SNPs after filtering
Vari_b40-14_v1.hap1.chr01	33,926,803	33.9	2,782,419	1,375,689
Vari_b40-14_v1.hap1.chr02	22,787,875	22.8	1,955,943	920,414
Vari_b40-14_v1.hap1.chr03	17,755,125	17.8	1,542,440	767,980
Vari_b40-14_v1.hap1.chr04	30,932,466	30.9	2,566,482	1,169,772
Vari_b40-14_v1.hap1.chr05	25,930,761	25.9	2,311,363	1,188,958
Vari_b40-14_v1.hap1.chr06	23,233,325	23.2	1,759,313	968,551
Vari_b40-14_v1.hap1.chr07	35,285,526	35.3	2,852,943	1,378,311
Vari_b40-14_v1.hap1.chr08	19,150,580	19.2	1,496,299	612,688
Vari_b40-14_v1.hap1.chr09	25,085,101	25.1	2,036,641	931,494
Vari_b40-14_v1.hap1.chr10	23,682,995	23.7	2,032,387	1,089,722
Vari_b40-14_v1.hap1.chr11	19,267,294	19.3	1,355,229	665,031
Vari_b40-14_v1.hap1.chr12	32,374,331	32.4	2,849,773	1,316,433
Vari_b40-14_v1.hap1.chr13	30,043,575	30.0	2,576,413	1,324,160
Vari_b40-14_v1.hap1.chr14	27,053,463	27.1	2,430,724	1,229,304
Vari_b40-14_v1.hap1.chr15	22,502,345	22.5	1,999,980	836,821
Vari_b40-14_v1.hap1.chr16	25,629,229	25.6	2,132,614	916,236
Vari_b40-14_v1.hap1.chr17	19,376,636	19.4	1,776,932	918,695
Vari_b40-14_v1.hap1.chr18	47,005,314	47.0	3,832,273	1,639,733
Vari_b40-14_v1.hap1.chr19	22,268,574	22.3	1,807,138	741,236
**Total**	503,291,318	503	42,097,306	19,991,228

Table S5. Genome-wide calculation of nucleotide diversity (π) within per species

Species	π
V. monticola	0.00353
V. riparia	0.00312
V. candicans	0.00304
V. arizonica	0.00270
V. girdiana	0.00255
V. berlandieri	0.00211

Table S6. Results from genome-wide estimates of introgressions across all combination of trios tested.

Trio	P1	P2	P3	D	Z-score	p-value	f4-ratio	BBAA	ABBA	BABA
GAM	*girdiana*	*arizonica*	*monticola*	0.086	15.764	0.00E+00	8.03%	312836	147982	124492
GRM	*girdiana*	*riparia*	*monticola*	0.062	10.308	0.00E+00	6.08%	294631	151684	133908
ARC	*arizonica*	*riparia*	*candicans*	0.091	21.944	0.00E+00	2.43%	411536	126061	105037
MBC	*monticola*	*berlandieri*	*candicans*	0.071	9.224	0.00E+00	3.24%	231272	189653	164656
GRC	*girdiana*	*riparia*	*candicans*	0.087	17.202	0.00E+00	2.30%	417236	124661	104798
ARB	*arizonica*	*riparia*	*berlandieri*	0.116	27.752	0.00E+00	7.40%	350794	140344	111062
GRB	*girdiana*	*riparia*	*berlandieri*	0.119	23.024	0.00E+00	7.47%	357161	139597	110007
AGR	*arizonica*	*girdiana*	*riparia*	0.021	2.961	1.54E-03	3.32%	204334	176756	169492
RAM	*riparia*	*arizonica*	*monticola*	0.019	2.737	3.10E-03	2.07%	281783	151658	145974
AGC	*arizonica*	*girdiana*	*candicans*	0.005	1.256	1.05E-01	0.13%	446358	106248	105140
GAB	*girdiana*	*arizonica*	*berlandieri*	0.001	0.358	3.60E-01	0.09%	388298	113818	113480

Table S7. GO terms significantly enriched ($p < 0.05$) in pIRs

Trio	GO ID	GO Description	p-value
AGR	GO:0005575	C:cellular_component	8.87E-04
ARB	GO:0005575	C:cellular_component	5.46E-03
ARB	GO:0030246	F:carbohydrate binding	1.81E-09
GRB	GO:0030246	F:carbohydrate binding	9.54E-06
GRC	GO:0030246	F:carbohydrate binding	5.34E-05
GRM	GO:0030246	F:carbohydrate binding	5.12E-25
ARB	GO:0016301	F:kinase activity	7.01E-07
GRB	GO:0016301	F:kinase activity	3.82E-02
GRM	GO:0016301	F:kinase activity	4.99E-12
AGR	GO:0008289	F:lipid binding	9.83E-03
AGR	GO:0003674	F:molecular_function	9.58E-03
ARB	GO:0003674	F:molecular_function	4.59E-02
ARB	GO:0000166	F:nucleotide binding	6.68E-07
GRB	GO:0000166	F:nucleotide binding	2.48E-05
GRC	GO:0000166	F:nucleotide binding	1.57E-02
GRM	GO:0000166	F:nucleotide binding	1.07E-10
ARC	GO:0004872	F:receptor activity	4.37E-02
ARC	GO:0038023	F:signaling receptor activity	1.55E-02
GRM	GO:0007154	P:cell communication	6.06E-04
ARC	GO:0007267	P:cell-cell signaling	2.21E-07
GRM	GO:0007267	P:cell-cell signaling	1.03E-03
GRM	GO:0006464	P:cellular protein modification process	1.45E-05
RAM	GO:0009790	P:embryo development	3.43E-02
RAM	GO:0009908	P:flower development	6.48E-04
GAM	GO:0006139	P:nucleobase-containing compound metabolic process	4.81E-02
GRM	GO:0009856	P:pollination	5.40E-05

Table S8. Gene density averages per 10 kb and relative enrichment as presented in Table 1.

Trio	Avg. gene density	Relative. Enrichment
Genome-wide	0.5762719	-
ARC	0.4417671	0.77
GRC	0.4383202	0.76
GRB	0.433835	0.75
ARB	0.4343598	0.75
GAM	0.5551546	0.96
RAM	0.5276498	0.92
GRM	0.4581006	0.79
MBC	0.4706927	0.82
AGR	0.5678322	0.99

Table S9. Recombination rates per trio and values relative to the genome-wide estimated rate of recombination as presented in Table 1.

Trio	Avg. cM/Kb	Relative to genome-wide
Genome-wide	0.0092	-
AGR	0.0148	1.61
ARB	0.0166	1.80
ARC	0.0113	1.23
GAM	0.0208	2.27
GRB	0.0132	1.43
GRC	0.0134	1.45
GRM	0.0111	1.21
MBC	0.0139	1.52
RAM	0.0101	1.10

Table S10. Quantitative measurements of Pierce's Disease evaluations.

Accession	Sample ID	Species	Category	Least Sq Mean	Std Error	Mean	Study[1]
NM11-036	vari04	vari	R	7.708	0.953	7.626	Riaz et al 2020
NM11-044f	vari20	vari	R	7.890	0.953	7.808	Riaz et al 2020
NM11-031	vari11	vari	R	7.942	0.948	7.376	This study
NM11-048	vari12	vari	R	8.604	1.056	8.0375	This study
NM11-026	vari09	vari	R	8.774	0.966	9.424	This study
NM11-038	vari15	vari	R	8.845	1.067	9.5825	This study
NM11-044a	vari19	vari	R	9.236	0.966	9.886	This study
NM11-043	vari18	vari	R	9.466	0.966	10.116	This study
NM11-049	vari01	vari	R	9.554	0.953	9.472	Riaz et al 2020
NM11-037	vari05	vari	R	10.211	0.669	9.354	This study
TXNM088	vber09	vari	R	10.724	0.953	10.642	Riaz et al 2020
NM11-033	vari13	vari	R	10.742	0.966	11.392	This study
NM11-027	vari10	vari	R	10.760	0.966	11.41	This study
NM11-046	vari07	vari	R	10.871	0.939	10.014	This study
NM11-035	vari03	vari	R	10.931	1.058	9.8675	This study
NM11-045	vari02	vari	R	11.182	0.953	11.1	Riaz et al 2020
NM11-042	vari17	vari	R	11.350	0.966	12	This study
NM11-040	vari16	vari	R	11.458	0.966	12.108	This study
NM11-047	vari21	vari	R	11.535	1.072	12.185	This study
NM11-039	vari06	vari	R	12.228	0.953	12.146	Riaz et al 2020
ANU58	vrip15	vari	R	12.485	0.939	11.628	This study
NM11-034	vari14	vari	R	12.762	0.948	12.196	This study
T18	vber29	vber	R	11.808	0.953	11.726	This study
TX16-034	vber26	vber	S	12.472	0.965	14.172	Riaz et al 2020
C 5-93	vber19	vber	R	13.102	0.948	12.536	This study
TX15-003	vber10	vber	S	13.206	0.965	14.906	Riaz et al 2020
TX16-035	vber27	vber	S	13.962	0.965	15.662	Riaz et al 2020
C 8-93	vber20	vber	S	14.022	0.949	12.958	This study
T21	vber02	vber	R	14.156	1.215	13.59	This study
TX15-059	vber11	vber	S	14.270	0.949	13.206	This study
T 03-05 S03	vber07	vber	S	14.782	0.950	14.754	This study
TX16-064	vcan26	vber	S	14.997	1.217	13.9333	This study
TX16-018	vber18	vber	S	15.285	1.071	16.985	Riaz et al 2020
TX16-032	vber14	vber	S	15.362	1.071	17.0625	Riaz et al 2020
T38	vber04	vber	S	15.852	1.061	15.77	This study
TX43-01	vber01	vber	S	16.148	1.219	16.0667	This study

TX15-063	vber23	vber	S	16.172	0.965	17.872	Riaz et al 2020
TX16-016	vber17	vber	S	16.346	0.965	18.046	Riaz et al 2020
TX16-022	vber21	vber	S	16.657	1.071	18.3575	Riaz et al 2020
TX16-065	vber28	vber	S	16.886	0.965	18.586	Riaz et al 2020
TX16-026	vber12	vber	S	17.016	0.965	18.716	Riaz et al 2020
TX16-030	vber13	vber	S	17.054	0.965	18.754	Riaz et al 2020
TX9722	vber05	vber	S	17.904	0.953	17.822	Riaz et al 2020
T2	vcan10	vcan	R	7.052	1.068	6.14	This study
T48	vcan03	vcan	R	9.499	0.554	8.6807	Riaz et al 2020
T45	vcan02	vcan	R	10.140	0.961	9.228	Riaz et al 2020
T46	vcan14	vcan	R	10.204	1.061	8.9825	Riaz et al 2020
candicans 9039	vcan31	vcan	R	10.441	1.483	9.875	This study
TX9715	vcan15	vcan	R	10.748	0.953	10.666	Riaz et al 2020
TX12-003	vcan09	vcan	R	10.999	0.870	9.935	This study
T62	vcan16	vcan	R	11.060	0.680	9.993	Riaz et al 2020
T73	vcan11	vcan	R	11.068	0.949	10.004	This study
T56	vcan08	vcan	R	11.115	0.879	11.5017	Riaz et al 2020
TX16-001	vcan24	vcan	R	11.152	0.949	10.088	This study
TX16-006	vcan25	vcan	R	11.216	0.949	10.152	This study
TX9703	vcan12	vcan	R	11.478	0.752	10.8188	Riaz et al 2020
T64	vcan17	vcan	R	11.491	1.063	11.17	Riaz et al 2020
T 03-08	vcan20	vcan	R	11.632	0.949	10.568	This study
T36	vcan13	vcan	R	11.718	0.645	10.8009	Riaz et al 2020
T 03-05	vcan19	vcan	R	12.318	0.949	11.254	This study
TX14-081	vcan23	vcan	R	12.386	0.949	11.322	This study
TX32-01	vcan04	vcan	R	12.617	0.954	11.396	Riaz et al 2020
T 03-13	vcan21	vcan	R	12.728	0.949	11.664	This study
candicans 9003	vcan29	vcan	R	12.808	0.948	12.242	This study
candicans 9001	vcan28	vcan	R	13.218	0.948	12.652	This study
candicans 9005	vcan30	vcan	R	13.310	0.948	12.744	This study
TX16-024	vcan27	vcan	R	13.319	0.870	12.255	This study
SC36	vgir27	vgir	R	9.368	0.539	8.8462	Riaz et al 2020
NV11-118	vgir12	vgir	R	9.754	0.953	9.672	This study
NV11-116	vgir11	vgir	R	10.340	0.953	10.258	This study
SC53	vgir17	vgir	R	10.728	0.949	9.664	This study
SC30	vgir22	vgir	R	10.857	0.879	11.2433	Riaz et al 2020
SC51	vgir16	vgir	R	11.352	0.949	10.288	This study
SC39	vgir26	vgir	R	11.460	0.879	11.8467	Riaz et al 2020

SC26	vgir23	vgir	R	12.232	0.879	12.6183	Riaz et al 2020
NV11-115	vgir10	vgir	R	12.264	0.953	12.182	This study
ANU78	vgir02	vgir	R	13.300	0.879	13.6867	Riaz et al 2020
SC11	vgir25	vgir	R	14.202	0.879	14.5883	Riaz et al 2020
SC33	vgir21	vgir	S	14.544	1.217	13.48	This study
SC40	vgir20	vgir	R	14.673	0.939	13.816	This study
SC9	vgir06	vgir	S	16.426	0.949	15.362	This study
NV12-040	vgir01	vgir	S	16.536	0.953	16.454	This study
NV12-041	vgir14	vgir	S	16.669	1.061	16.5875	This study
NV12-050	vgir15	vgir	S	16.814	1.058	15.75	This study
T 03-02 S01	vmon04	vmon	R	11.248	0.958	11.634	This study
T40	vmon02	vmon	R	12.520	0.958	12.906	Riaz et al 2020
C20-93-A	vmon05	vmon	R	13.572	0.948	13.006	This study
monticola 9040	vmon07	vmon	R	13.948	0.948	13.382	This study
TX67-03	vmon01	vmon	S	14.244	0.972	13.068	Riaz et al 2020
TXNM0823	vrip16	vrip	R	12.131	0.939	11.274	This study
NM12-111	vrip06	vrip	R	14.243	0.939	13.386	This study
NM12-104	vrip18	vrip	S	14.588	0.953	14.506	This study
NM12-119	vrip21	vrip	S	15.080	0.953	14.998	This study
KS14-035	vrip11	vrip	S	15.404	0.948	14.838	This study
OK14-064	vrip13	vrip	S	15.502	0.949	14.438	This study
CO12-103	vrip02	vrip	S	15.681	0.939	14.824	This study
KS14-036	vrip12	vrip	S	15.754	0.948	15.188	This study
NM12-108	vrip19	vrip	S	15.834	0.949	14.77	This study
NM12-116	vrip08	vrip	S	16.719	0.859	15.8617	This study
OK14-026	vrip01	vrip	S	16.904	1.058	15.84	This study
NM12-117	vrip09	vrip	S	17.080	0.953	16.998	This study
OK14-027	vrip10	vrip	S	17.109	1.225	17.8467	This study
TXNM0824	vrip17	vrip	S	17.139	0.939	16.282	This study
V28-96	vrip05	vrip	S	17.282	1.492	16.37	This study
V23-98	vrip04	vrip	S	17.477	1.492	16.565	This study
NM12-114	vrip07	vrip	S	17.899	0.939	17.042	This study
CO12-102	vrip14	vrip	S	18.290	0.953	18.208	This study
KS14-043	vrip23	vrip	S	18.979	0.939	18.122	This study

1. Riaz et 2020 refers to ref. 121 in the main text.

Table S11. Enrichments of SNPs significantly associated with PD in pIRs. Significance was calculated with randomization tests of 10,000 permutations.

Trio	Relative enrichment	Randomization p-value
GAM	**2.19**	**9.00E-04**
ARC	**2.47**	**1.19E-02**
GRC	**2.47**	**3.37E-02**
RAM	**1.12**	5.17E-01
GRB	**1.03**	8.13E-01
ARB	**1.09**	8.47E-01
GRM	0.81	9.14E-01
AGR	0.80	9.39E-01
MBC	0.59	9.80E-01

Table S12. Enrichments of SNPs associated to the the top three bioclimatic variables per trio in pIRs. Significance was calculated with randomization tests of 10,000 permuations.

**Trio**	**Sp. Assoc**	**BIO**	**Relative enrichment**	**Randomization (p-value)**
AGR	*V. girdiana*	2	**1.80**	**0.00E+00**
AGR	*V. girdiana*	6	**1.15**	1.00E+00
AGR	*V. girdiana*	17	**1.30**	8.15E-01
ARB	*V. riparia*	1	**1.34**	**0.00E+00**
ARB	*V. riparia*	2	**1.31**	**0.00E+00**
ARB	*V. riparia*	6	**1.18**	1.48E-01
ARC	*V. riparia*	1	**1.07**	**2.65E-02**
ARC	*V. riparia*	2	**1.28**	**0.00E+00**
ARC	*V. riparia*	6	**1.06**	**8.70E-03**
GAM	*V. arizonica*	6	**1.37**	**0.00E+00**
GAM	*V. arizonica*	11	**1.06**	1.00E+00
GAM	*V. arizonica*	14	**1.38**	**0.00E+00**
GRB	*V. riparia*	1	**1.07**	6.06E-01
GRB	*V. riparia*	2	**1.24**	**0.00E+00**
GRB	*V. riparia*	6	**1.08**	5.47E-01
GRC	*V. riparia*	1	**1.34**	**2.00E-04**
GRC	*V. riparia*	2	**1.46**	**0.00E+00**
GRC	*V. riparia*	6	**1.21**	6.61E-02
GRM	*V. riparia*	1	**1.12**	2.96E-01
GRM	*V. riparia*	2	**1.15**	7.95E-02
GRM	*V. riparia*	6	**1.12**	1.90E-01
MBC	*V. berlandieri*	2	**1.77**	**0.00E+00**
MBC	*V. berlandieri*	9	**2.13**	**0.00E+00**
MBC	*V. berlandieri*	11	**2.15**	**0.00E+00**
RAM	*V. arizonica*	6	**1.93**	**0.00E+00**
RAM	*V. arizonica*	11	**1.07**	1.00E+00
RAM	*V. arizonica*	14	**1.52**	**0.00E+00**
