# Supplementary material for: Introgression among North American wild grapes (Vitis) fuels biotic and abiotic adaptation
Source: Genome Biol. 2021 Sep 3;22:254. doi: 10.1186/s13059-021-02467-z (PMC8414701; doi:10.1186/s13059-021-02467-z)
Supplement: Supplementary file 2 — Additional file 2: Figure S1. Genetic clustering of all samples (including hybrid samples) detected by the structure analyses from K = 2 to K = 10. Figure S2. Consensus maximum likelihood phylogenetic tree created with IQtree using 15,893 informative sites found by SNPphylo. Figure S3. Clock calibrated phylogenetic tree created with BEAST using 15,893 informative sites found by SNPphylo. Figure S4. Results of the Fbranch statistic using species tree models. Figure S5. Pairs of species that show evidence of introgression present evidence of overlap at three periods: present, Holocene, Pleistocene. Figure S6. Pairs of species that do not show evidence of introgression present no or low overlap at three periods: present, Holocene, Pleistocene. Figure S7. Pairs of species that do not show evidence of introgression but have overlapping distributions at three periods: present, Holocene, Pleistocene. Figure S8. Genomic size distribution of the windows used to calculate the ABBA-BABA statistics per trio. Figure S9. Distribution the fdM statistic values across windows per trio. Figure S10. Distribution the fd statistic values across windows per trio. Figure S11. Manhattan plot of all the middle point value all windows with fdM values for all nine trios. Figure S12. Scatterplot showing correlations between the fdM and fd statistics across all windows in the genome. Figure S13. Mean gene density of across the whole genome and within the pIRs of all the trios. Figure S14. Average weights of phylogenetic relationships in the pIRs. SNPs from pIRs for each trio were split in 10Kb windows to create a phylogenetic tree for each window. Figure S15. Principal component variance LFMM association analyses. Figure S16. QQ plots showing the logarithm of the p-values for a linear regression model, the LFMM model using 7 LF and without controlling for a genome inflation factor and controlling for a genome inflation factor. Figure S17. Manhattan plot of LFMM association results for the conce [file 13059_2021_2467_MOESM2_ESM.pdf]

Additional File 2: Supplementary Figures S1-S19 for

Extensive introgression among North American wild grapes (*Vitis*) fuels biotic and abiotic adaptation

Abraham Morales-Cruz[1], Jonas Aguirre-Ligouri[1], Yongfeng Zhou[1], Andrea Minio[2], Summaira Riaz[2], Andrew M. Walker[2], Dario Cantu[2], and Brandon S. Gaut*[2]

[1] Dept. Ecology and Evolutionary Biology, University of California Irvine, Irvine, CA

[2] Dept. of Viticulture, University of California, Davis, Davis, CA

[*]Address for Correspondence: Brandon Gaut, Department of Ecology and Evolutionary Biology, 321 Steinhaus Hall, UC Irvine, 92697; Email: bgaut@uci.edu; Phone: (949) 677-06972

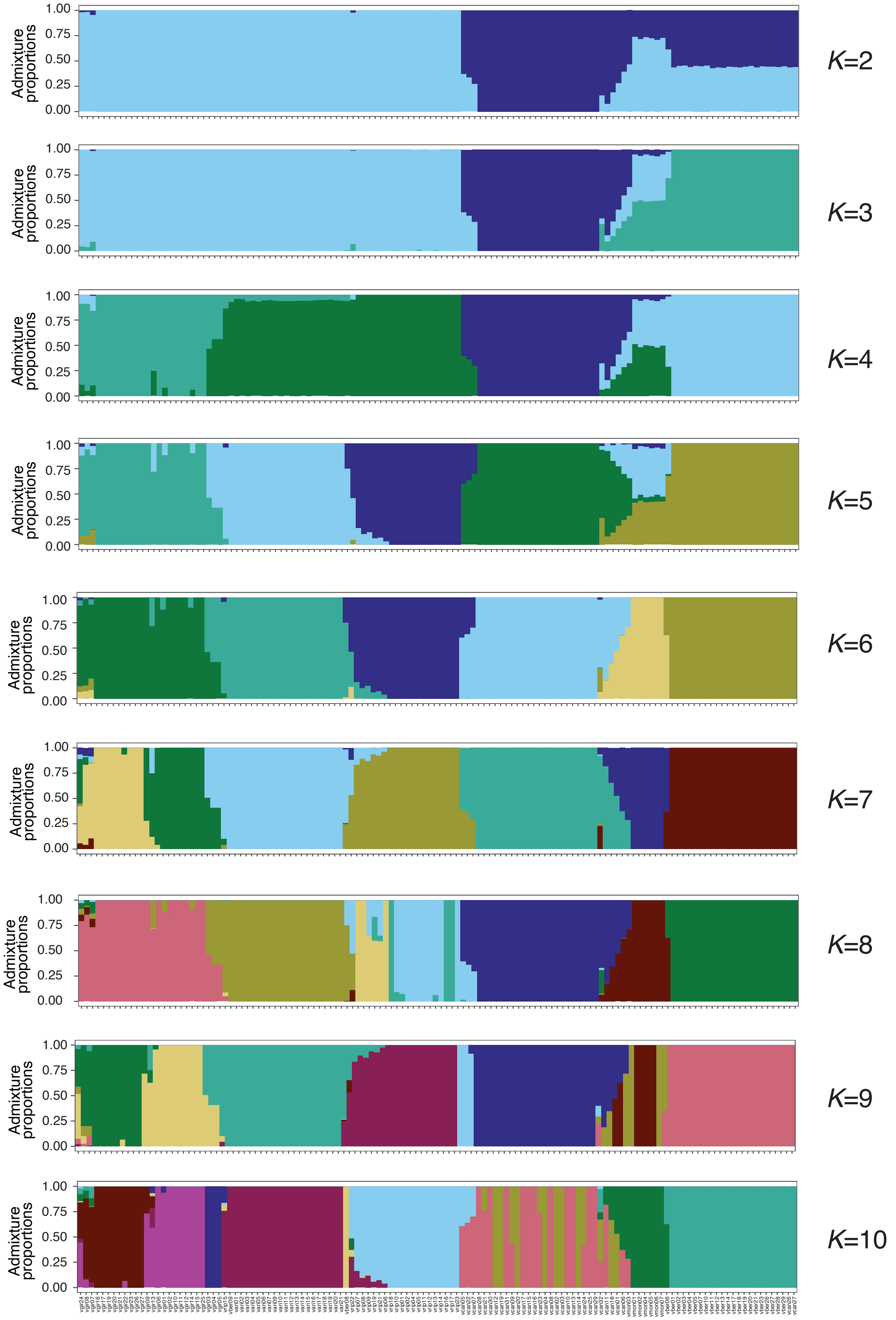

Figure S1. Genetic clustering of all samples (including hybrid samples) detected by the structure analyses from K=2 to K=10.

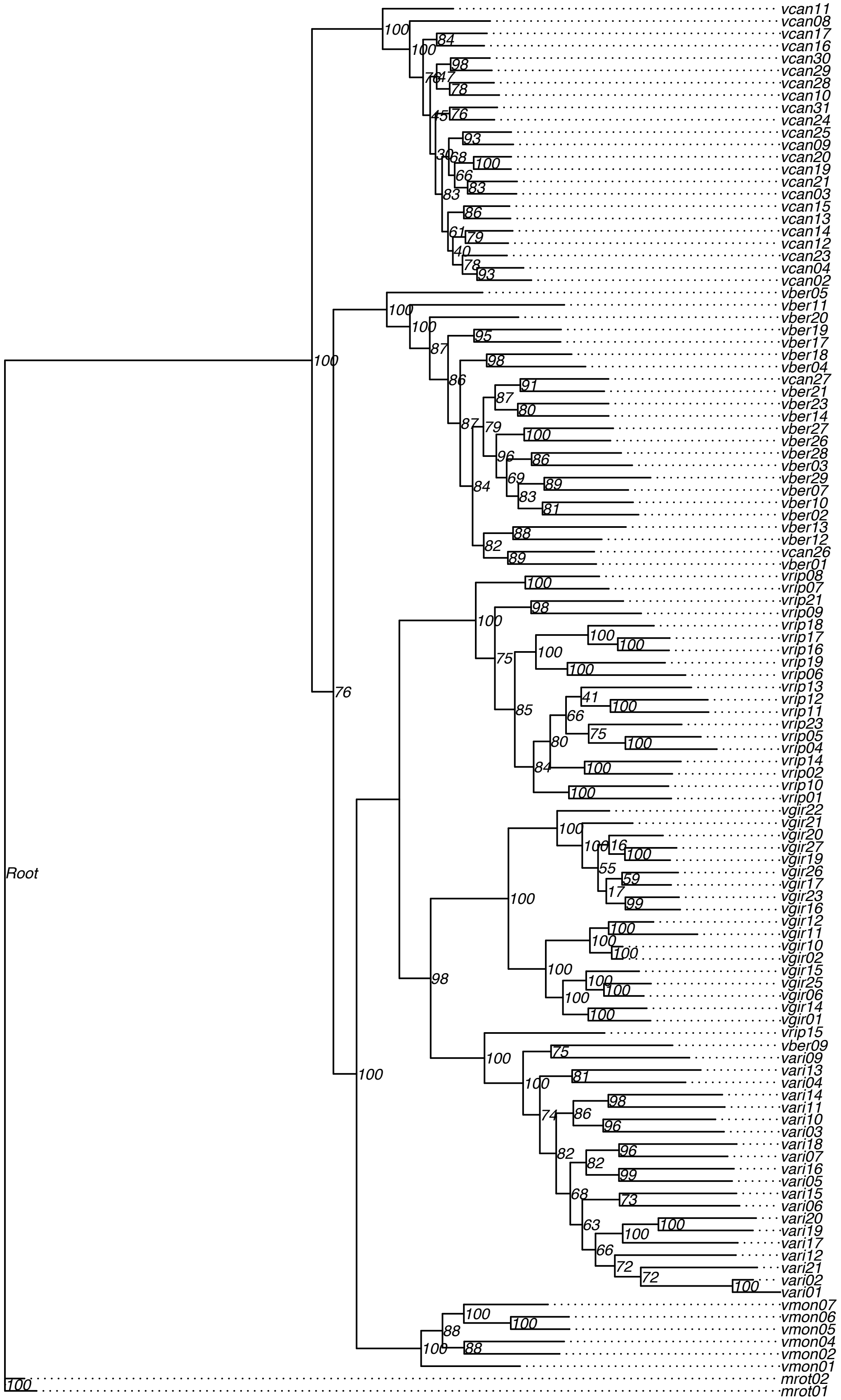

Figure S2. Consensus maximum likelihood phylogenetic tree created with IQtree using 15,893 informative sites found by SNPphylo. Numbers on the nodes correspond to bootstrap support values from 1000 replicates.

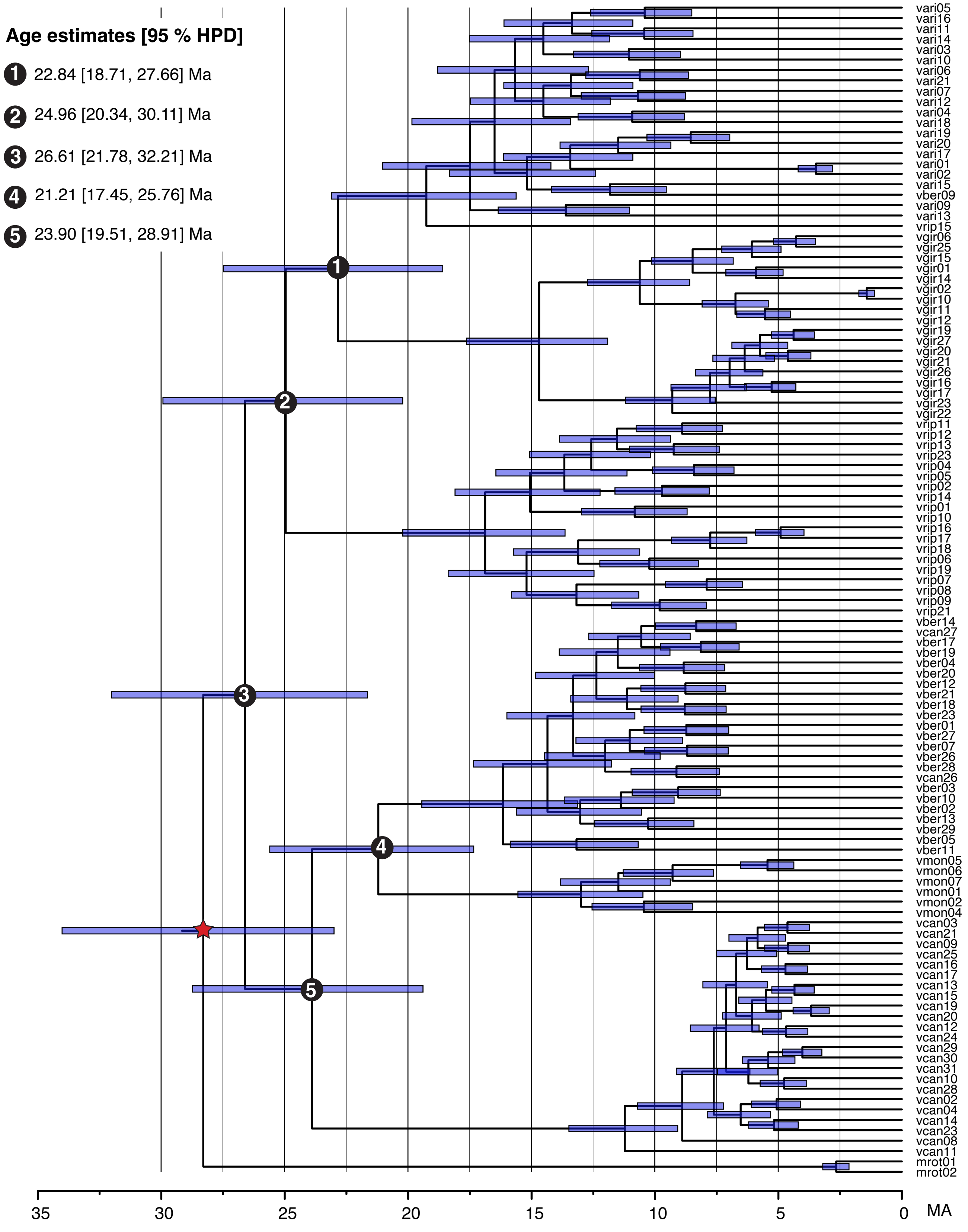

Figure S3. Clock calibrated phylogenetic tree created with BEAST using 15,893 informative sites found by SNPphylo. BEAST was run in a chain length of 10 million with sampling every 500. A 10% burin was used to create the final tree. The node bars correspond to the 95% confidence intervals of the Highest Posterior Density (HPD). The star represents the calibration point based on Wan et al 2013. Scale shows million years ago (MA).

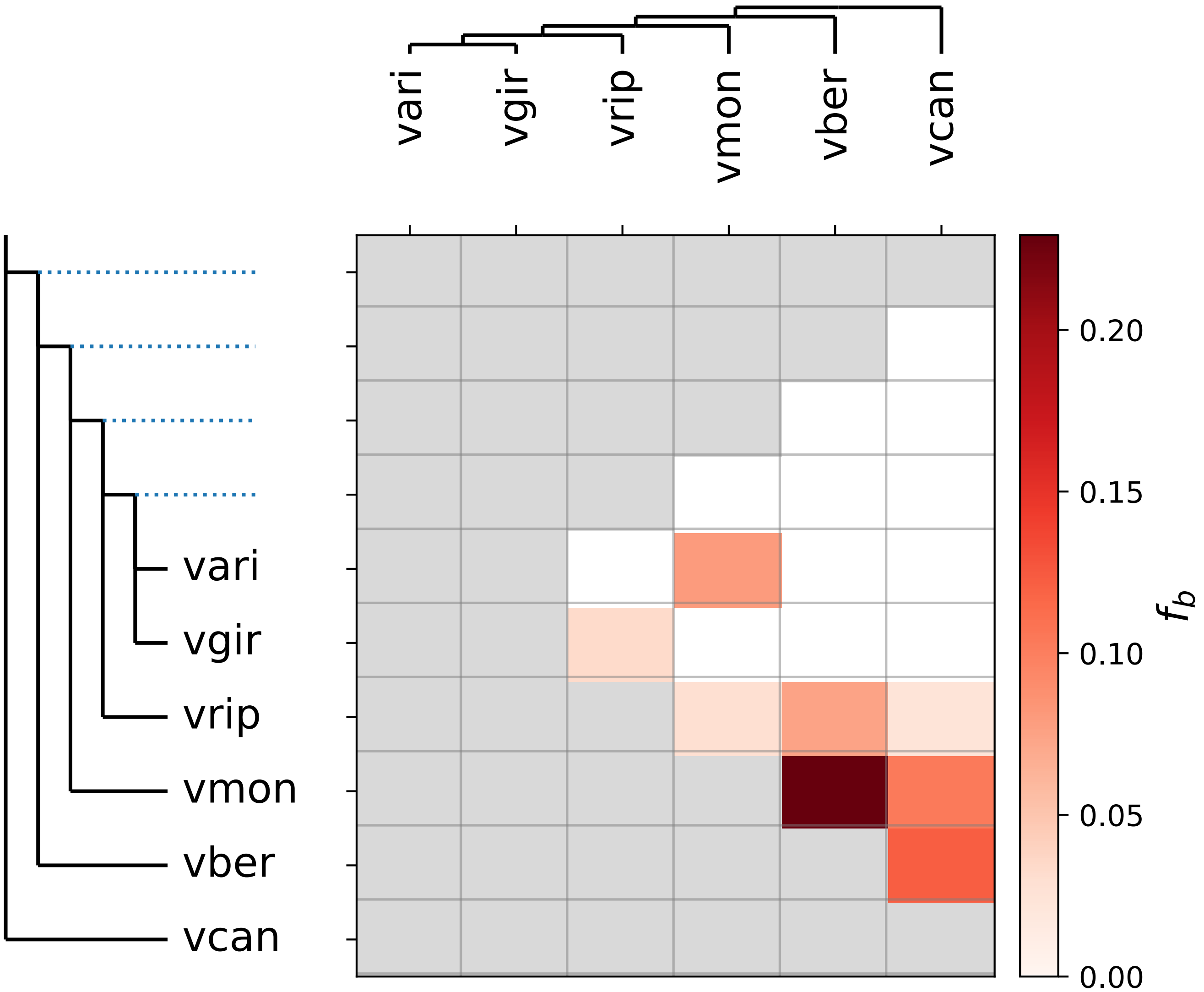

Figure S4. Results of the Fbranch statistic using species tree models. Only comparisons with f4-ratios significantly (p < 0.01) higher than zero were evaluated. Positive values of the f_b statistic in nodes would indicate complex history and potential evidence of gene flow in specific internal branches.

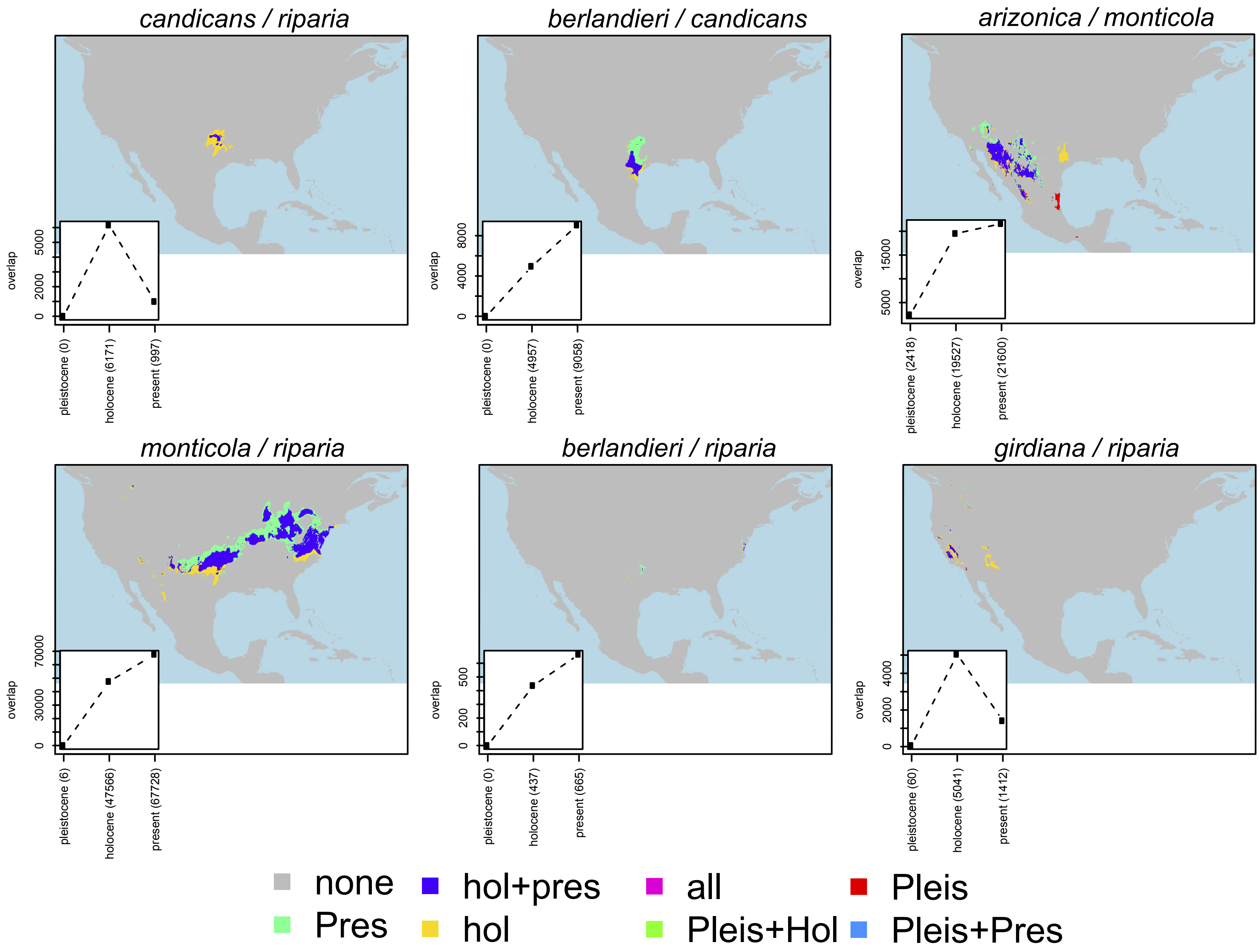

Figure S5. Pairs of species that show evidence of introgression present evidence of overlap at three periods: present, Holocene, Pleistocene. The overlap corresponds to the number of overlapped pixels from the raster objects at a 2.5 arcseconds.

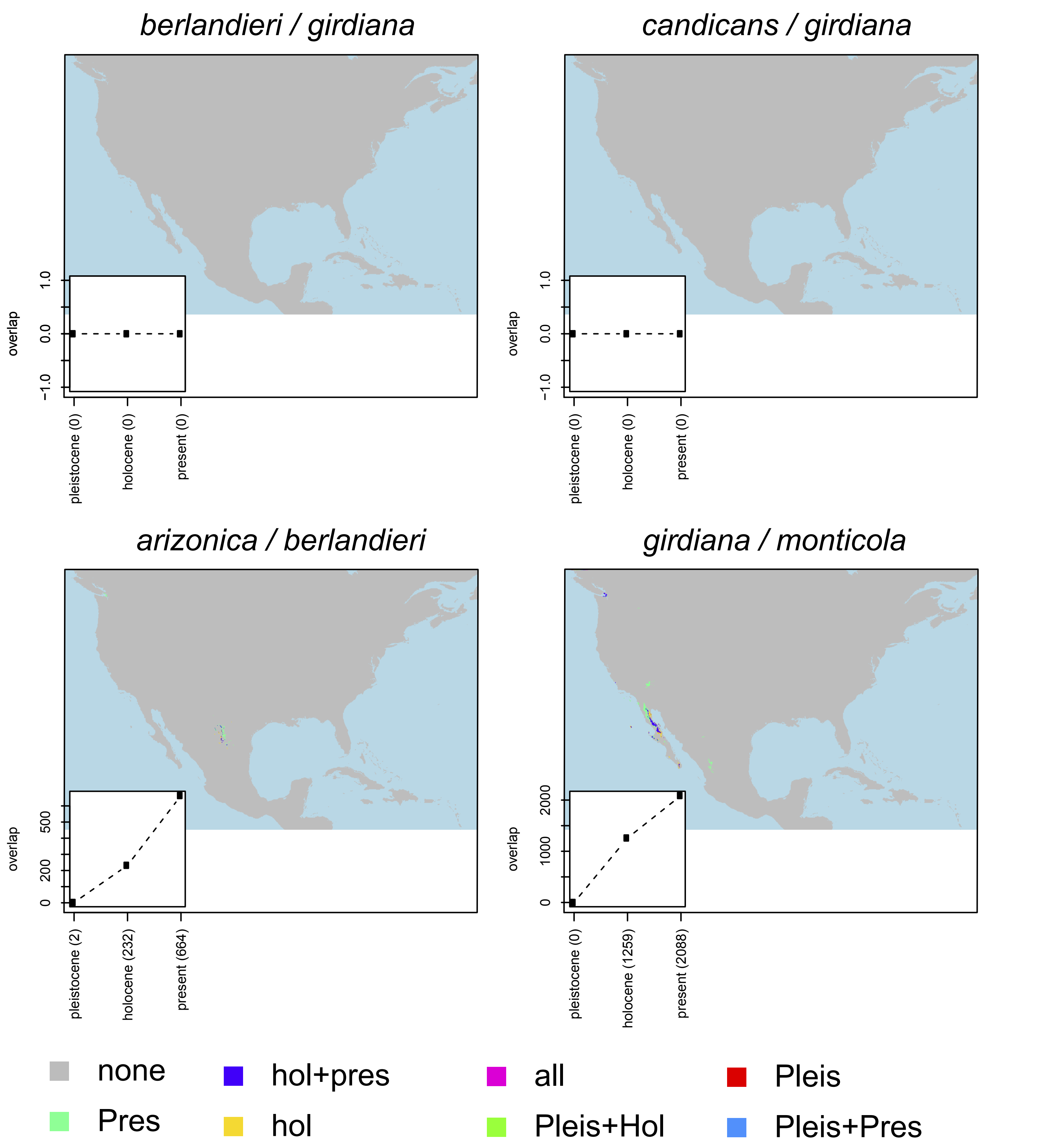

Figure S6. Pairs of species that do not show evidence of introgression present no or low overlap at three periods: present, Holocene, Pleistocene. The overlap corresponds to the number of overlapped pixels from the raster objects at a 2.5 arcseconds

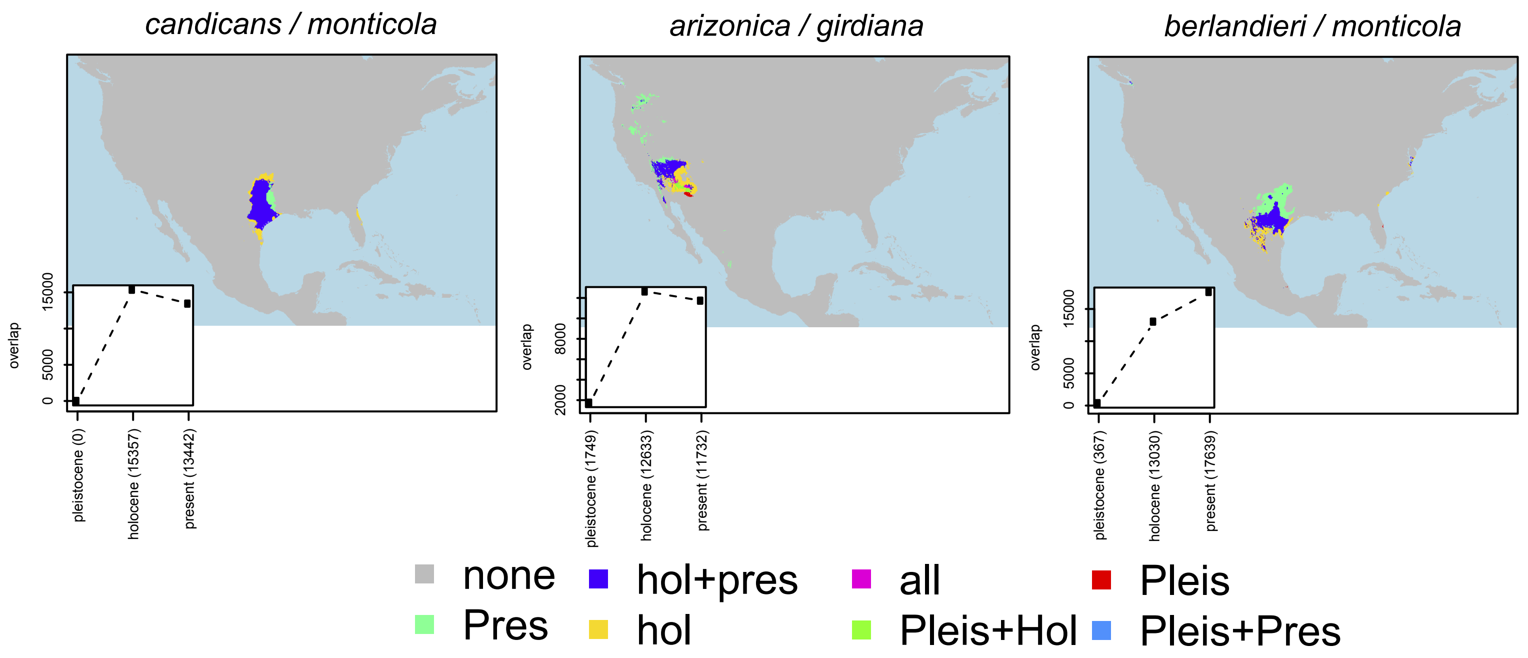

Figure S7. Pairs of species that do not show evidence of introgression but have overlapping distributions at three periods: present, Holocene, Pleistocene. The overlap corresponds to the number of overlapped pixels from the raster objects at a 2.5 arcseconds.

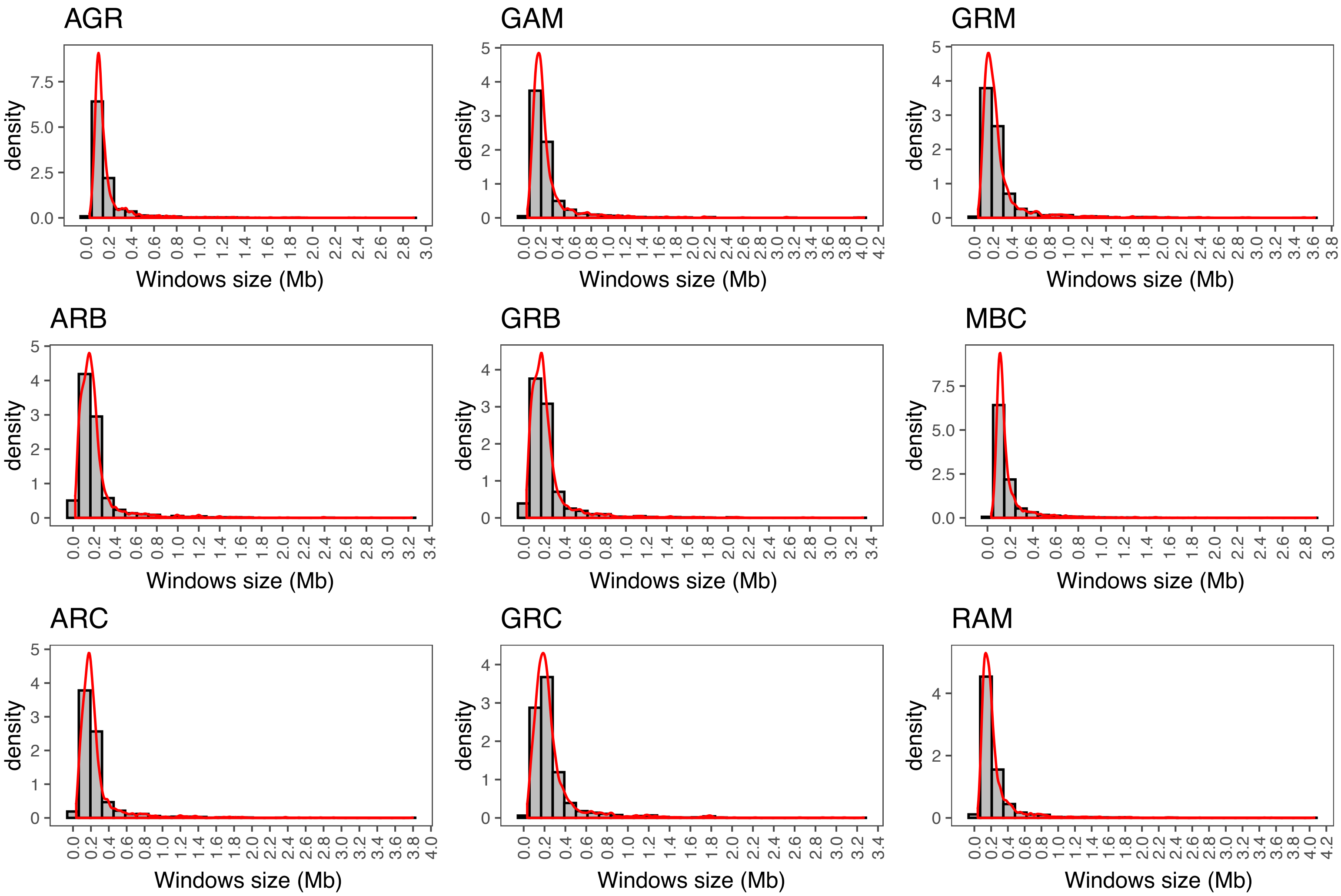

Figure S8. Genomic size distribution of the windows used to calculate the ABBA-BABA statistics per trio.

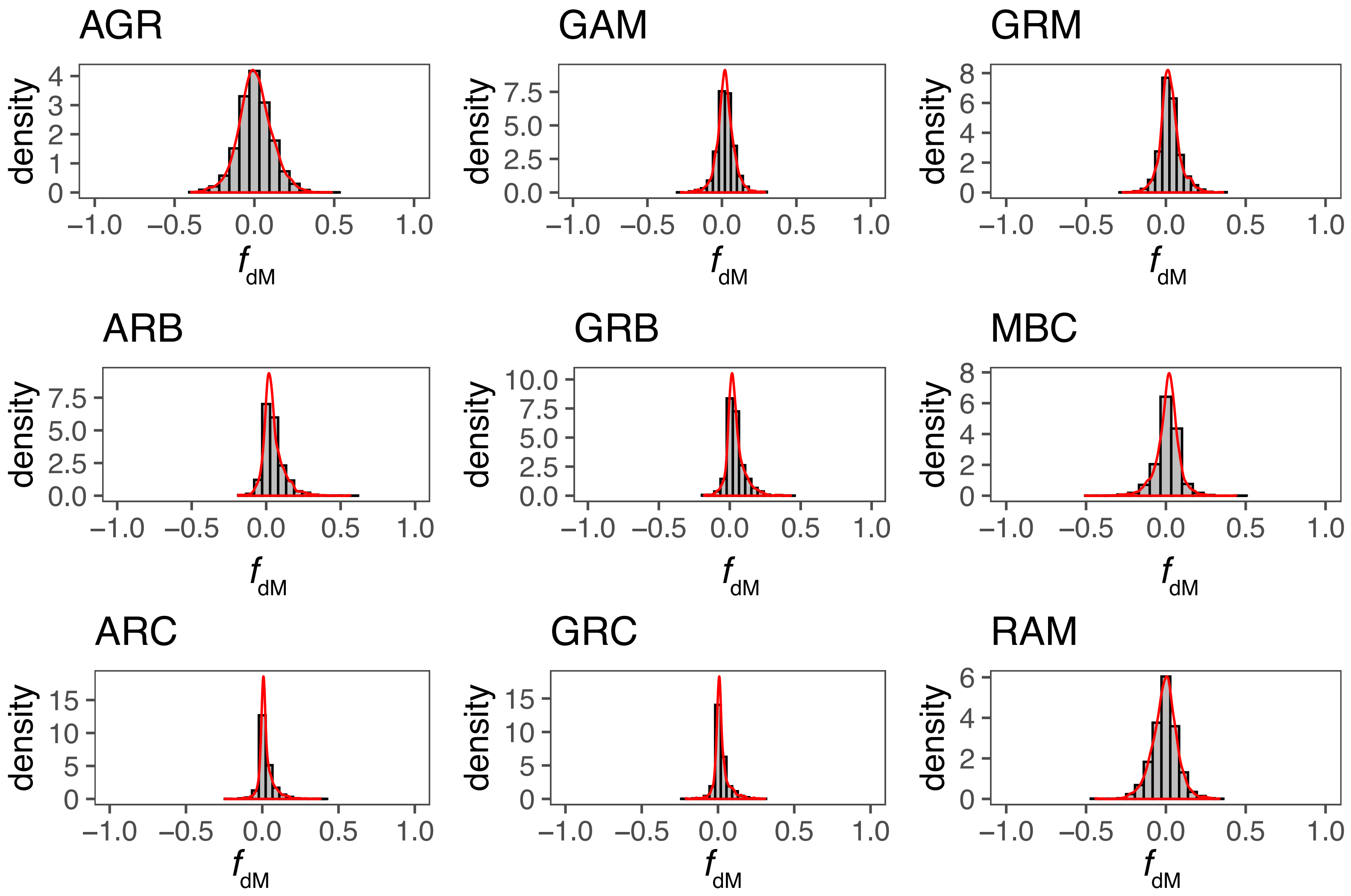

Figure S9. Distribution the f_{dM} statistic values across windows per trio**.**

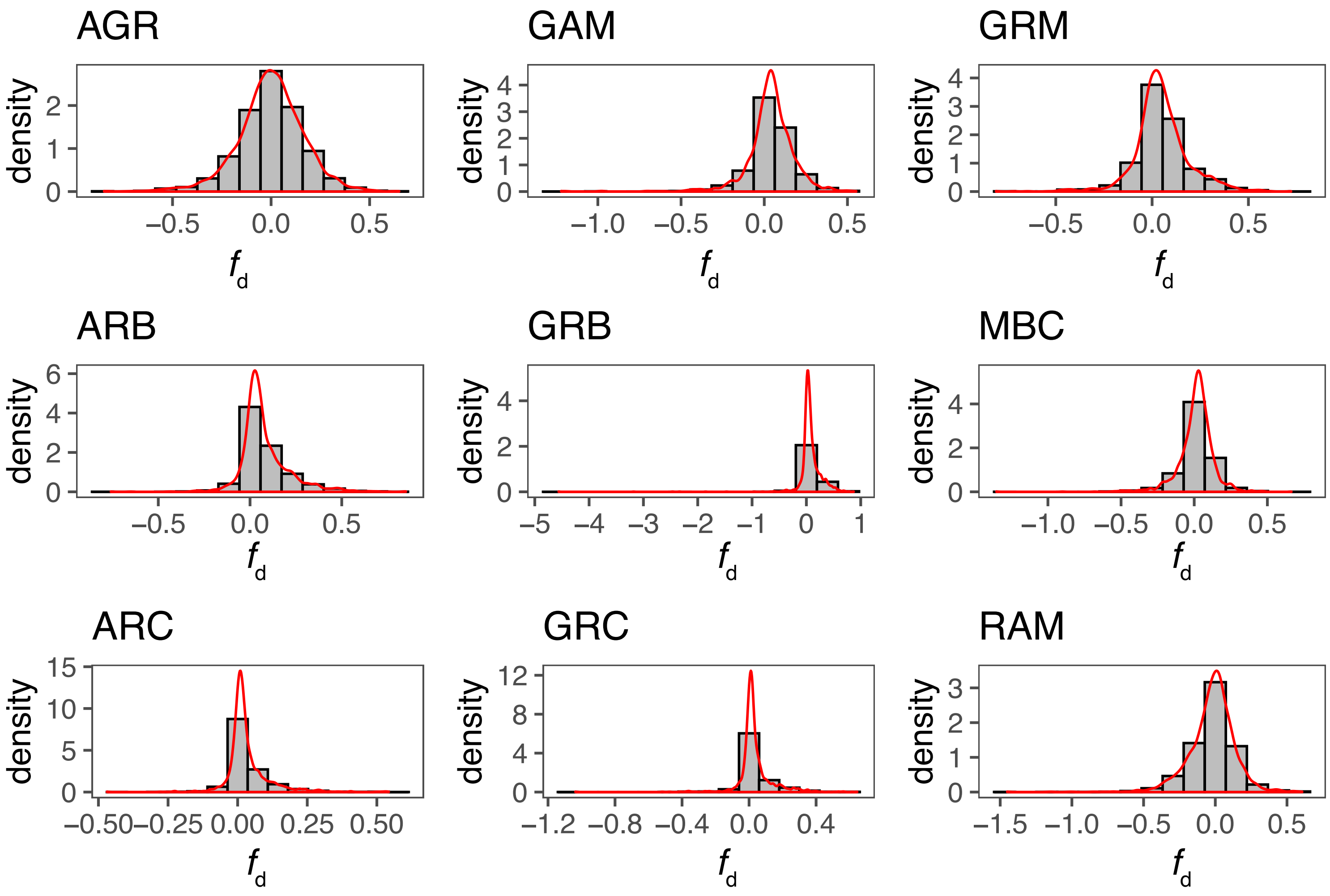

Figure S10. Distribution the f_d statistic values across windows per trio.

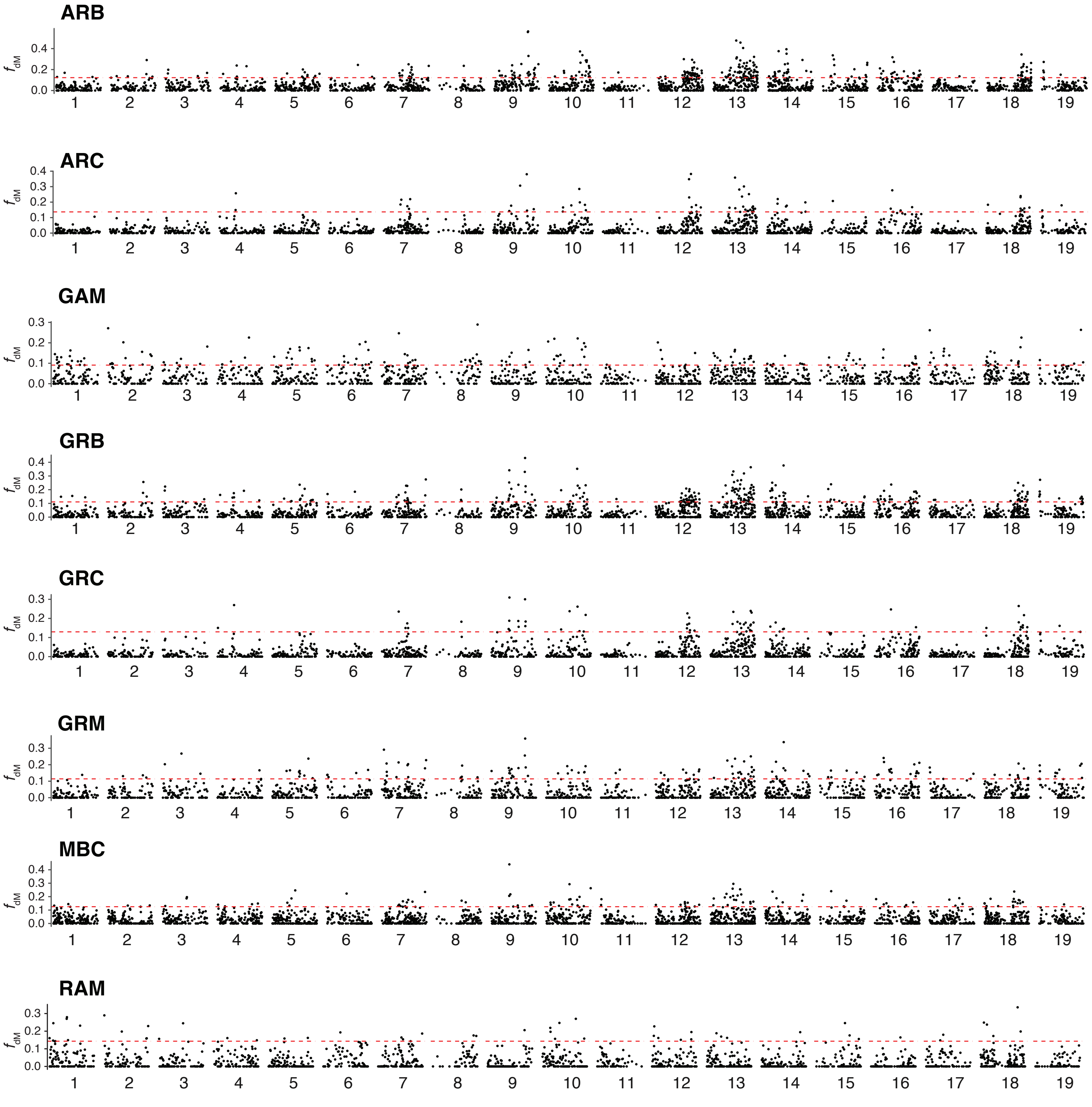

Figure S11. Manhattan plot of all the middle point value all windows with fdM values for all nine trios. The red dotted line corresponds to the cutoff imposed for each trio in function of the f4-ratio or the genome-wide estimate of introgression.

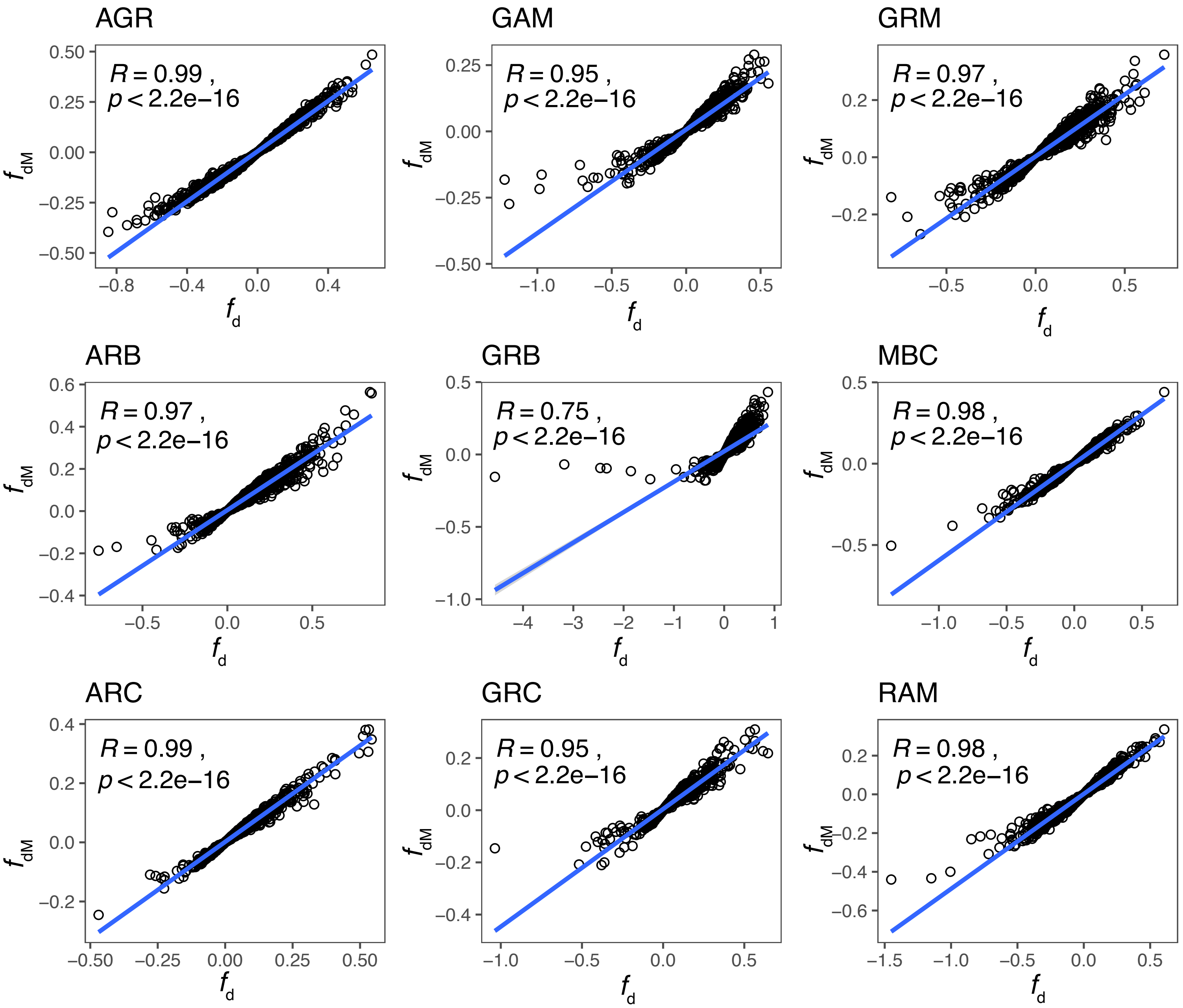

Figure S12. Scatterplot showing correlations between the f_{dM} and f_d statistics across all windows in the genome.

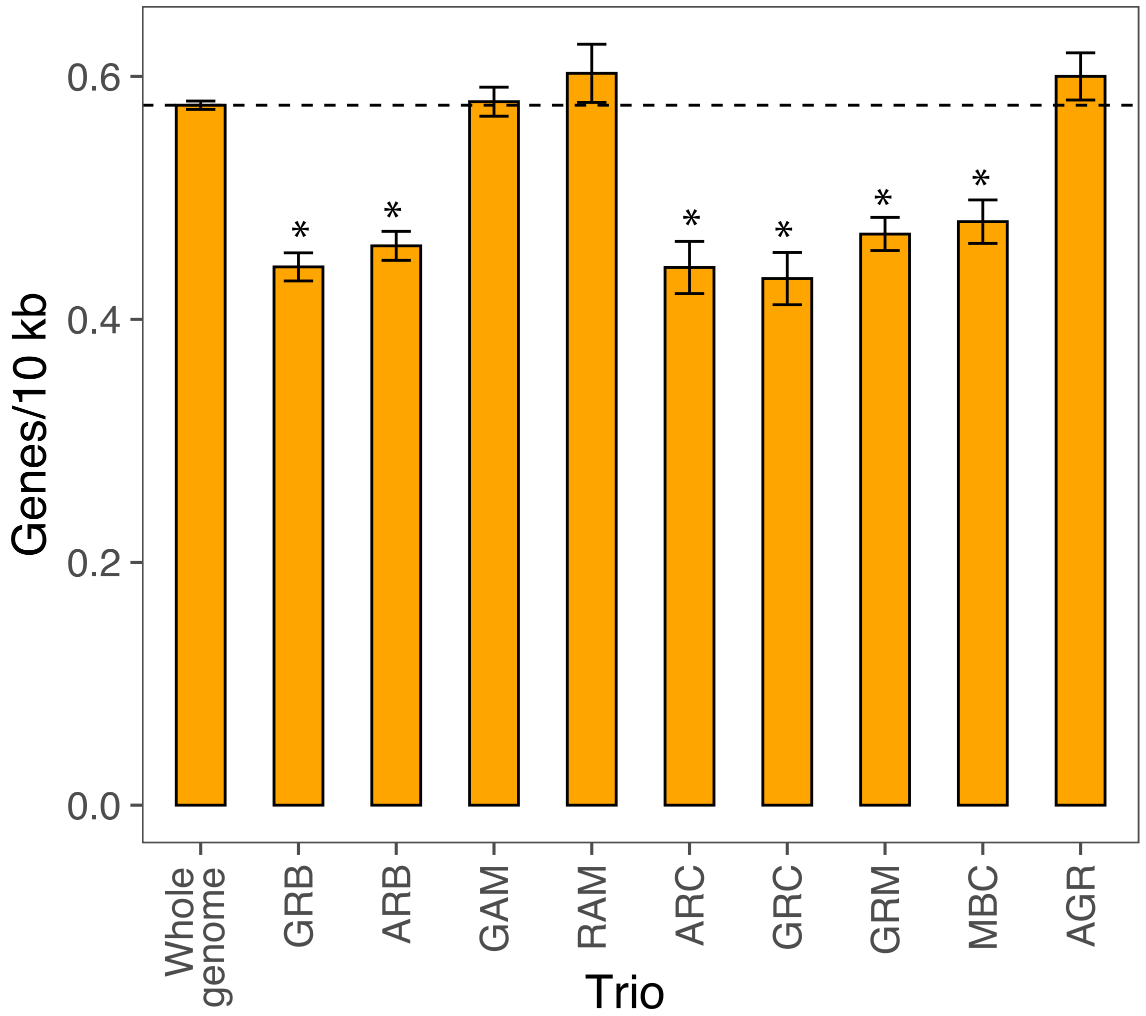

Figure S13. Mean gene density of across the whole genome and within the pIRs of all the trios. Error bars correspond to 1 SD. Dotted line corresponds the mean gene density of the whole genome. Asterisks denote trios with significantly lower gene density than the genome-wide average (t-test, $p < 0.001$).

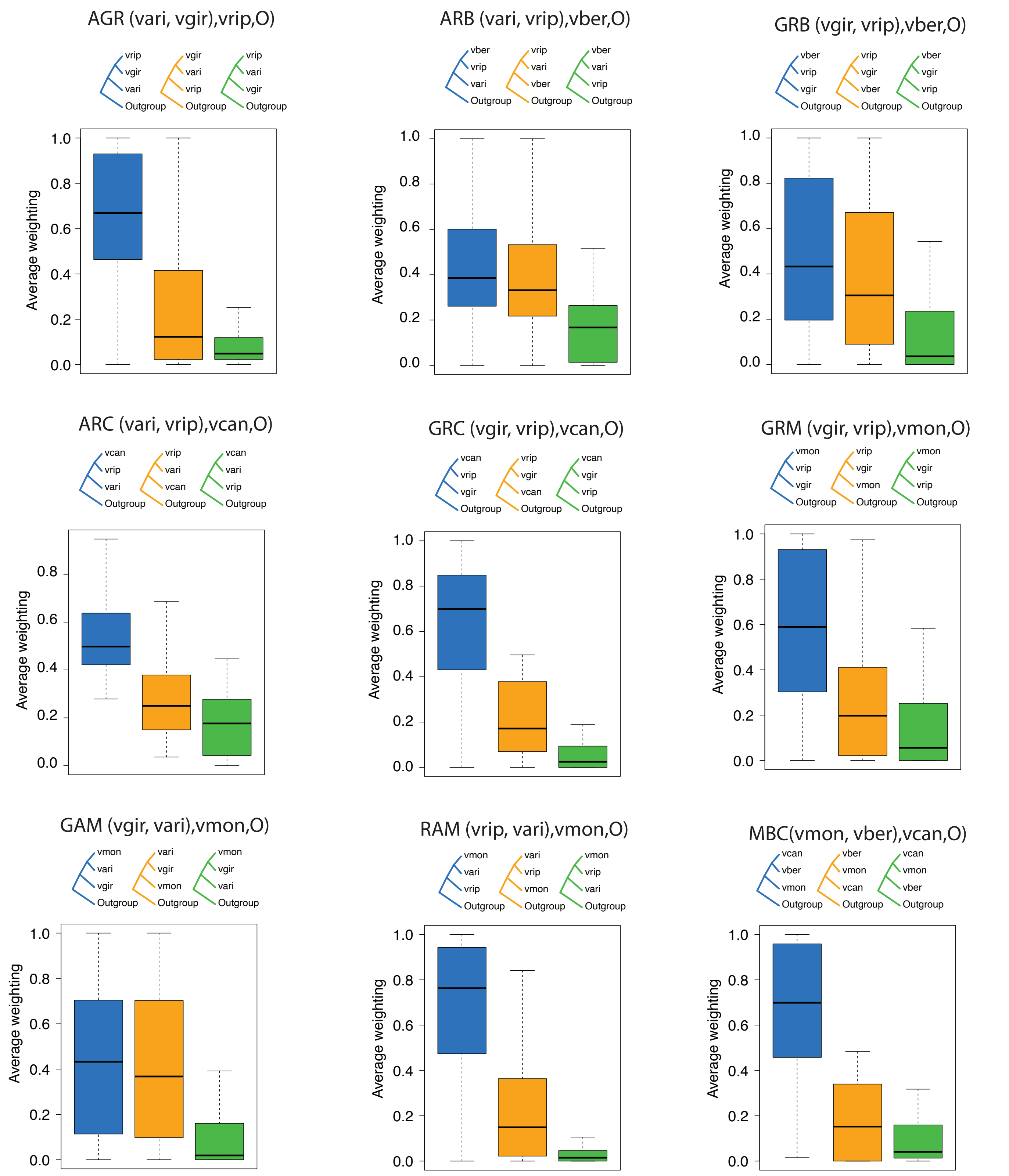

Figure S14. Average weights of phylogenetic relationships in the pIRs. SNPs from pIRs for each trio were split in 10Kb windows to create a phylogenetic tree for each window. In all trios, the phylogeny with the highest frequency supports the hypothesis of introgression because of the discordance to the consensus tree. In every trio, the phylogeny with the highest average weight shows a switch between the P2 and P3 species.

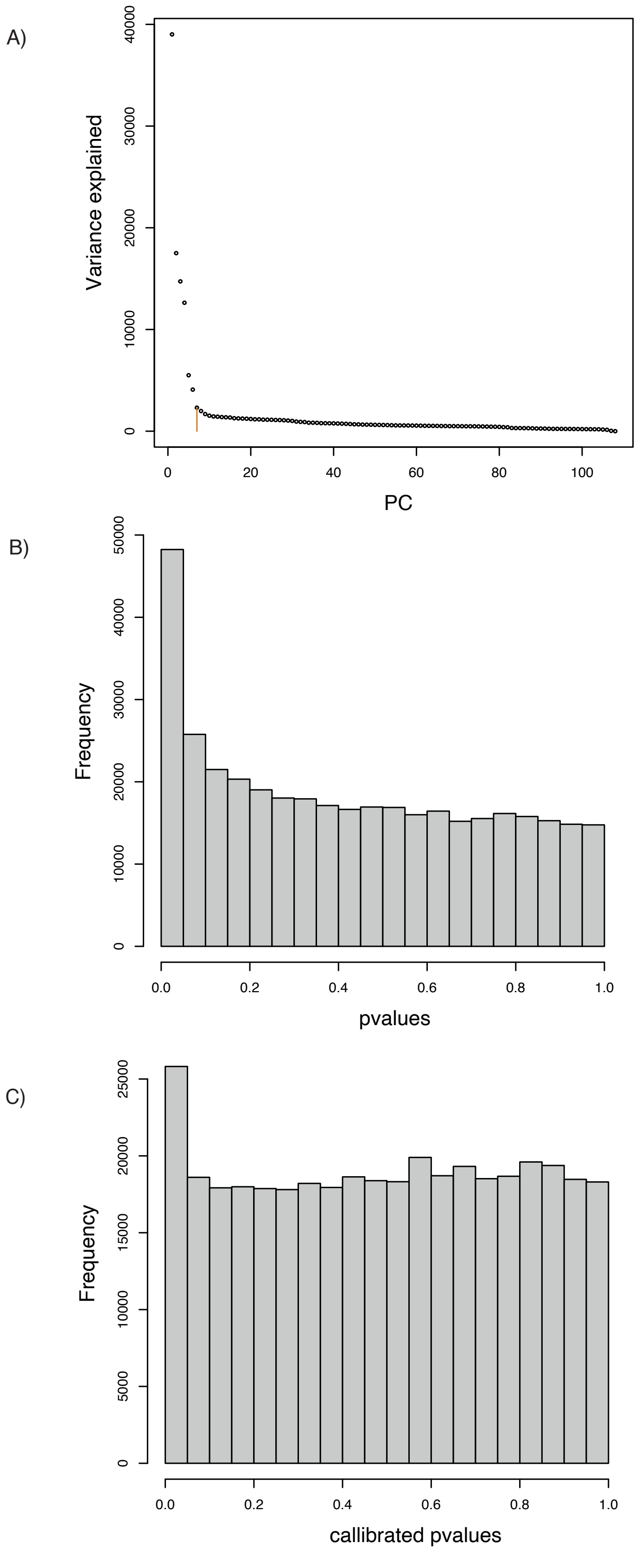

Figure S15. A) Screen plot showing the variance explained by different number of PCs. The orange arrow shows the “knee” where the variance explained by each PC becomes smaller. This suggest that 7 LF should be used in the LFMM models (this is also suggested by the Admixture analyses in **Figure 2A**). B) Distribution of p-values without controlling for the genome inflation factor. C) Distribution of p-values controlling for the genome inflation factor. This plot is expected to have a flat distribution with a peak near 0.

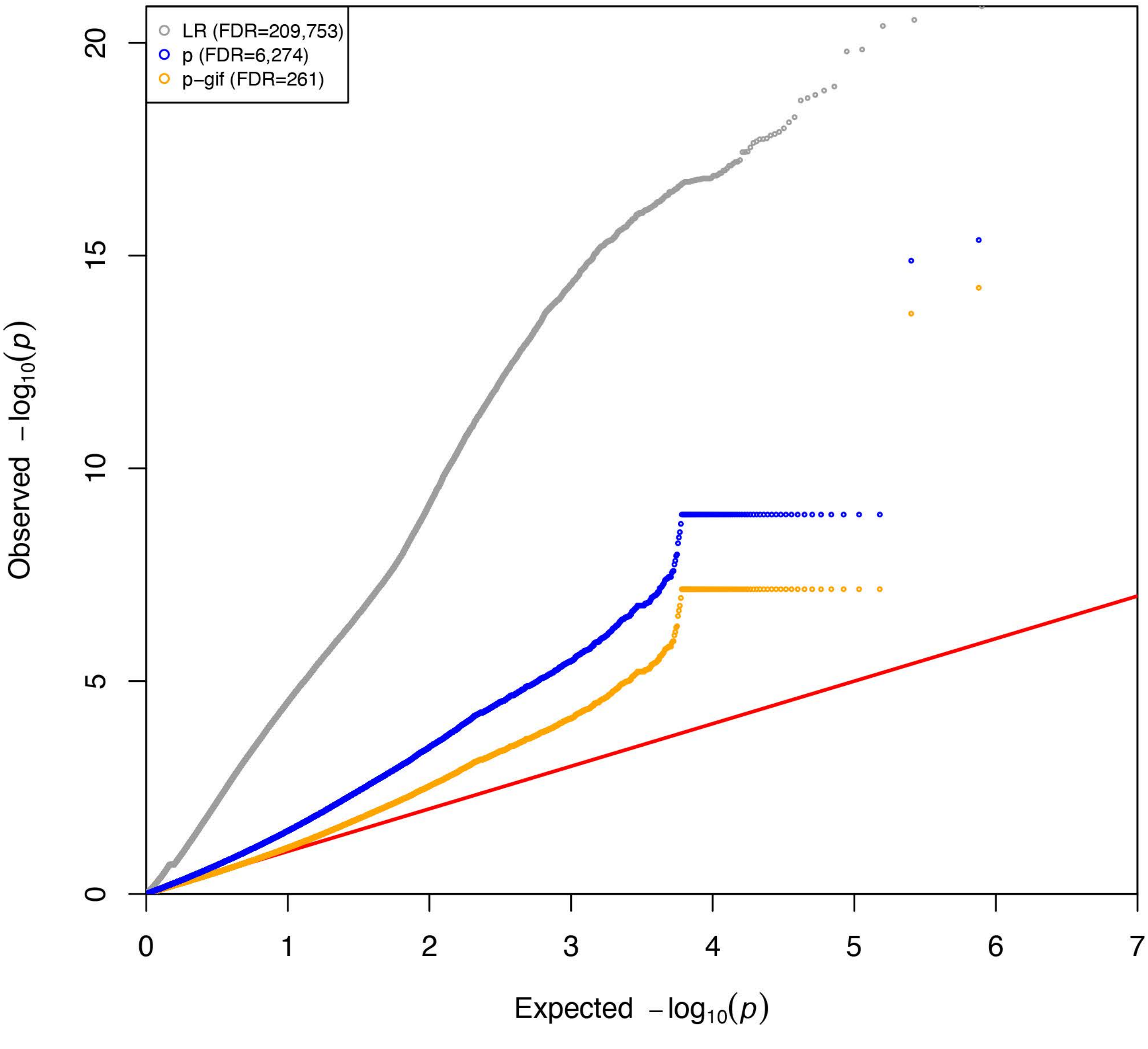

Figure S16. QQ plots showing the logarithm of the p-values for a linear regression model (grey), the LFMM model using 7 LF and without controlling for a genome inflation factor (blue) and controlling for a genome inflation factor (orange). The red line shows the slope=1 line.

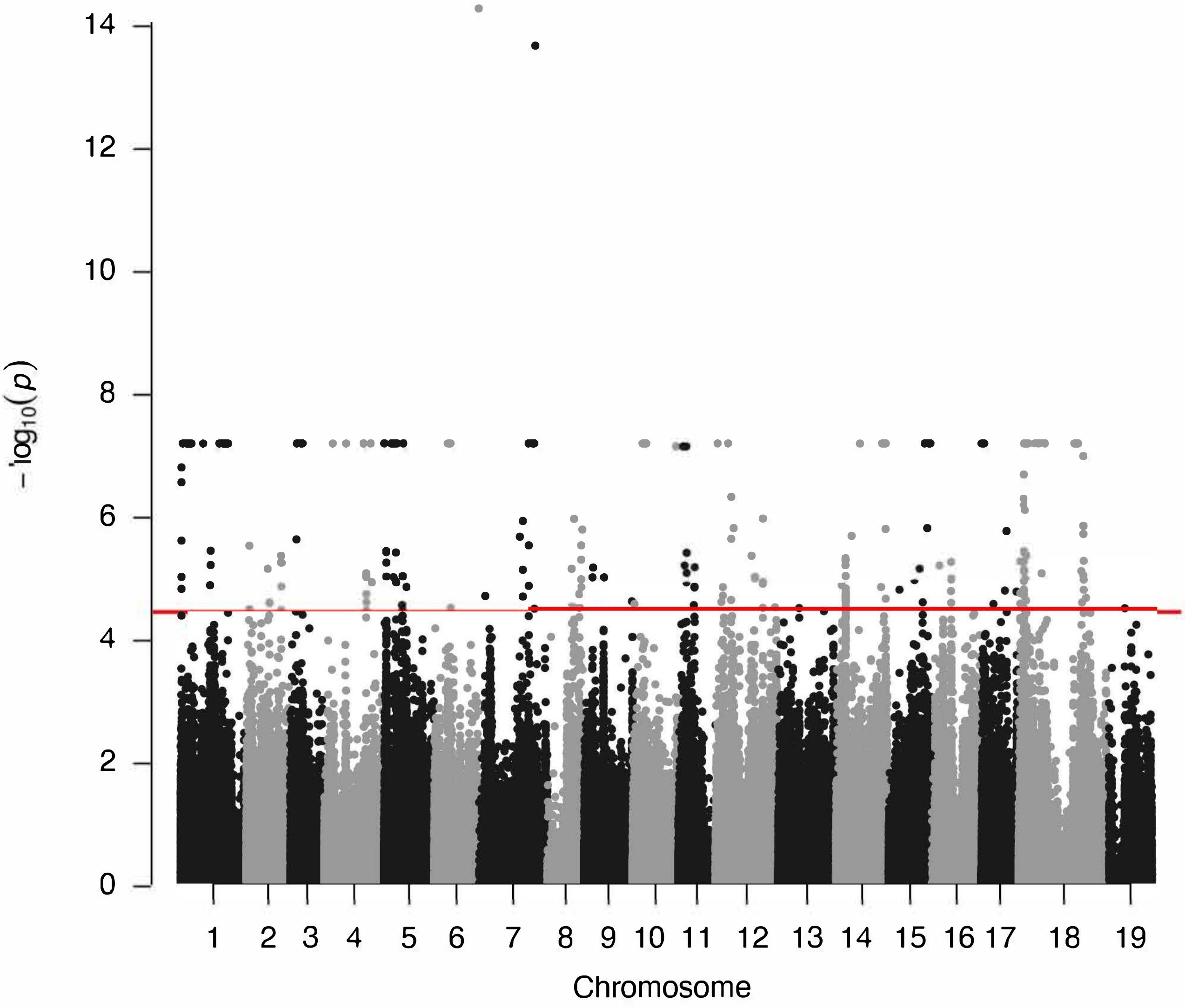

Figure S17. Manhattan plot of LFMM association results for the concentration of *Xyllela fastidiosa*. The red lines indicate the threshold at an FDR<0.05.

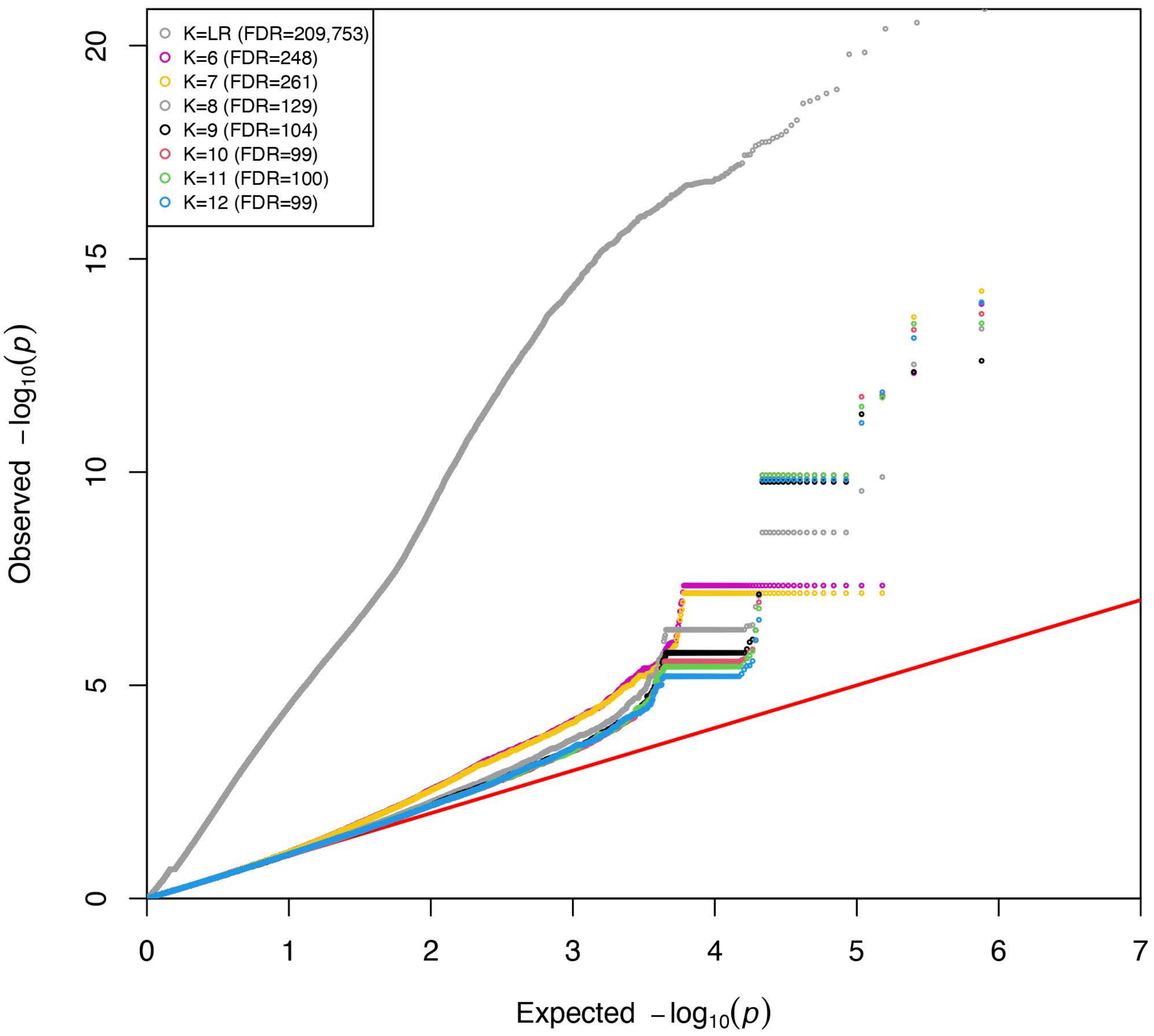

Figure S18. QQ plots showing the logarithm of the p-values for a linear regression model (grey), and LFMM models using 6-12 LF and controlling for a genome inflation factor. The red line shows the slope=1 line.

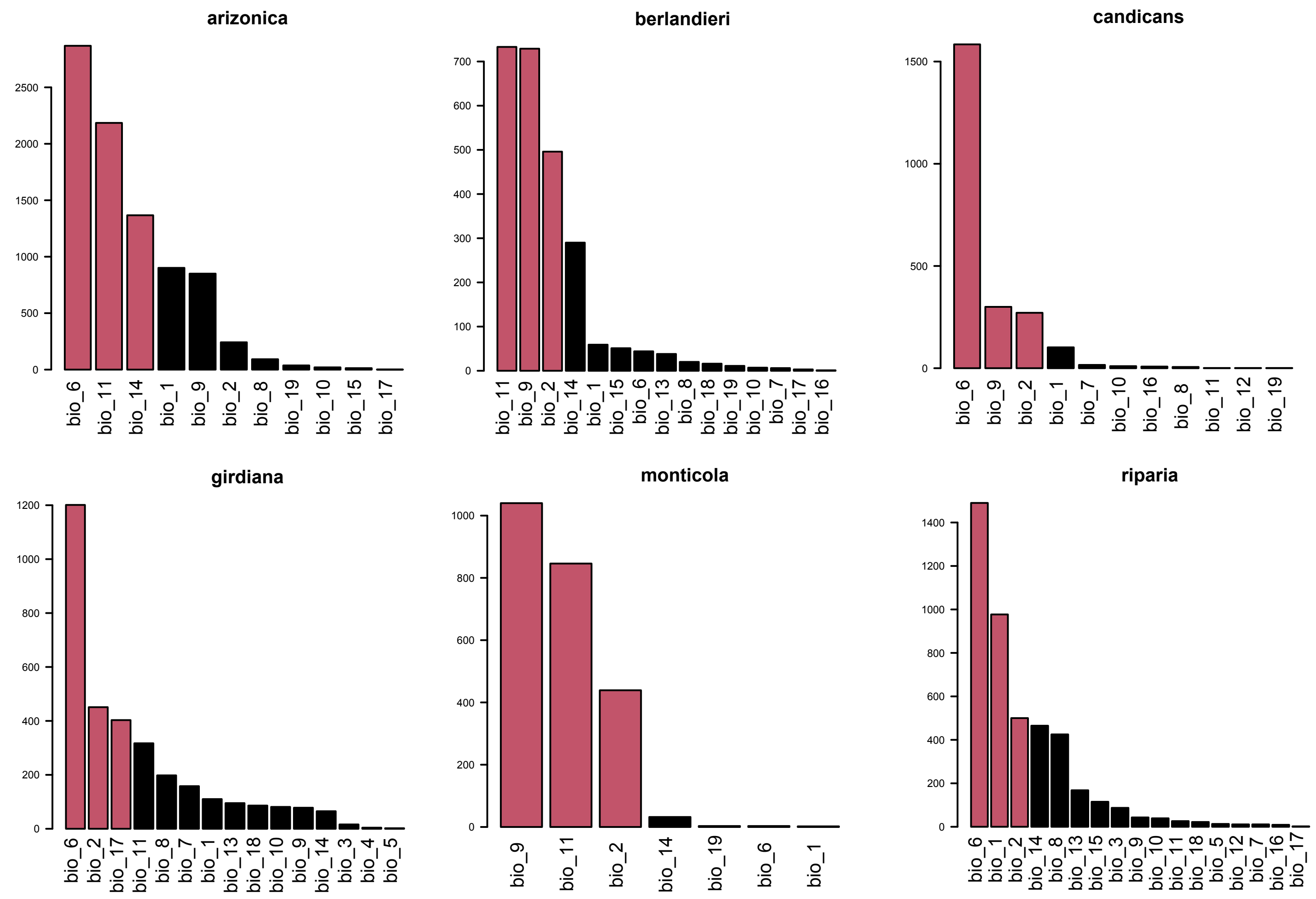

Figure S19. Number of associated candidate SNPs per bioclimatic variables per species. Red bars indicate the three Bioclimatic variables with the higher number of associated SNPs.
