# Supplementary material for: Introgression among North American wild grapes (Vitis) fuels biotic and abiotic adaptation
Source: Genome Biol. 2021 Sep 3;22:254. doi: 10.1186/s13059-021-02467-z (PMC8414701; doi:10.1186/s13059-021-02467-z)
Supplement: Supplementary file 3 — Additional file 3. Review history. [file 13059_2021_2467_MOESM3_ESM.docx]

Review History

**First round of review**

**Reviewer 1**

**Are you able to assess all statistics in the manuscript, including the appropriateness of statistical tests used?**

**Yes, and I have assessed the statistics in my report.**

**Comments to author:**

The authors provided valuable resources to the genetic research and breeding of grapevine by resequencing 130 wild accessions from six Vitis species in the Southwestern United States. They focused on the genome wide introgressions among Vitis species, however, the results of introgression events and the subsequent GWAS analysis are a little vague and lack of detailed proof process. The lack of solid functional genes also make the novelty relatively limited.

Major comments:

1. Line 86-87: "We generated a reference genome of V. arizonica produced from long-read sequences [30] that were assembled with the aid of an optical map."

The reference genome of V. arizonica mentioned here could not to be found in NCBI database. By the way, since the genome of V. arizonica had been reported in the previous paper (as Massonnet M, Cochetel N, Minio A, Vondras AM, Lin J, Muyle A, et al. The genetic basis of sex determination in grapes. Nat Commun. 2020; 11:2902.), the descriptions related to the genome sequencing, assembly and annotation in the manuscript should be removed. For instance, the description "The reference assembly contained 19 anchored pseudomolecules, an N50 of 25.9 Mb, a size of 503 Mb and a BUSCO score of 96.4% (Additional File 2: Table S1 & Additional File 3: Table S2)" will mislead readers.

In addition, in line 630, the raw data (BioProject ID: PRJNA607282 & PRJNA705722) could not be retrieved in NCBI.

2. Line 95-96: The K=2 to 10 clusters of genetic structure obtained by NGSadmix should be all presented in Figure S2. All results of K generated by software (e.g. Clumpak) should be presented in the form of figures or text in supplementary files.

3. Three are some problems in the introgression analysis section:

(1) Whether the shares of derived alleles are equilibrium or not were compared in the introgression test calculated by D statistic, as A represents the ancestral alleles and B represents the derived in ABBA-BABA test. Meanwhile, ABBA represents shared derived alleles between P2 and P3, while BABA represents shared derived alleles between P1 and P3. Further, the D statistic do not show direction, such as D is higher than 0 means P2 and P3 shared more derived alleles than P1 and P3, but it is not sure that whether it introgressed from P2 to P3 or from P3 to P2 unless subsequent polarization like Dfoil has been done (Pease, James B.; Hahn, Matthew W. (2015): Detection and Polarization of Introgression in a Five-Taxon Phylogeny. In Systematic biology 64 (4), pp. 651-662. DOI: 10.1093/sysbio/syv023.). Therefore, subsequent judgment on introgression direction needs more proofs, and the arrow in Fig2C should not be one-way.

(2) A single gene flow may cause multiple f4-ratio combinations to be significant, when many species (or populations) were used to calculate the f4-ratio of different subset combinations due to different levels of genetic relationship between these populations, which increased the possibility of false positive. Maybe the f-branch could be used to separate these associated f4-ratio (Malinsky, M., Svardal, H., Tyers, A. M., Miska, E. A., Gener, M. J., Turner, G. F., & Durbin, R. (2018). Whole‐genome sequences of Malawi cichlids reveal multiple radiations interconnected by gene flow. Nature Ecology & Evolution, 457, 830. https://doi.org/10.1038/s41559‐018‐0717‐x).

(3) For the introgressed populations from multiple species (such as Vrip), how to distinguish introgressed fragments from different sources when detecting local introgression areas?

(4) The distribution of fdM should be displayed in a Manhattan plot with corresponding threshold line. Only one histogram is not clear enough.

(5) The calculation of divergence time is needed between populations.

(6) It is necessary to further construct a phylogenetic tree and display the SNP haplotype heatmap for the key functional introgression region.

4. Figure 4B: Only two regions were analyzed further without the region which owns the highest fdM value.

5. The details of GWAS were not presented and there is only a final statistic result. First, the overall Manhattan plot of the GWAS should be added to determine whether the significance of GWAS signal is generated from aggregated results rather than scattered. The significance obtained from such a result may contain too many false positives. Second, the QQ plot is needed to exclude the influence of population stratification effect. Last, Bonferroni correction is recommended for the test of significance when other methods may lead to higher possibility of false positives. And a table should be added in the supplements to record the location of significantly related SNPs and the corrected P values.

6. Line 218: "With this approach, we found bacterial levels were significantly associated (FDR adjusted p < 0.05) with 261 SNPs across the genome (Dataset S3), of which 25% (64/261) were located in pIRs of at least one of the nine trios."

25% of the SNPs related to susceptibility fall into the pIRs, which is a union of "at least one of the nine trios". From Table 1, we can see that the total introgression area has been accounted for more than 40% of the whole genome according to your method. Therefore, a set of non-redundant introgression areas needs to be given in supplements and to show the range of these introgression areas in the set. Then the authors can calculate how many SNPs related to susceptibility fall into the pIRs, then analysis whether the enrichment of introgression regions is significant.

7. Line 235: "The number of candidate SNPs associated with each of the three strongest bioclimatic variables ranged from 3,380 SNPs (for BIO9 in V.candicans) to 39,587 (for BIO11 in V. arizonica) (Dataset S4)." Which SNPs are overlapped between genome environment associations (GEA) and putatively introgressed regions (pIR)? Please provide these SNPs in Dataset S4.

Minor comments:

1. Page 3, line 76: The authors addressed Three sets of questions, but closely followed by Four questions as "First…, Second…, Third…, Finally…". Please make sure the right statements.

2. Figure1, the legend "suceptible" is supposed to be "susceptible".

3. Line 96: "K=1 to 10" should be "K=2 to 10", the K=1 is unnecessary.

4. Line 113: the 'or/or' should it be changed to 'and/or' ?

5. Line 121: What does the abscissa of the Figure 2B represents?

6. Page 5, line 132: A spelling mistake of "andr".

7. Figure 2, the word sizes of "A" and "B" are smaller than "C" and "D". Figure 2D is too small to see it clearly.

8. The authors used "f4-ratio" at Page 5 line 127, and then used "f4" in line 133, 135, 165, 528, 658 and 671, "f statistics" in line 276. Please make sure the subscript right.

9. Line 175 "5 of 9 trios" should be "6 of 9 trios", there are 6 notable trios in the Figure S11.

10. Line 210: "genome-wide associations analyses (GWA)" should be abbreviated as GWAS.

11. Page 9, line 247: The format of reference 39 is not consistent with others.

12. Figure 4, the word sizes of "A", "B", "C", "D" and "E" are too big.

13. Page 15, line 433: A space is required between "100" and "bp".

14. Line 513: "(in red, Figure 1C)", Figure 1C couldn't to be found.

15. Line 533: How to calculate the CLR, the RAiSD software seems to have no CLR algorithm.

16. Some spaces are missed between text and reference, like in line 365 (before the references 64 and 60) and line 563 (before the reference 103). Please check all over this manuscript again.

17. "fd" and "fdM" were used at Page 6 line 159, but in MATERIALS and METHODS section, "fd" and "fdM" were used in line 525, 527, 669 and 670. Please make sure the subscript right.

18. In MATERIALS and METHODS section, ":" were used after the underlined subtitles at the beginning of some paragraphs, but others didn't, like line 477, 529, 543 and 596. Please use consistent format.

**Reviewer 2**

**Are you able to assess all statistics in the manuscript, including the appropriateness of statistical tests used?**

No, I do not feel adequately qualified to assess the statistics.

**Comments to author:**

Introgression among North American wild grapes (Vitis) fuels biotic and abiotic adaptation by Morales-Cruz and colleagues is a well-written and thoughtful manuscript. It takes a large and valuable dataset and thoroughly uses it to address the question of introgression in wild Vitis from numerous angles. Taking the work all the way from reference genome assembly to climate modelling is impressive. Vitis is an excellent model for the study of introgression and I believe these results will be of interest to the broader scientific community, particularly those working on crops and crop wild relatives.

My comments below are relatively minor. My expertise lies in the collection and analysis of the next-generation DNA sequence data for the purposes of population genetic analysis and GWAS. I cannot comment on genome assembly, genome annotation, or climate modelling as these are outside of my area of expertise.

In particular, there are two areas where further information from the authors would be valuable due to potential biases in the work.

First, the authors use a single reference genome from one of wild species (V. arizonica). Aligning other species to this reference for comparison could result in ascertainment bias, since some regions of the genome will not be shared across all Vitis. How does this potential bias influence the downstream analyses and conclusions of the work? I do not think it is necessary for the authors to rerun these analyses, but I would encourage them to articulate in the discussion what the limitations of their work are in this regard.

Secondly, it is unclear from the manuscript and my own unfamiliarity with some of the programs used what minor allele frequency filter, if any, is used. The authors state a MAF filter for NgsAdmix (L504) of 5%, but it is unclear if this implemented for the other analyses. Given that V. monticola is only represented by 6 accessions out of 111 (130 starting samples), the MAF filter is an important consideration in this work, since it would be easy to exclude any SNPs that segregate only in V. monticola if the filter was too strict.

Overall, I think the manuscript is well-written and the authors have done some great work. I have included my minor comments, mostly regarding data visualization, below.

- Figure 1 and Figure S1 are redundant, it is the same figure just with additional information provided (a legend for the colors). The complete figure (S1) should be included in the manuscript, rather than referring the reader to the supplement in order to interpret it. Additionally, the color scheme uses red and green to differentiate regions making it impossible for those with colorblindness to interpret.

- L76P3: It states there are three sets of questions but four are listed (after "Third", there is a "Finally" and additional questions)

- "SNPs were removed if they had more than 75% missing data per individual" - it is unclear what the authors mean. Did they remove if it had an average of more than 75% missing data across all individuals? And if so, is there a reason not to impute the missing data?

- L513P17: " For the densitree (in red, Figure 1C) we divided the genome in 10 Kb" looks like it should be Figure 2B

- It would be helpful to the reader to identify the two geographic locations in Figure 2A for V. girdiana. In the map (Figure 1) it looks like there are three geographic locations for sampling, but the majority of accessions are from one location. Whereas, in Figure 2A, it appears that the accessions are approximately equally divided between two groups.

- The resolution of Figure S3 is very difficult to read

- L138P5: Should be Figure 2B not 3B

- Figure 2D could definitely be "zoomed in" since the area of interest is so tiny and difficult to visualize potential overlap with most of the map not of interest. If the full map is for comparison purposes across all comparisons, it could be included in the supplement along with the remaining maps.

- The figure legends in the supplement (Additional Files 13-15: Figures S7-S9) do not identify what the labels stand for (AGR, GAM, GRM, etc). This information is only found in the legend for Table 1 in the main manuscript.

- L190P7: "which suggest" should be "which suggests"

- Plotting the GWAS results would be helpful to the reader instead of only referring to a supplemental dataset

- L264P9: The authors state that "to date, only one genome has been published from another wild Vitis species". This is no longer the case, see: Patel et al (2020) Horticulture Research for an additional V. riparia genome, Wang et al. (2020) The Plant Journal for Vitis amurensis.

**Reviewer 3**

**Are you able to assess all statistics in the manuscript, including the appropriateness of statistical tests used?**

Yes, and I have assessed the statistics in my report.

**Comments to author:**

The manuscript reports the genome assembly of one wild species of grape and whole-genome re-sequencing of 130 accessions of six grape species native to the Southwestern US. These datasets have been used to identify putative inter-species introgression events and assess factors that contributed to retention of introgressed genomic segments in population. The authors generated impressive set of genomic data that will be of great value for genomic analysis of these agriculturally relevant species and facilitate introduction of beneficial wild relative diversity into this cultivated crop.

Discovery of extensive gene flow among the analyzed taxa supports analyses performed in other major crops. While the authors claim that the number of studies assessing the phenotypic effects of introgression is limited, I would prefer to have this statement toned-down. Though they were not cited by the authors, in some crop studies (wheat, maize, potato), the effects of historic introgression from wild relatives on agronomic traits have been documented. Moreover, in some crops introgression from wild relatives is an important source of diversity for breeding. My feeling is that these aspects of wild relative-crop introgression research need to be better reflected in Introduction and Discussion parts of the manuscript.

Association detected between the enrichment of introgression with adaptive variants and variants linked with resistance to a grape pathogen is quite intriguing. The latter appear to be supported by the enrichment of disease response genes within the introgressed genomic segments. I have some concerns about the results that led to this conclusion. First, it is the low power of the association mapping experimental design that is small in sample size and based on heavily structured population. The authors did not provide evidence that the model used for GWAS effectively controls for the effect of population structure on the detected SNP-trait associations. Comparison of Q-Q plots generated using different models would help here. Second, the authors indicated that many introgression events likely occurred between species that occupied the same geographic area in the distant past. What is the chance that the pathogen used in GWAS was a relevant factor that played role in the retention of introgression in the past? I feel that these concerns need to be addressed in the revised manuscript.

Minor comments:

L. 139: The figure legend should provide more detailed explanation of results presented on Fig. 2D. For example, what are the units that were used to show overlap between species?

L. 189-205: It is not clear how the enrichment for disease response genes was performed and whether the authors have taken in the account the possible confounding effect of genomic distribution of disease response genes, which are often enriched in the high-recombining regions of genomes. Please provide more details in the Results and Methods sections.

L. 253-255: It would be more informative to cite original research.

L. 257-274: Many parts of this paragraph seem to belong to results section rather than to discussion.

**Authors Response**

**Point-by-point responses to the reviewers’ comments:**

Reviewer #1

The authors provided valuable resources to the genetic research and breeding of grapevine by resequencing 130 wild accessions from six Vitis species in the Southwestern United States. They focused on the genome wide introgressions among Vitis species, however, the results of introgression events and the subsequent GWAS analysis are a little vague and lack of detailed proof process. The lack of solid functional genes also make the novelty relatively limited.

*Reply: Thank you for the positive comments. With all due respect, however, we would like to push back on the notion that this study has ‘limited novelty’. We first must state clearly what we believe to be obvious: this study was not designed to identify a single gene or group of genes and explore their function. Instead, it was designed to elucidate evolutionary patterns among a group of agronomically important wild plants. It is among few studies to use full resequencing with population sampling to investigate introgression among a group (rather than just a pair) of agronomically important wild plant taxa. And it is also among the first to use such data to investigate potential functional trends for putatively introgressed regions and to investigate species distribution models to hypothesize about historical opportunities for introgression. In short, we absolutely agree that we did not identify solid functional candidates, but that was not our intent. Instead, we present a rare, multi-layered analysis consisting of genomic analyses, phenotypic and bioclimatic associations, and species distribution modeling across a group of species. We know of no other study that presents this combination of approaches on a group of distinct and economically important wild species.*

Major comments:

1. Line 86-87: "We generated a reference genome of V. arizonica produced from long-read sequences [30] that were assembled with the aid of an optical map."

The reference genome of V. arizonica mentioned here could not to be found in NCBI database. By the way, since the genome of V. arizonica had been reported in the previous paper (as Massonnet M, Cochetel N, Minio A, Vondras AM, Lin J, Muyle A, et al. The genetic basis of sex determination in grapes. Nat Commun. 2020; 11:2902.), the descriptions related to the genome sequencing, assembly and annotation in the manuscript should be removed. For instance, the description "The reference assembly contained 19 anchored pseudomolecules, an N50 of 25.9 Mb, a size of 503 Mb and a BUSCO score of 96.4% (Additional File 2: Table S1 & Additional File 3: Table S2)" will mislead readers.

*Reply: Massonnet et al 2020 published the contigs of Vitis arizonica. In this study, we added Bionano optical mapping data to create scaffolds of the contigs and created a chromosome-scale genome reference. Hence, all of the N50 statistics, etc., are unique to this paper and are completely necessary for this paper. We have kept the statistics in the manuscript. We also retained the gene expression and annotation information, because all of this is all new to this paper, too!*

*Although we believe that the breakdown of information between Massonnet et al. and this paper was clear previously, we have slightly modified the first paragraph of the Results to make it even clearer. We also combed the Methods carefully again to be sure that we were not misleading about what was done for this paper vs. the previous paper. To sum: there is lots of new information in this paper about the genome, including scaffolding with an optical map, annotation, etc., and this information needs to remain in the manuscript.*

*The reviewer is correct that the genome is not on NCBI. The genome and data are available from http://www.grapegenomics.com/. This site also includes a genome browser. To further make the data available that we have used in this paper, we have submitted the arizonica assembly and annotations to Zenodo, which has generated a doi that is generally available to the public. The Zenodo doi (https://doi.org/10.5281/zenodo.4977234) is reported in the ms. We have not, however, submitted the genome to NCBI, and this is quite purposeful. The reason is that NCBI generates new genome versions that complicate public use by offering multiple versions and annotations based on different standards. Please also note that in our experience genomes need not be submitted to NCBI to be published by the Springer Nature group, so long as the data are publicly available.*

In addition, in line 630, the raw data (BioProject ID: PRJNA607282 & PRJNA705722) could not be retrieved in NCBI.

*Reply: Yes, those BioProjects were not intended to be public until publication. The raw sequencing data are now available under PRJNA731597 and the IsoSeq data are in PRJNA705722.*

2. Line 95-96: The K=2 to 10 clusters of genetic structure obtained by NGSadmix should be all presented in Figure S2. All results of K generated by software (e.g. Clumpak) should be presented in the form of figures or text in supplementary files.

*Reply: We added the admixture plots from K=2 to K=10 as Additional File 2: Figure S1.*

3. Three are some problems in the introgression analysis section: (1) Whether the shares of derived alleles are equilibrium or not were compared in the introgression test calculated by D statistic, as A represents the ancestral alleles and B represents the derived in ABBA-BABA test. Meanwhile, ABBA represents shared derived alleles between P2 and P3, while BABA represents shared derived alleles between P1 and P3. Further, the D statistic do not show direction, such as D is higher than 0 means P2 and P3 shared more derived alleles than P1 and P3, but it is not sure that whether it introgressed from P2 to P3 or from P3 to P2 unless subsequent polarization like Dfoil has been done (Pease, James B.; Hahn, Matthew W. (2015): Detection and Polarization of Introgression in a Five-Taxon Phylogeny. In Systematic biology 64 (4), pp. 651-662. DOI: 10.1093/sysbio/syv023.). Therefore, subsequent judgment on introgression direction needs more proofs, and the arrow in Fig2C should not be one-way.

*Reply: We thank the reviewer profusely for these comments, because he/she is absolutely correct about the issue of directionality. In response, we have made changes to avoid referring to the direction of introgressions. To do so, we removed mention of “receptor” and “donor” populations, but now refer to the species as the position (either P2 or P3) used in the ABBA-BABA tree models. We have also removed the previous Figure 2C and exchanged it for the tree models of the nine significant trios. This replacement helps better define the trios (as requested by Reviewer 2) and illustrates that we focus on introgressions between the P2 and P3 populations (again, without a specific direction). We also added arrows in both directions for Figure 4A&D. However, it is important to note that these revisions do not alter our primary conclusions, which were frankly agnostic to direction.*

*Thank you for the suggestion to use Dfoil, but it is not appropriate for our data for two reasons. First, this method was developed to apply to the genome assembly of one individual per species to calculate the D-statistic. Given that our dataset is composed of multiple (6-24) individuals per species and re-sequencing data (as opposed to genome assemblies), we would require a method that adequately takes the population allele frequency within each species. And second, the tree topology for the 5-taxon test requires two pairs of closely related species, in addition to the outgroups. This requirement excludes several of our significant introgression events. For example, it excludes tests that include V. candicans.*

*To sum: we are very grateful for the reviewer’s comment about directionality, as it was fully correct. We have carefully removed references to directionality, but this does not alter our conclusions.*

(2) A single gene flow may cause multiple f4-ratio combinations to be significant, when many species (or populations) were used to calculate the f4-ratio of different subset combinations due to different levels of genetic relationship between these populations, which increased the possibility of false positive. Maybe the f-branch could be used to separate these associated f4-ratio (Malinsky, M., Svardal, H., Tyers, A. M., Miska, E. A., Gener, M. J., Turner, G. F., & Durbin, R. (2018). Whole‐genome sequences of Malawi cichlids reveal multiple radiations interconnected by gene flow. Nature Ecology & Evolution, 457, 830. [https://doi.org/10.1038/s41559‐018‐0717‐x](https://doi.org/10.1038/s41559%E2%80%90018%E2%80%900717%E2%80%90x)).

*Reply: In theory, the advantage of the f-branch test is that it can identify introgression events that can be attributed to an internal branch, which (as the reviewer notes) can reduce the number of identified events. We calculated the f-branch statistic (fb), as requested by the reviewer. We did not observe any significant fb values in any of the internal branches as shown in Additional File 2: Figure S4. These results suggest that the introgression events found in individual species are not significantly correlated, as one would expect if introgression events occurred on an internal branch. That said, we know nothing about the power of this test (we frankly have our doubts about it, if we’re honest), and it is also genome wide, which means it lacks details about genomic regions. Note that despite the fb results, some putatively introgressed regions overlap among species (trios), so we continue to suggest in the Discussion that some of the introgression events could have been ancient.*

(3) For the introgressed populations from multiple species (such as Vrip), how to distinguish introgressed fragments from different sources when detecting local introgression areas?

*Reply: We’re not exactly sure what is being asked here, because each pIR inference is made in the context of a pairwise comparison. So, by definition any identified pIR is defined as between two species (or sources).*

(4) The distribution of fdM should be displayed in a Manhattan plot with corresponding threshold line. Only one histogram is not clear enough.

*Reply: Thank you for this comment. We had one example already in the main text, but we have now added the Manhattan plots of the fdM windows in all the trios as Additional File 2: Figure S11.*

(5) The calculation of divergence time is needed between populations.

*Reply: Thank you for this comment. While we view the estimate of divergence time as not necessary and generally outside the main goals of the paper, we were happy to conform to the reviewer’s request. To do so, we created a clock calibrated phylogenetic tree using BEAST. We used the estimate from Wan et al 2013 (https://pubmed.ncbi.nlm.nih.gov/23826735/) for the separation of Muscadinia and the Vitis subgenera. We added the clock calibrated tree as Additional File 2: Figure S3 and added a description of the analysis to the Methods.*

(6) It is necessary to further construct a phylogenetic tree and display the SNP haplotype heatmap for the key functional introgression region.

*Reply: With apologies, we did not quite understand what was being requested here. However, given the mention of a phylogenetic tree, we inferred that the reviewer wanted us to investigate taxonomic relationships within pIRs. We have done that for each of the trios and presented the results in Additional File 2: Figure S14. It shows that within pIRs, the favored consensus tree is reversed for P2 vs. P3, which is wholly consistent with the inference that these regions have a history of introgression. We believe the addition of this analysis substantively strengthens the manuscript and appreciate the comment (with apologies again if we misunderstood it).*

4. Figure 4B: Only two regions were analyzed further without the region which owns the highest fdM value.

*Reply: Thanks for this comment. But we need to be clear: we did not analyze only two regions! The figure provides examples to help illustrate one fundamental finding, which is that our inferred pIRs are heavily enriched for resistance genes. The examples in the figure are complemented by full-genome analyses across trios that are described in detail in the text. As it is not feasible to present similar diagrams for all of the inferred pIRs, that leaves us with two options: to either forego any examples (i.e., like those we have in the figure) or to retain the existing (or similar) examples to help illustrate the phenomenon. We have opted for the latter and retain the examples in the figure.*

5. The details of GWAS were not presented and there is only a final statistic result. First, the overall Manhattan plot of the GWAS should be added to determine whether the significance of GWAS signal is generated from aggregated results rather than scattered. The significance obtained from such a result may contain too many false positives. Second, the QQ plot is needed to exclude the influence of population stratification effect. Last, Bonferroni correction is recommended for the test of significance when other methods may lead to higher possibility of false positives. And a table should be added in the supplements to record the location of significantly related SNPs and the corrected P values.

*Reply: Both reviewers 1 and 3 were concerned by the potential influence that genetic structure may have on the identification of outlier SNPs and the potential presence of false positives. In the previous version we acknowledged that the GWAS could be biased by population structure, and we carefully conditioned our conclusions accordingly. However, we agree with both reviewers that more GWAS analyses and details (including the Manhattan plot, histogram of p-values, QQ plots) are helpful. We have therefore modified the GWAS in many ways, but we must also accentuate an important point that we had hoped was obvious in the original manuscript: this study was not designed to find a single (or set) of genes for functional characterization! We are asking a very different and far more general question: are pIRs statistically overrepresented for associated SNPs, suggesting that pIRs have some functional basis? GWAS is not the main point of the manuscript, but rather (in our view) simply a way to further explore the fact that pIRs are enriched for defense-related genes.*

*Manhattan plot: As requested, we have added a Manhattan plot to the supporting information (Additional File 2: Figure S17). The outlier SNPs are scattered across the genome, but in some regions we see some aggregations within chromosomes (i.e., chromosomes 1, 3, 5, 7, 11, 14, 15, 18). However, we did not expect to find strong aggregation because we are only analyzing SNPs without missing data that are shared between species. This results in a reduced but highly filtered dataset (~300K SNPs vs 5M+ SNPs).*

*FDR vs Bonferroni corrections: We appreciate that the reviewer would like us to apply Bonferroni corrections, but we believe it is not only unnecessary but does not conform to the precedence in the literature. Bonferroni correction can, admittedly, reduce the presence of false positives, but it likely also reduces the power of the analyses and removes true positives. Since we are using shared SNPs without missing data, we have already used strict criteria to ensure that only the highest quality data are used. This approach also limits the number of SNPs, and we believe adding Bonferroni corrections are too stringent for our analyses. [We could increase the number of SNPs by retaining SNPs with missing data, but the imputation of missing data can also increase the number of false positives.*

*More importantly, the general precedent in the literature is to use FDR (rather than Bonferroni corrections) with these methods. We reviewed all the papers that have used the LFMM2 method (Caye et al. 2020: https://pubmed.ncbi.nlm.nih.gov/30657943/) and 83% of these papers (24/29) used FDR (and 16 of the 24 used an FDR<0.05, which is what we have applied). Given that we are: i) not trying to pinpoint the causal genes that could contribute to PD resistance but instead documenting genomic trends, ii) using a limited number of high-quality SNPs, iii) that contrary to the reviewer’s comment, most studies use the FDR, and iv) readily acknowledging the limitations of our analyses, we believe that using FDR is not only an adequate choice but the appropriate choice.*

*QQ plots, and comparing different models: Both reviewer 1 and 3 suggested that we add the QQ plots to estimate the impact of genetic structure. Moreover, reviewer 3 suggested that we compare different models to test the impact of genetic structure. These are valuable suggestions, and we are happy to comply.*

*To recap: based on a PCA and a Screen plot, we have shown that there are 7 genetic clusters (Additional File 2: Figure S15). We also compared three GWAS models: a simple linear regression model (as in Cubry et al. 2020 [https://pubmed.ncbi.nlm.nih.gov/32936396/]; Rhoné et al. 2020 [https://pubmed.ncbi.nlm.nih.gov/33077747/]; and Luo et al. 2021 [https://pubmed.ncbi.nlm.nih.gov/33217107/]), a LFMM model with 7 Latent Factors (LFs), corresponding to the seven clusters, without using genomic control (GIF option in the lfmm_test function), and a LFMM with 7 LF using a genomic control. We found that controlling for genetic structure with LFs and using a genomic control reduced extreme p-values based on the Q-Q plot, suggesting better control for genetic structure (Additional File 2: Figure S16).*

*However, even after using GIF to control for genetic structure, we found that the QQ plots show that the observed -loq10(p) values are above the slope=1 line as expected when there is signal in the analyses. We also compared our QQ plots with other studies that have used LFMM2. From ~30 papers that have used LFMM2, only 5 reported QQ plots, so it seems as if this is by no means a requirement for publication. In general, the QQ plots presented in other intraspecific studies have similar QQ plot distributions (Figure S5a in Rhoné et al. 2020; Figure 3 in Zhang et al. 2019 [https://pubmed.ncbi.nlm.nih.gov/31138268/]; Figure 4 right panel in Luo et al. 2020) or have variable with similar distributions (Cubry et al. 2020: Figure S8_T_tmacPC2, DFT2012a, T_DFT2021b, TRL2012, T_SBL2012, T_SBintL2012). Overall, these analyses suggest that our GWAS is adequate, given our goal to establish trends while being appropriately cautious with our conclusions.*

*It is nonetheless possible that adding an additional number of LFs better corrects for genetic structure, and so we explored models with variable numbers of LFs (Additional File 2: Figure S18). We compared the LR model against LFMM2 models using from 6 to 12 LFs. We found that increasing the number LFs up to 8 reduced the number of significant SNPs, which may reflect better control for population structure but almost certainly also reduces the power of the analyses (as reported previously by Luo et al. 2021). With 8 LF, we found 129 outlier SNPs; there were not significantly enriched in pIRs but up to 10% of outlier SNPs were found in pIRs in some trios. We now report the results with LF=8 in the text, to be fully transparent. However, we continue to believe that the only reasonable evidence to guide the number of LFs for analysis is based on the seven identified genetic clusters, and so we continue to report these results. Moreover, if LF=7 results produce many false positives, it is hard for us to imagine why these statistical artifacts should be enriched within pIRs.*

6. Line 218: "With this approach, we found bacterial levels were significantly associated (FDR adjusted p < 0.05) with 261 SNPs across the genome (Dataset S3), of which 25% (64/261) were located in pIRs of at least one of the nine trios."25% of the SNPs related to susceptibility fall into the pIRs, which is a union of "at least one of the nine trios". From Table 1, we can see that the total introgression area has been accounted for more than 40% of the whole genome according to your method. Therefore, a set of non-redundant introgression areas needs to be given in supplements and to show the range of these introgression areas in the set. Then the authors can calculate how many SNPs related to susceptibility fall into the pIRs, then analysis whether the enrichment of introgression regions is significant.

*Reply: We apologize if our explanation of approaches somehow misled the reviewer. Yes, if one sums the % pIRs among trios in Table 1 they sum to approximately 40%, but (as the reviewer notes) it would be completely wrong to use that value in our analysis across all species; one needs to look at the non-redundant set of pIRs if analyzing across all species. Accordingly, we did not use tha value. However, because all of the pIR analyses are focused on trios, we thought (and continue to think) that enrichment analyses should be done at the level of individual trios, and that is exactly what we did. We modified the text slightly to make it clearer, but we continue to report the analyses based on trios.*

7. Line 235: "The number of candidate SNPs associated with each of the three strongest bioclimatic variables ranged from 3,380 SNPs (for BIO9 in V.candicans) to 39,587 (for BIO11 in V. arizonica) (Dataset S4)." Which SNPs are overlapped between genome environment associations (GEA) and putatively introgressed regions (pIR)? Please provide these SNPs in Dataset S4.

*Reply: Because a species was associated with more than one trio, adding the information to Dataset S4 was not ideal. However, we created Dataset S5 in which we present only SNPs that were inside pIRs and were significantly associated with the top 3 bioclimatic variables (BF > 10).*

Minor comments:

1. Page 3, line 76: The authors addressed Three sets of questions, but closely followed by Four questions as "First…, Second…, Third…, Finally…". Please make sure the right statements.

*Reply: Thank you for this comment. We changed the word “three” to “four”. The sentence now reads “Given this multifaceted dataset, we address four sets of questions”.*

2. Figure1, the legend "suceptible" is supposed to be "susceptible".

*Reply: Thank you. We changed the word to “susceptible”.*

3. Line 96: "K=1 to 10" should be "K=2 to 10", the K=1 is unnecessary.

*Reply: Yes, many thanks. The sentence now reads “…used them to assess genetic structure using NGSadmix[31] with K=2 to 10 clusters”.*

4. Line 113: the 'or/or' should it be changed to 'and/or' ?

*Reply: We changed it to “and/or”.*

5. Line 121: What does the abscissa of the Figure 2B represents?

*Reply: We have added an explanation to the figure legend.*

6. Page 5, line 132: A spelling mistake of "andr".

*Reply: Thanks. We removed the letter “r”.*

7. Figure 2, the word sizes of "A" and "B" are smaller than "C" and "D". Figure 2D is too small to see it clearly.

*Reply: Thanks for catching this problem. We fixed the letters sizes of the panels and made Figure 2D bigger.*

8. The authors used "f4-ratio" at Page 5 line 127, and then used "f4" in line 133, 135, 165, 528, 658 and 671, "f statistics" in line 276. Please make sure the subscript right.

*Reply: Thank you for catching this typographical error. We changed everything to f4-ratio.*

9. Line 175 "5 of 9 trios" should be "6 of 9 trios", there are 6 notable trios in the Figure S11.

*Reply: Thank you for catching this typographical error! We changed it to “6 of 9 trios”.*

10. Line 210: "genome-wide associations analyses (GWA)" should be abbreviated as GWAS.

*Reply: We changed to “GWAS” here and after.*

11. Page 9, line 247: The format of reference 39 is not consistent with others.

*Reply: We changed the format of the citation, with many thanks.*

12. Figure 4, the word sizes of "A", "B", "C", "D" and "E" are too big.

*Reply: We reduced the sizes of the letters.*

13. Page 15, line 433: A space is required between "100" and "bp".

*Reply: We added a space between “100” and "bp”.*

14. Line 513: "(in red, Figure 1C)", Figure 1C couldn't to be found.

*Reply: We changed it to “Figure 2B”.*

15. Line 533: How to calculate the CLR, the RAiSD software seems to have no CLR algorithm.

*Reply: We changed all instances of CLR to the µ throughout the manuscript, including Figure 3B. Thank you for catching this.*

16. Some spaces are missed between text and reference, like in line 365 (before the references 64 and 60) and line 563 (before the reference 103). Please check all over this manuscript again.

*Reply: We added spaces between the text and reference.*

17. "fd" and "fdM" were used at Page 6 line 159, but in MATERIALS and METHODS section, "fd" and "fdM" were used in line 525, 527, 669 and 670. Please make sure the subscript right.

*Reply: We changed when it was necessary to subscript. Thank you for catching this.*

18. In MATERIALS and METHODS section, ":" were used after the underlined subtitles at the beginning of some paragraphs, but others didn't, like line 477, 529, 543 and 596. Please use consistent format.

*Reply: We added “:” to all of them. Thank you!*

Reviewer #2

Introgression among North American wild grapes (Vitis) fuels biotic and abiotic adaptation by Morales-Cruz and colleagues is a well-written and thoughtful manuscript. It takes a large and valuable dataset and thoroughly uses it to address the question of introgression in wild Vitis from numerous angles. Taking the work all the way from reference genome assembly to climate modelling is impressive. Vitis is an excellent model for the study of introgression and I believe these results will be of interest to the broader scientific community, particularly those working on crops and crop wild relatives.

*Reply: Thank you for these positive comments!*

My comments below are relatively minor. My expertise lies in the collection and analysis of the next-generation DNA sequence data for the purposes of population genetic analysis and GWAS. I cannot comment on genome assembly, genome annotation, or climate modelling as these are outside of my area of expertise. In particular, there are two areas where further information from the authors would be valuable due to potential biases in the work.

Major comments: First, the authors use a single reference genome from one of wild species (V. arizonica). Aligning other species to this reference for comparison could result in ascertainment bias, since some regions of the genome will not be shared across all Vitis. How does this potential bias influence the downstream analyses and conclusions of the work? I do not think it is necessary for the authors to rerun these analyses, but I would encourage them to articulate in the discussion what the limitations of their work are in this regard.

*Reply: We added a few sentences about reference bias to the second paragraph of the Discussion. It is very difficult to know the effect of reference bias on any study (this or any other population genomic study!), but some studies suggest that the effect is not very strong for some inferences. It is worth pointing out that it isn’t entirely clear how to proceed if more genomes were available, and we make that point in the Discussion as well. Overall, we are grateful for this comment, because it is an issue worth bearing in mind.*

*Secondly, it is unclear from the manuscript and my own unfamiliarity with some of the programs used what minor allele frequency filter, if any, is used. The authors state a MAF filter for NgsAdmix (L504) of 5%, but it is unclear if this implemented for the other analyses. Given that V. monticola is only represented by 6 accessions out of 111 (130 starting samples), the MAF filter is an important consideration in this work, since it would be easy to exclude any SNPs that segregate only in V. monticola if the filter was too strict.*

*We did not use MAF filters any of the analyses. However, we did test the GWAS with a MAF <0.05 filter and without filtering. The results did not change, and we now mention that fact in the Methods section.*

Minor comments:

Overall, I think the manuscript is well-written and the authors have done some great work. I have included my minor comments, mostly regarding data visualization, below.

- Figure 1 and Figure S1 are redundant, it is the same figure just with additional information provided (a legend for the colors). The complete figure (S1) should be included in the manuscript, rather than referring the reader to the supplement in order to interpret it. Additionally, the color scheme uses red and green to differentiate regions making it impossible for those with colorblindness to interpret.

*Reply: We decided to remove the internal colors of the map to avoid confusion, and as a consequence supplementary figure S1 became redundant and was removed.*

- L76P3: It states there are three sets of questions but four are listed (after "Third", there is a "Finally" and additional questions).

*Reply: Thanks for catching this. We changed the word “three” to “four”.*

- "SNPs were removed if they had more than 75% missing data per individual" - it is unclear what the authors mean. Did they remove if it had an average of more than 75% missing data across all individuals? And if so, is there a reason not to impute the missing data?

*Reply: We removed SNPs that had more than 25% missing data across all samples and species. To clarify this, we edited this section as follows: “We kept SNPs sites for downstream analysis if they were biallelic, had quality higher than 30, had a depth of coverage higher than five reads, also had no more than three times the median coverage depth across accessions, and had less than 25% of missing data among all samples”. Most of the analyses in this study used allele frequencies of each separate species, we expect the percentage of missing data within each species to be significantly lower. Moreover, we did not perform imputation to avoid introducing errors into our analyses.*

- L513P17: " For the densitree (in red, Figure 1C) we divided the genome in 10 Kb" looks like it should be Figure 2B.

*Reply: Thanks for the good catch! We changed it to Figure 2B.*

- It would be helpful to the reader to identify the two geographic locations in Figure 2A for V. girdiana. In the map (Figure 1) it looks like there are three geographic locations for sampling, but the majority of accessions are from one location. Whereas, in Figure 2A, it appears that the accessions are approximately equally divided between two groups.

*Reply: We now show two shapes for V. girdiana in Figure 1 to better show the location of the two genetic clusters. We also present the coordinates for each sample in Additional File 1: Table S3. We hope these address the comment thoroughly.*

- The resolution of Figure S3 is very difficult to read.

*Reply: We increased the quality of the figure, which is now Additional File 2: Figure S2.*

- L138P5: Should be Figure 2B not 3B.

*Reply: We changed this to “Figure 2C”.*

- Figure 2D could definitely be "zoomed in" since the area of interest is so tiny and difficult to visualize potential overlap with most of the map not of interest. If the full map is for comparison purposes across all comparisons, it could be included in the supplement along with the remaining maps.

*Reply: In retrospect, we fully agree, so we “zoomed in” in both maps of Figure 2D to better show the SDMs. We think it greatly improves the presentation, so thanks!*

- The figure legends in the supplement (Additional Files 13-15: Figures S7-S9) do not identify what the labels stand for (AGR, GAM, GRM, etc). This information is only found in the legend for Table 1 in the main manuscript.

*Reply: We added the information to the supplemental files the reviewer suggested, now Figures S8 to S12. We now also show explicitly the tree models in Figure 2C, which we believe helps make the rest of the paper easier to digest.*

- L190P7: "which suggest" should be "which suggests".

*Reply: Thanks. We fixed the typo. The sentence now reads “which suggests”.*

- Plotting the GWAS results would be helpful to the reader instead of only referring to a supplemental dataset.

*Reply: Thank you for this suggestion, we have added the Manhattan plot and have performed multiple evaluations (Figures S15-S18), as detailed above in the response to reviewer 1.*

- L264P9: The authors state that "to date, only one genome has been published from another wild Vitis species". This is no longer the case, see: Patel et al (2020) Horticulture Research for an additional V. riparia genome, Wang et al. (2020) The Plant Journal for Vitis amurensis.

*Reply: Thank you for the comment! We added the following sentence about other published genomes to the discussion section: “To date, three genomes have been published from other wild Vitis species, two from V. riparia [52,53] and one genome from V. amurensis [54]”.*

Reviewer #3

The manuscript reports the genome assembly of one wild species of grape and wholegenome re-sequencing of 130 accessions of six grape species native to the Southwestern US. These datasets have been used to identify putative inter-species introgression events and assess factors that contributed to retention of introgressed genomic segments in population. The authors generated impressive set of genomic data that will be of great value for genomic analysis of these agriculturally relevant species and facilitate introduction of beneficial wild relative diversity into this cultivated crop.

*Reply: Thank you for these positive comments.*

Major comments:

Discovery of extensive gene flow among the analyzed taxa supports analyses performed in other major crops. While the authors claim that the number of studies assessing the phenotypic effects of introgression is limited, I would prefer to have this statement toned-down. Though they were not cited by the authors, in some crop studies (wheat, maize, potato), the effects of historic introgression from wild relatives on agronomic traits have been documented. Moreover, in some crops introgression from wild relatives is an important source of diversity for breeding. My feeling is that these aspects of wild relative-crop introgression research need to be better reflected in Introduction and Discussion parts of the manuscript.

*Reply: Thank you for making this point. This was our error in the presentation. We of course 100% agree that introgression between a crop and its wild relatives is an important topic that has been studied already and needs to be studied further. But we are not studying it here. Moreover, crop-wild introgression studies are often focused on a comparison between the crop and a single wild relative. We had intended to make the point that there have been very few papers that focus on introgression among multiple wild relatives of a crop - i.e., the natural history of introgression in a group of wild species that have agronomic utility. We continue to believe this statement to be true. However, the point may not have been clear and is perhaps splitting hairs in any case.*

*To conform to the reviewer’s request, we have added some text and citations to the second paragraph of the Introduction and also (very briefly) to the first paragraph of the Discussion. To be honest, we are leery of trending too far down the rabbit hole of wildcrop introgression, because it is not our major interest here. We fear potentially altering our focus (introgression among wild species) to something quite different (crop-wild introgression mediated by artificial selection). We nonetheless hope that these changes meet the spirit of the reviewer’s comment.*

Association detected between the enrichment of introgression with adaptive variants and variants linked with resistance to a grape pathogen is quite intriguing. The latter appear to be supported by the enrichment of disease response genes within the introgressed genomic segments. I have some concerns about the results that led to this conclusion. First, it is the low power of the association mapping experimental design that is small in sample size and based on a heavily structured population. The authors did not provide evidence that the model used for GWAS effectively controls for the effect of population structure on the detected SNP-trait associations. Comparison of Q-Q plots generated using different models would help here. Second, the authors indicated that many introgression events likely occurred between species that occupied the same geographic area in the distant past. What is the chance that the pathogen used in GWAS was a relevant factor that played a role in the retention of introgression in the past? I feel that these concerns need to be addressed in the revised manuscript.

*Reply: We appreciate these suggestions. These concerns were also pointed out by reviewer 1; please feel free to peruse our lengthier response to that reviewer. But briefly: In this new version of the manuscript, we have added multiple tests to evaluate the impact of genetic structure (Figures S15-S18). As you suggest, we have also compared different models to identify how genetic structure could inflate the number of false positives. Briefly, we compared a simple linear regression model (as in Curby et al. 2020, Rhoné et al. 2020 and Luo et al. 2020), and two LFMM models using 7 LF with and without a genomic control (GIF option in the lfmm_test fuction). We found that using LFMM2 and the genomic control reduced the impact of genetic structure (Figure S16), as inferred from the distribution of extreme p-values in a QQ plot.*

*Since we found that there is still an impact of genetic structure, we compared the QQ plots with other studies that have reported QQ plots. Please note that from the ~30 papers that have used LFMM2, only 5 reported QQ plots. Importantly, we found that our plots show similar distributions with papers using intraspecific studies (Rhoné et al. 2020- Figure S5a [https://pubmed.ncbi.nlm.nih.gov/33077747/]; Zhang et al. 2019- Figure 3 [https://pubmed.ncbi.nlm.nih.gov/31138268/]; Luo et al. 2020-Figure 4 right panel [https://pubmed.ncbi.nlm.nih.gov/33217107/]; Cubry et al. 2020 -Figure S8_T_tmacPC2, DFT2012a, T_DFT2021b, TRL2012, T_SBL2012, T_SBintL2012 [https://pubmed.ncbi.nlm.nih.gov/32936396/]). However, we also tested the effect of increasing the number of LFs to control for more genetic structure (Additional File 2: Figure S18). While this might reduce biases associated with genetic structure, it also likely reduces the power of the analyses (Luo et al. 2020). Indeed, we found that increasing the number of LF to 8 substantively reduced the number of outlier SNPs, with relatively little shift for LF > 8. At LF=8, outlier SNPs were no longer significantly enriched in pIRs regions (although the trend was in the right direction). We now report this result in the text, but we also note that we cannot know if the loss of enrichment is due to reduced power of the analyses with more LFs or the removal of false positives. We have to note again that the only reasonable number of LFs, based on the a priori clustering analysis, is the one we used - i.e., LF = 7. And if LF=7 is generating many false positives, it is hard to imagine why those false positives should be enriched within pIRs.*

*Overall, our results suggest that introgression has preferentially involved diseaserelated genes, and that the GWAS provides some positive (but limited) evidence that there is a functional basis for the retention of introgressed resistance genes. However, we continue to try to appropriately condition our responses and be as transparent as possible, especially seeing as we do not view the GWAS as the major point of the study.*

*Again, many thanks for the suggestions regarding GWAS analyses! We certainly welcome further comments.*

Minor comments:

L. 139: The figure legend should provide more detailed explanation of results presented on Fig. 2D. For example, what are the units that were used to show overlap between species?

*Reply: We modified Figure 2D to better show overlaps. We also added this to the Figure Legend: “The overlap corresponds to the number of overlapped pixels from the raster objects at a 2.5 arcseconds.”*

L. 189-205: It is not clear how the enrichment for disease response genes was performed and whether the authors have taken in the account the possible confounding effect of genomic distribution of disease response genes, which are often enriched in the high-recombining regions of genomes. Please provide more details in the Results and Methods sections.

*Reply: This is a fair and interesting question, because the enrichment of disease genes and recombination could be confounded. To investigate this question, we broke the genome into four quadrants of high, medium, low and no recombination. We then asked if disease genes are enriched in any of the four quadrants. The answer is that LRRs are significantly enriched in regions of LOW recombination in our genome. Hence, the pIRs are exceptions by being enriched for disease genes AND being in regions of high recombination.*

*We have included this result briefly in the Results section and thank you for the question.*

L. 253-255: It would be more informative to cite original research.

*Reply: We have cited more original research in the Introduction. We also expanded this section somewhat to cite an additional review. Again, we are a bit loath to focus on crop-wild introgression too extensively, but if there is specific research that the reviewer feels must be cited, we would be happy to hear about it.*

L. 257-274: Many parts of this paragraph seem to belong to results section rather than to discussion.

*Reply: We do not disagree with this comment, but we opted not only to retain but to expand this section of the Discussion, for two reasons. One is that one reviewer was under the misimpression that the V. arizonica genome analyses were all previously published, which is not true at all. Hence, we want to continue to discuss this newly assembled and annotated reference. Second, reviewer 2 wanted us to comment on reference biases, and this seemed to be the most logical place to include those comments.*

**Second round of review**

**Reviewer 1**

The authors have solved almost all of the questions.

There is only a minor issue in the GWAS part: the display format of Manhattan plot (Additional File 2: Figure S17) needs to be revised. It is recommended to refer to the Fig. 2 of the following paper (Al Kalaldeh M, Gibson J, Lee SH, et al. Detection of genomic regions underlying resistance to gastrointestinal parasites in Australian sheep[J]. Genetics Selection Evolution, 2019, 51(1): 1-18.).

**Reviewer 2**

Please note that in the list of other published Vitis genomes, an additional one has come to my attention since my initial review, the 'Concord' reference genome (Yang et al. (2020) Horticulture Research) which is primarily V. labrusca but does include approximately 1/3 V. vinifera parentage. To ensure the sentence in the manuscript regarding Vitis genomes is comprehensive, this should likely be cited as well.

**Reviewer 3**

Most of my comments are properly addressed.

Based on additional analyses performed by the authors, I still have feeling that the results of GWAS are not solid enough to support claim that selection for resistance was one of the driving forces behind retention of introgression. However, even with these issues, publication of this study could spark some interesting follow up research in other crops to investigate the role of selection for disease resistance in retention of introgressed diversity.

**Authors Response**

**Point-by-point responses to the reviewers’ comments:**

Reviewer #1

The authors have solved almost all of the questions. There is only a minor issue in the GWAS part: the display format of Manhattan plot (Additional File 2: Figure S17) needs to be revised. It is recommended to refer to the Fig. 2 of the following paper (Al Kalaldeh M, Gibson J, Lee SH, et al. Detection of genomic regions underlying resistance to gastrointestinal parasites in Australian sheep[J]. Genetics Selection Evolution, 2019, 51(1): 1-18.).

*Reply: THIS HAS BEEN DONE.*

Reviewer #2

The authors of the manuscript “Introgression among North American wild grapes (Vitis) fuels biotic and abiotic adaptation” have done a thorough job responding to reviewer comments. In particular, I think they have adequately addressed my own comments from the previous review and I believe the manuscript is suitable for publication in its current form.

*Reply: THE CITATION HAS BEEN ADDED.*
